# Accuracy and reliability of forensic handwriting comparisons

**DOI:** 10.1073/pnas.2119944119

**Published:** 2022-08-01

**Authors:** R. Austin Hicklin, Linda Eisenhart, Nicole Richetelli, Meredith D. Miller, Peter Belcastro, Ted M. Burkes, Connie L. Parks, Michael A. Smith, JoAnn Buscaglia, Eugene M. Peters, Rebecca Schwartz Perlman, Jocelyn V. Abonamah, Brian A. Eckenrode

**Affiliations:** ^a^Noblis, Inc., Reston, VA 20191;; ^b^Federal Bureau of Investigation, Laboratory Division, Questioned Document Unit, Quantico, VA 22135;; ^c^Meredith DeKalb Miller, Green Cove Springs, FL 32043;; ^d^Federal Bureau of Investigation, Laboratory Division, Research and Support Unit, Quantico, VA 22135;; ^e^Ideal Innovations, Inc., Arlington, VA 22203

**Keywords:** forensics, handwriting, decision analysis, documents, error rates

## Abstract

Forensic handwriting examination involves the comparison of writing samples by forensic document examiners (FDEs) to determine whether or not they were written by the same person. Here we report the results of a large-scale study conducted to assess the accuracy and reliability of handwriting comparison conclusions. Eighty-six practicing FDEs each conducted up to 100 handwriting comparisons, resulting in 7,196 conclusions on 180 distinct comparison sets, using a five-level conclusion scale. Erroneous “written by” conclusions (false positives) were reached in 3.1% of the nonmated comparisons, while 1.1% of the mated comparisons yielded erroneous “not written by” conclusions (false negatives). False positive rates were markedly higher for nonmated samples written by twins (8.7%) compared to nontwins (2.5%). Notable associations between training and performance were observed: FDEs with less than 2 y of formal training generally had higher error rates, but they also had higher true positive and true negative rates because they tended to provide more definitive conclusions; FDEs with at least 2 y of formal training were less likely to make definitive conclusions, but those definitive conclusions they made were more likely to be correct (higher positive predictive and negative predictive values). We did not observe any association between writing style (cursive vs. printing) and rates of errors or incorrect conclusions. This report also provides details on the repeatability and reproducibility of conclusions, and reports how conclusions are affected by the quantity of writing and the similarity of content.

## Significance

Forensic handwriting examinations are a critical part of the criminal justice system, seeking to determine whether handwritten documents can be attributed to specific writers by comparison to known exemplars. This paper summarizes a 5-y research study designed to assess the accuracy and reliability of forensic handwriting comparison decisions, which is important in assessing scientific validity for admissibility in court. Here we report the results of the largest-scale study yet conducted to measure the accuracy, reproducibility, and repeatability of conclusions made by practicing forensic document examiners when comparing samples selected to span a range of quality, quantity, and attributes found in casework.

Forensic science is under scrutiny, particularly for pattern-based disciplines in which source conclusions are reported. The National Research Council report *Strengthening Forensic Science in the United States: A Path Forward* (1) stated that “The scientific basis for handwriting comparisons needs to be strengthened” and noted that “there has been only limited research to quantify the reliability and replicability of the practices used by trained document examiners.” The President’s Council of Advisors on Science and Technology (PCAST) report *Forensic Science in Criminal Courts: Ensuring Scientific Validity of Feature-Comparison Methods* (2) expressed concerns regarding the validity and reliability of conclusions made by forensic examiners, and called for empirical testing: “The only way to establish the scientific validity and degree of reliability of a subjective forensic feature-comparison method—that is, one involving significant human judgment—is to test it empirically by seeing how often examiners actually get the right answer. Such an empirical test of a subjective forensic feature-comparison method is referred to as a ‘black-box test.’” The National Commission on Forensic Science also called for such testing (3). Although the accuracy and reliability of conclusions made by forensic document examiners (FDEs) have been the focus of multiple studies over the years (4–10), the designs of those studies are notably different from this study (and from PCAST’s recommendations), and therefore the resulting rates are not directly comparable (in particular, when comparing open-set to closed-set studies, comparing studies based on one-to-one vs. one-to-many examinations, and comparing studies that use notably different conclusion scales; see *SI Appendix*, *Appendix B* for a summary).

This study was conducted to provide data that can be used to assess the scientific validity of handwriting comparisons, for use by policy makers, laboratory managers, the legal community, and FDEs. This study follows the approach used in the previous FBI Laboratory–Noblis latent print black box study (11) and later recommended by the PCAST report. The design utilizes open-set, one-to-one document comparisons to evaluate the conclusions reached by practicing FDEs when comparing writing samples selected to be broadly comparable to casework. The primary purposes of the study are to measure the accuracy of conclusions by FDEs when comparing handwriting samples and to assess reliability by measuring the reproducibility (interexaminer variability) and repeatability (intraexaminer variability) of those conclusions. Secondary purposes include reporting any associations between the accuracy of the decisions in this study, factors related to the participants (such as training or experience), and factors related to the samples (such as quantity of writing, comparability of content, limitations, or style of writing).

## 1. Background

A forensic handwriting examination involves a side-by-side comparison of questioned and known writing samples for the purpose of determining whether the questioned writing was written by the writer of the known material. Questioned writing is a body of handwriting for which the writer is unknown. Known writing is a set of writing samples that are known to have been prepared by one specific individual. The forensic comparison of handwriting and the conclusions made are based on the training, education, and experience of the FDEs. There is, currently, no generally accepted quantitative model that can be used as the basis for source conclusion decisions by the FDEs.

Ideally, a handwriting comparison involves questioned writing that is original, is freely and naturally prepared, and contains a sufficient quantity and quality of writing. Questioned writing that is limited in one or more of these attributes may provide insufficient evidence to make a source determination. A handwriting comparison should also, ideally, include known writing that is original and freely and naturally prepared; is comparable to the questioned writing in format, style, characters, and character combinations; and is provided in sufficient quantity and quality to be representative of the individual’s range of writing. Limitations in the questioned or known writing may result in inconclusive or qualified conclusions.

A forensic handwriting comparison involves greater complexity than many other pattern-based forensic disciplines. Since handwriting is a learned neuromuscular activity (12), the FDE has to account for wider intrasubject variation than for disciplines based on physical characteristics (such as fingerprints) or manufactured items (such as firearms or footwear). Handwriting is not static and can be affected by a number of factors, including the writer’s graphic maturity; the environment in which one is writing; the substrate; the writing instrument; the emotional, physical, and/or mental state; medication; and more. Writers can also consciously decide to change the style (cursive, hand printing, mixed), size, and/or slant of the handwriting. Furthermore, handwriting is content dependent: The words, letter combinations, and numerals are prepared using an individual’s established subconscious and habitual writing pattern to prepare either a signature, a sentence, a paragraph, or a multipage document. Despite this variation, mature writers generally exhibit a clear and distinct pattern of characteristics that can be used to distinguish handwriting between individuals (e.g., refs. 13 and 14).

The predominant method for forensic handwriting examinations is known as analysis, comparison, evaluation, and verification (ACE-V). The analysis phase involves an independent evaluation (using proper lighting and magnification) of each questioned and known writing sample to assess whether the writings are original (for physical samples) and freely and naturally prepared and have sufficient quantity and quality of characteristics to be deemed suitable for comparison. Next, the side-by-side comparison involves assessment of comparability between the questioned and known writing, documentation of class and individual characteristics in the questioned writing, and the determination of whether or not those characteristics are also evident in the known writing samples. The evaluation phase follows, at which time the forensic examiner assesses the quantity and weight of similarities, differences, and limitations, to decide what conclusion is warranted. Finally, the examiner's conclusion is verified by another competent examiner. This method is discussed in more detail in various forensic document working group publications (15–17).

## 2. Materials and Methods

The full study protocol was approved by the FBI’s Institutional Review Board (IRB) for Human Subjects Research. This section summarizes the participants, study design, and the handwriting samples; see *SI Appendix*, *Appendix A* for further details.

### 2.1. Participation.

Participation was open to practicing examiners who had conducted operational casework within the previous 2 y, making conclusions using a conclusion scale of at least five levels. A total of 86 US and international handwriting examiners participated in this study, with US participation principally from nonprivate federal and state entities. Of the 86 participants, 64 completed at least 90 of the assigned comparisons.

Participation was solicited at relevant conferences and via professional organization announcements, email communications, and direct requests to laboratory management. No participants who met the requirements were barred from participation. Participants were required to complete an IRB-approved informed-consent form and background questionnaire prior to starting the study. The questionnaire responses were used to assess performance relative to examiner variables such as training, experience, and certification, as well as to inform an understanding of the participating examiners’ operational procedures, casework profiles, and affiliated agencies (e.g., agency function, accreditation, adopted conclusion scale).

Participants represented a broad cross-section of the FDE community. Of the 86 participants, 63% were employed by US federal, state, or local agencies, 27% were employed by international government agencies, 34% worked at private practices or companies, and 8% were academics (overlapping categories sum to over 100%); 41% conduct handwriting examination casework daily (26% a few times a week); 41% had testified in a legal setting at least 20 times (including depositions); and 73% had formal training of 2 y or more (not self-trained, not testifying during training). Participants were highly experienced: 50% had at least 16 y of experience. See *SI Appendix*, *Appendix C1* for all participant background survey responses.

### 2.2. Study Overview.

Each participant was asked to perform 100 handwriting comparisons over a period of about 10 mo. The test was administered using custom web-based software that presented examiners with 300-ppi digital images of each writing sample in the comparison set and collected all responses. Note that this study focuses on conclusions by individual FDEs, and therefore does not include verification of examiner conclusions, which is part of the ACE-V methodology.

For each comparison set, participants selected one conclusion from the following five-level scale:

• The questioned sample was written by the known writer (*Written*)
• The questioned sample was probably written by the known writer (*ProbWritten*)
• No conclusion (*NoConc*)
• The questioned sample was probably not written by the known writer (*ProbNot*)
• The questioned sample was not written by the known writer (*NotWritten*)

For some analyses we combine these responses, referring to “definitive” conclusions (*Written* and *NotWritten*) and “qualified” conclusions (*ProbWritten* and *ProbNot*). In casework, these conclusions are considered “qualified” because they must be accompanied by a clear statement that they are not definitive, and limitations must be documented in reports and communicated in testimony.

In this study, a five-level conclusion scale was selected as a common denominator familiar to most FDEs due to its widespread use in proficiency testing. A variety of conclusion scales are used throughout the document examination community. In the background survey conducted as part of this study, 53% of participants reported that they use a nine-level conclusion scale defined in the Scientific Working Group for Forensic Document Examination (SWGDOC) 2013 standard (18), 24% use a variation of that scale with at least seven levels, and 20% use a five- or six-level scale. Although the use of a five-level scale compresses the finer distinctions made on scales with more levels, it is more practical for the analysis and comparison of examiner performance. The instructions specified how other conclusion scales should be mapped to the five-level scale used here. There is much discussion in the forensic, statistical, and academic communities regarding the potential use of likelihood ratios (LRs) in reporting instead of categorical conclusions (see discussion specific to forensic document examination in ref. 17)—however, LRs are used only to a limited extent by the study population. Out of the 86 participants in the study, 76 (88%) do not use LRs or other probability measures in reporting conclusions, 9 (10%) use LRs or other probability measures in addition to (or in support of) the conclusion scale, and only one participant uses LRs or other probability measures instead of a conclusion scale. This is not limited to US examiners: 19 of the 26 non-US participants do not use LRs.

In addition to the conclusion, for each comparison, participants provided responses about sample limitations, difficulty of the comparisons, variation present in the writing, skill of the writer(s), style of writing, the two most influential attributes, and the basis of exclusion, if applicable. Participants had the option to comment on each comparison. For further detail, see *SI Appendix*, *Appendix D* for the instructions provided to participants.

### 2.3. Handwriting Samples.

Each comparison set (QKset) included one questioned sample (Q) and up to five samples from one known writer (K). Each participant was assigned 100 QKsets: 44 mated QKsets (in which the questioned and known samples were from the same writer) and 56 nonmated QKsets (in which the questioned and known samples were from different writers). The mating and ratio of mated to nonmated sets was not known to the participants. The participants could receive no more than 10 QKsets at a time for examination. The number of mated and nonmated sets within any given packet of 10 QKsets varied. To assess intraexaminer repeatability, each participant was assigned 10 QKsets that contained repeated imagery from an earlier comparison (i.e., each participant was assigned 100 total QKsets, but only 90 distinct QKsets). These repeated sets were assigned different QKset numbers and were separated from the initial comparisons by a median of 70 QKsets, to minimize the chances of participants recognizing the samples.

Most questioned handwriting samples (80%) were approximately half of a page in length; the remainder were generally one-sixth of a page in length, except each participant received two QKsets in which the Q was limited to an address. Known samples ranged from one to five samples of writing from a single writer (approximately half a page of writing each). Content was either free text or directed material such as specific addresses, the modified London letter, or the the Center of Excellence for Document Analysis and Recognition Forensic Examination (CEDAR-FOX) letter (see *SI Appendix*, *Appendix A3.2* for content). Approximately half of the QKsets were designed to contain the same text between the questioned and the known writing samples. The study design controlled the samples assigned to each participant in order to assess the effects of the quantity and the comparability of writing. Cursive, handprinted, and mixed styles of handwriting were all represented. This study did not include signature comparisons. To the best of our knowledge, the handwriting samples were prepared freely and naturally, and no instruction was given to writers to purposely distort their handwriting samples; this was stated explicitly in the instructions to participants. All results reported in this study are the result of digital image comparisons. Although an optional physical subtest was offered, it was abandoned due to lack of sufficient participation.

A total of 180 distinct QKsets (78 mated and 102 nonmated) were created from 230 distinct writers, selected from a pool of four sources containing more than 4,500 total writers. The handwriting samples were selected to span a range of attributes and difficulty found in casework: The study was designed to have each participant receive a distribution of samples that, collectively, could be considered to be similar to those encountered in casework. The relative difficulty of any forensic test is driven, in part, by the methods used to select nonmated subjects. In this study, we selected nonmated subjects that would appear to be similar when assessed by a layperson (“close nonmates”). This study does not include comparisons of arbitrary subjects with dissimilar styles of writing that could easily be distinguished, although some casework may include such easy comparisons. Subjects for nonmated QKsets were selected using a three-step process. First, writing samples were categorized into groups of potentially similar writers utilizing layperson evaluations. In parallel, all pairs of writers within and between multiple datasets were compared using the Forensic Language Independent Analysis System for Handwriting Identification (FLASH ID, Sciometrics LLC) automated handwriting identification system: Highest-scoring nonmates were selected for follow-on manual review. Next, layperson members of the study team reviewed these resulting groups and selected proposed pairs of “close nonmates.” Finally, the FDEs on the study design team used these proposed pairs to select the final sets of nonmated QKsets; nonmated subjects the study team FDEs considered “easy” comparisons were not included in the study, so the resulting dataset focused on moderate and difficult nonmated comparisons. Some individual writing samples in nonmated QKsets were cropped to remove grossly different characteristics. (See *SI Appendix*, *Appendix A3.3* for additional details.)

Ten of the nonmated QKsets were from 10 pairs of twins. Past studies have investigated whether the handwriting of twin writers can be distinguished (14) and whether monozygotic (identical) or dizygotic (fraternal) twin writing is more/less similar (19). Boot (20), Beacom (21), and Gamble (22) each noted that, although the handwriting of some twins demonstrates a “marked degree of similarity,” none of them wrote exactly alike, and their writing patterns were distinguishable.

## 3. Results

Analyses were based on a total of 7,196 responses from 86 participants on 180 distinct QKsets. To evaluate repeatability (intraexaminer variability), each participant who completed the study was assigned 10 QKsets twice: The responses on second assignments were not included in most analyses because a subset of the QKsets would have a disproportionate effect on the results, and because the second responses are not statistically independent. For this reason, second responses were not used to calculate overall conclusion rates.

For most analyses, we use the baseline dataset (6,576 responses from 86 participants on 180 distinct QKsets), which omits the 620 second assignments. For analyses of repeatability, we use the repeatability dataset, which includes 620 pairs of first and second assignments (1,240 total responses) by 65 participants on 20 distinct QKsets. For the analyses that computed and compared rates for individual participants, we use the examiner comparison dataset, a subset of the baseline dataset that is limited to those participants who completed at least 50 QKsets each (6,096 responses from 70 participants on 180 distinct QKsets) (see *SI Appendix*, *Appendix E* for details on the datasets and test yield).

Each of the 180 QKsets was assigned to one-half of the participants. The baseline dataset includes responses from 31 to 48 participants per QKset (mean 36.5, median 37)—overall (including repeats), 31 to 81 responses were received per QKset (mean 40.0, median 37).

### 3.1. Accuracy and Errors.

**Fig. 1.**
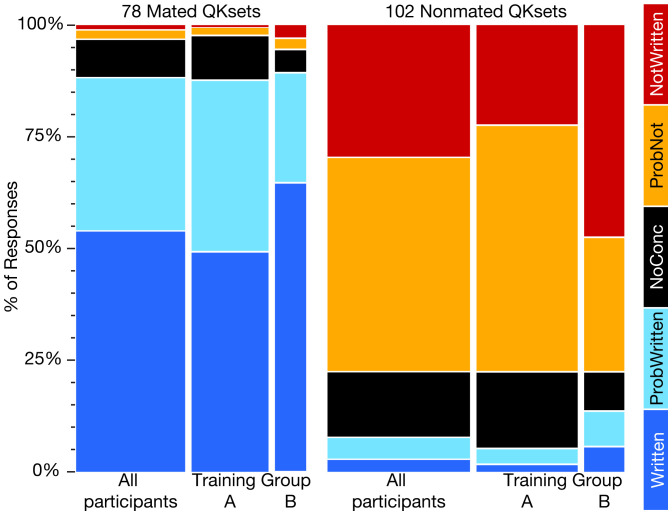
Distribution of conclusions. Training group results are discussed in section 3.5 (baseline dataset: All, 2,863 mated and 3,713 nonmated responses from 86 participants; group A, 2,026 mated and 2,635 nonmated responses from 63 participants; group B, 837 mated and 1,078 nonmated responses from 23 participants).

Accuracy refers to the extent to which participants’ conclusions agree with known source attribution (ground truth). Accuracy cannot be described using a single number but can be measured using a variety of metrics (detailed in *SI Appendix*, *Appendix F*). Fig. 1 illustrates the distribution of the participants’ conclusions in the baseline dataset, with the rates of each type of conclusion.

The use of a five-level conclusion scale requires consideration of how to address the results of qualified conclusions (*ProbWritten* and *ProbNot*). The proportion of trials on mated QKsets resulting in *Written* conclusions (true positive rate, TPR_PRES_) was 54.0%, but an additional 34.3% of trials resulted in *ProbWritten* conclusions (correct association rate, CAR_PRES_), so a total of 88.3% of trials on mated QKsets were consistent with ground truth.* The proportion of trials on nonmated QKsets resulting in *NotWritten* conclusions (true negative rate, TNR_PRES_) was 29.6%, and an additional 47.8% of trials resulted in *ProbNot* conclusions (correct nonassocation rate, CNR_PRES_), for a total of 77.4% of trials on nonmated QKsets that were consistent with ground truth.

Throughout this paper, we will use the term “error” to refer to definitive conclusions that contradict ground truth and refer to nondefinitive qualified conclusions that are not consistent with ground truth as “incorrect.” The reason for this distinction is that, in practice, participants explicitly differentiate between definitive and qualified conclusions, which is intended to convey different strengths of conclusions and/or different perceived weights of evidence, and, therefore, it would be inappropriate to lump them together into a single category of error. On nonmated QKsets, 3.1% of trials resulted in erroneous *Written* conclusions (false positive rate, FPR_PRES_), and an additional 4.8% of trials resulted in incorrect *ProbWritten* conclusions (incorrect association rate, IAR_PRES_). Thus, 7.9% of the conclusions in the nonmated trials contradict ground truth. On mated QKsets, 1.1% of trials resulted in erroneous *NotWritten* conclusions (false negative rate, FNR_PRES_), and an additional 2.1% of trials resulted in incorrect *ProbNot* conclusions (incorrect nonassocation rate, INR_PRES_). Consequently, 3.2% of the conclusions in the mated trials contradict ground truth. *NoConc* responses are regarded as neither correct nor incorrect, since they cannot be assessed in terms of ground truth: The proportion of trials resulting in *NoConc* conclusions on mated QKsets was 8.5% and, for nonmated QKsets, was 14.7%. See *SI Appendix*, *Appendix F2* for details regarding accuracy and error rates, and CIs.

Note that the FNRs are lower than the FPRs, and that the TNRs are lower than the TPRs; this is unlike other pattern fields [e.g., latent prints (11)], in which exclusion is generally easier (and more frequent) than identification. These results reflect the difficulty in eliminating writers, especially with a limited set of known writing samples, resulting in a smaller proportion of *NotWritten* conclusions. Further discussion of the challenges associated with eliminating writers is provided in *SI Appendix*, *Appendix F5*.

An alternative method of describing accuracy is through the use of conditional probabilities, which assess the likelihood that a given conclusion is correct. Overall, 93.1% of *Written* responses were correct (positive predictive value [PPV]), as were 97.2% of *NotWritten* responses (negative predictive value [NPV]), 84.6% of *ProbWritten* responses, and 96.7% of *ProbNot* responses. These rates are notably affected by the proportions of mated vs. nonmated data; see *SI Appendix*, *Appendix F3* for details and projections. The rates of accuracy and error were not evenly distributed by QKset or by participant, as is discussed further below.

### 3.2. Sample-Specific Effects.

**Fig. 2.**
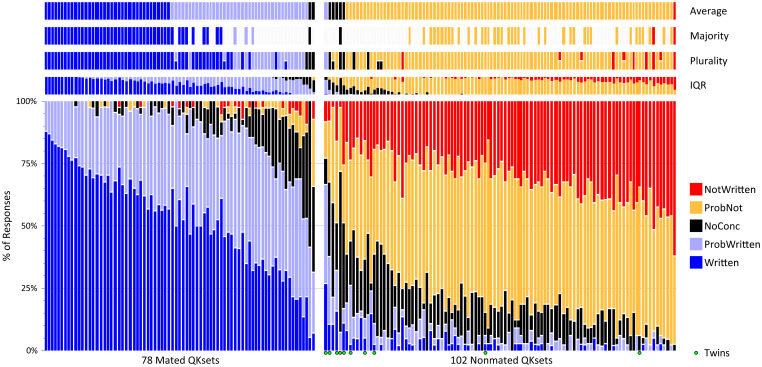
Decision rates by QKset, sorted by average conclusion. Multiple methods of assessing consensus are shown: average response, majority, plurality, and IQR. Green circles indicate nonmated QKsets in which the writers were twins. Averages were calculated by converting conclusions to an ordinal (1 to 5) scale and rounding to the nearest value. Each QKset received responses from 31 to 48 participants (mean 36.5) (baseline dataset: *n* = 6,576 responses).

Fig. 2 shows the same data as Fig. 1, but illustrates the distribution of conclusions by QKset, as well as multiple methods of assessing consensus for each QKset. Here we see that the distribution of conclusions is strongly affected by mating status and by specific QKsets.

In terms of conclusions consistent with ground truth, on the 78 mated QKsets, the proportion of *Written* conclusions (TPR_PRES_) ranges from 6 to 88%, and the combined proportion of *Written* and *ProbWritten* conclusions ranges from 32 to 100% (15 QKsets achieved 100%). On the 102 nonmated QKsets, the proportion of *NotWritten* conclusions (TNR_PRES_) ranges from 3 to 62%, and the combined proportion of *NotWritten* and *ProbNot* conclusions ranges from 23 to 98%. Note that over half of the mated QKsets (45/78) result in a majority definitive *Written* conclusion, but only two of the nonmated QKsets result in a majority definitive *NotWritten* conclusion.

In terms of conclusions contrary to ground truth, 22 of the 78 mated QKsets have at least one *NotWritten* conclusion in the baseline dataset (42 have at least one *NotWritten* or *ProbNot* conclusion). The proportion of erroneous *NotWritten* conclusions (FNR_PRES_) ranges up to 9%, and the combined proportion of erroneous/incorrect *NotWritten* and *ProbNot* conclusions ranges up to 34%. For nonmates, 55 of the 102 nonmated QKsets have at least one *Written* conclusion in the baseline dataset (89 have at least one *Written* or *ProbWritten* conclusion). The proportion of erroneous *Written* conclusions (FPR_PRES_) ranges up to 27%, and the combined proportion of erroneous/incorrect *Written* and *ProbWritten* conclusions ranges up to 67%.

*NoConc* responses represent a relatively small percentage of the responses: Only 6 mated and 13 nonmated QKsets received more than 25% *NoConc* responses. It is the majority response for two QKsets, and is the consensus response in terms of average or plurality for 11 QKsets.

**Fig. 3.**
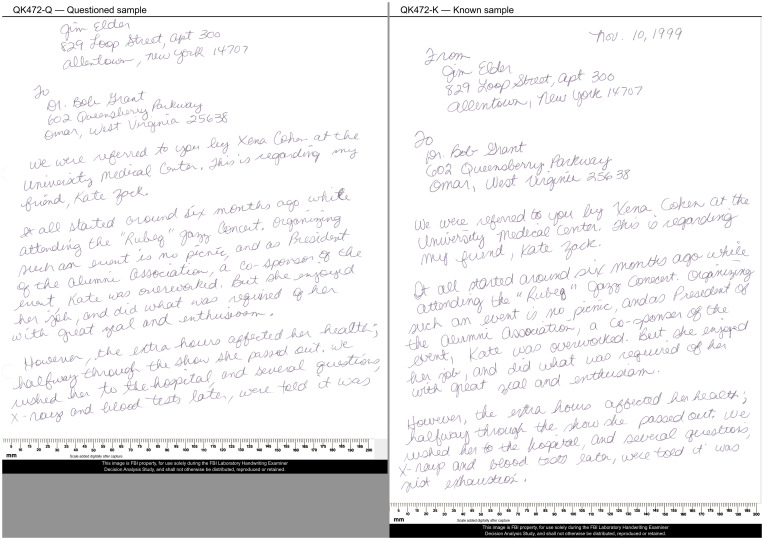
QK472: nonmated QKset that resulted in a total of 23 erroneous *Written* conclusions (13 in the baseline dataset, and an additional 10 in the repeats [responses from second assignments in the repeatability dataset], 5 of which were repeated errors). The subjects who wrote the Q and K were twins. The Q was cropped to eliminate the date and “From,” making discrimination more difficult. Conclusion rates for this QKset: *Written*: 13 in baseline data (10 additional in repeats); *ProbWritten*: 19 in baseline data (10 additional in repeats); *NoConc*: 5 in baseline data (7 additional in repeats); *ProbNot*: 7 in baseline data (4 additional in repeats); *NotWritten*: 4 in baseline data (2 additional in repeats).

The 10 QKsets that included twins as nonmated subjects had disproportionately high error rates (shown as green circles on the *x* axis in Fig. 2, *Bottom Right*). The FPR_PRES_ on the 10 QKsets from twins was 8.7%, compared to just 2.5% on the 92 nontwin nonmated QKsets. Of the 114 erroneous *Written* conclusions in the baseline dataset, 28% (32/114) were on nonmated QKsets collected from twins; 11% (13/114) occurred on the nonmated twins QKset that is shown in Fig. 3 (twins QKsets also resulted in a high rate of repeated errors, discussed in the repeatability analyses below).

The distribution of responses is strongly associated with the perceived difficulty of the comparison. Each participant assessed the difficulty of each comparison on a scale of [very difficult, difficult, moderate, easy, obvious/very easy]. Easier comparisons were associated with higher rates of definitive conclusions and lower rates of inconclusives: For example, 88% of “obvious/very easy” comparisons were definitive conclusions, and none were inconclusive; this contrasts with the “very difficult” comparisons, in which 10% were definitive and 51% were inconclusive. Assessments of easy comparisons are not proof against error: Of the 23 erroneous *Written* conclusions on QK472 (Fig. 3, including second assignments), 12 were “easy” and 7 were “very easy”; none were “difficult” or “very difficult.” Overall, a majority of erroneous *Written* conclusions were assessed as “easy” or “obvious/very easy.” In the posttest survey (*SI Appendix*, *Appendix C2*), participants assessed how the overall difficulty of the comparisons in this study corresponded to their casework: 12% indicated “easier,” 80% indicated “similar,” and 8% indicated “harder.” These assessments of overall difficulty may be debatable given that some participants made incorrect responses on comparisons they considered easy. However, if we ignore participants for whom 5% or more of responses were erroneous or incorrect yet assessed as easy, very easy, or moderate, the overall assessments are not notably different: 12% indicated “easier,” 82% indicated “similar,” and 6% indicated “harder.” Note that the results by difficulty provide insight into how data selection relates to the distribution of conclusions: If this study had included a greater proportion of easy comparisons, the conclusion distributions would be expected to shift to be more definitive, whereas, if it had included a greater proportion of difficult comparisons, the conclusion distributions would be expected to shift to be more inconclusive. See *SI Appendix*, *Appendix M* for additional details regarding participants’ assessments of difficulty.

### 3.3. Consensus and “Appropriate” Conclusions.

In this study, a given response can be evaluated in terms of whether it is consistent with or contradicts ground truth, but there are no such absolute criteria to judge whether a definitive or qualified conclusion is the appropriate response for a given comparison. FDEs use their expertise to determine whether a given comparison contains sufficient information to make a definitive conclusion, as opposed to a qualified conclusion or no conclusion. In making a comparison, FDEs assess a variety of factors in determining whether the samples are sufficient to make a given conclusion (clarity and detail of samples, availability of sufficient individual characteristics, range/variability of writing, writer skill, length of questioned and known writing, comparability of questioned and known writing, similarity/dissimilarity of features, limitations, and/or presence of characteristics observed in the questioned writing that could not be accounted for in the known writing provided). However, there are no precise criteria or thresholds that clearly delineate what constitutes sufficient information to make a specific conclusion for a given comparison. In the absence of specific criteria, we evaluate, in Fig. 2, the votes among FDEs as to what they collectively consider the appropriate conclusions. The proportions of responses aggregated across participants can be regarded as votes in a decision space: The proportion of *Written* vs. *NotWritten* responses (and, to a lesser degree, *ProbWritten* vs. *ProbNot*) provides a method to measure the relative amount of corresponding vs. contradictory information assessed by participants for each QKset.

Fig. 2 also illustrates that there are multiple methods of assessing the extent to which there is a consensus among FDEs on the “appropriate” conclusion for each QKset. Because the conclusion scale includes five categories, there is not always a single conclusion that received a majority of responses for a given QKset: 63% of the mated QKsets and 41% of the nonmated QKsets had a majority consensus. In contrast to majority, the plurality and average conclusions can be determined for every QKset (in some cases, two conclusions tied for the plurality of responses). The consensus conclusions obtained using plurality and average were in alignment on 85% of all QKsets; when plurality and average consensus conclusions differed, it was always by a single conclusion category, and the conclusions determined using average were generally more conservative. Interquartile range (IQR) uses a different approach to assess consensus: Rather than a specific conclusion, this approach includes the range of conclusions that fall within the 25th to 75th percentiles; this discounts the top quarter and bottom quarter of responses and treats the center half of responses as the consensus (24). The consensus conclusions for mated and nonmated data differed in regard to definitive vs. qualified conclusions: On nonmated data, the consensus across all four methods was overwhelmingly for the qualified conclusion (*ProbNot*), whereas, on mated data, there is a range of consensus from definitive to qualified, and the four methods all show a consensus for the definitive conclusion (*Written*) on nearly half of the mated QKsets. This shows an asymmetry in *Written* vs. *NotWritten* conclusions: There was often a consensus among participants for *Written* conclusions, but rarely for *NotWritten* conclusions. This result indicates the relative difficulty of excluding vs. identifying a writer in this study as the source of a sample; see *SI Appendix*, *Appendix F5* for additional discussion.

Majority consensus may arguably serve as a reasonable mechanism for determining the most appropriate conclusion (sometimes described as the “forensically correct” response) for a given comparison. For QKsets that do not have a majority, the plurality, average, and/or IQR may be a suitable proxy. Note, however, that the distinctions between these approaches can be made here because over 30 FDEs provided responses for each QKset. In casework, determining the appropriate response will necessarily be based on the conclusions of a few FDEs: There is unlikely to be a difference between majority, plurality, and average if only three FDEs review a comparison, and IQR cannot be defined for fewer than five responses.

A consensus response is not necessarily proof against error. Majority conclusions were never erroneous or incorrect, but the majority was often undefined. Plurality and average conclusions were never erroneous and were incorrect on only a single nonmated QKset, which resulted in a consensus conclusion of *ProbWritten* using both metrics (QK472; Fig. 3). The IQR includes an erroneous response for one (mated) QKset, and incorrect responses for several QKsets.

### 3.4. Comparing Participants.

Rates of accuracy and error were not evenly distributed among participants: in the baseline dataset, nearly half of the participants (42/86) reported at least one erroneous *Written* conclusion, but a majority of the errors were made by just eight participants (61/114). Erroneous *NotWritten* conclusions were made by 17 of the participants, but one participant made seven such errors, thus accounting for 22% of the total (7/32).

**Fig. 4.**
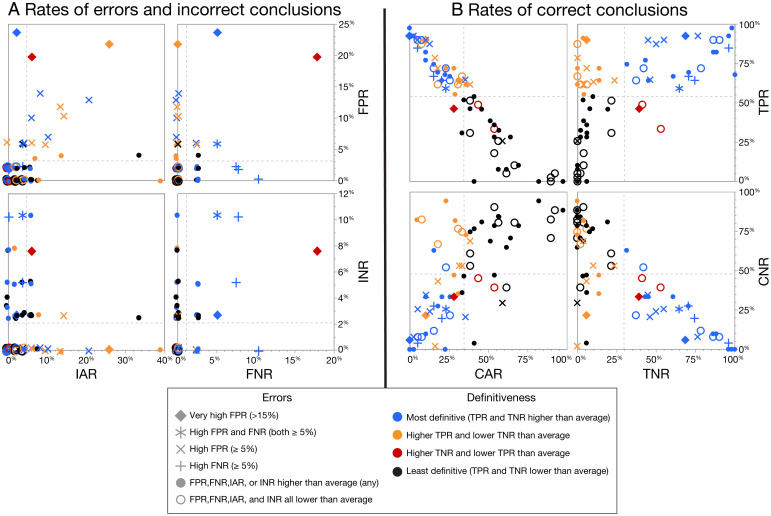
Comparison of participants by (*A*) rates of errors and incorrect conclusions and (*B*) rates of correct conclusions. Means are shown as dashed lines (examiner comparison dataset: *n* = 70 participants who completed at least 50 total comparisons; rates are calculated from 6,096 responses; markers in *A* are jittered to minimize superimpositions; rates are calculated based on mean of 37.9 mated and 49.2 nonmated QKsets per participant).

Because of the use of a five-level conclusion scale, FDE performance cannot be assessed using a single measure: Performance is multidimensional and must be based on rates of errors and incorrect conclusions (FPR, FNR, IAR, and INR) and rates of correct conclusions (TPR, TNR, CAR, and CNR) but must also consider the relative proportions of definitive vs. qualified conclusions and indeterminate responses. Fig. 4 compares the participants in multiple dimensions. Fig. 4*A* shows the rates of conclusions contrary to ground truth, with error rates (FPRs and FNRs) in the top right quadrant; the interactions with the incorrect qualified conclusion rates (IARs and INRs) are shown in the other quadrants. Fig. 4*B* shows the corresponding rates of conclusions consistent with ground truth, with TRPs and TNRs in the top right quadrant, and interactions with the CARs and CNRs in the other quadrants. When calculating rates for each participant, analyses are based on the examiner comparison dataset, which is limited to the 70 participants who completed at least 50 QKsets. The symbols and colors correspond between Fig. 4*A* and Fig. 4*B* (see *SI Appendix*, *Appendix J* for tables and additional figures).

For example, in the top right quadrant of Fig. 4*A*, the participant with the highest rate of erroneous *Written* conclusions had an FPR of 24% and FNR of 5% (blue diamond); following that shape to the other quadrants, we see both IAR and INR for that participant were 2%. In Fig. 4*B*, we see that participant had notably high TPR (92%) and TNR (69%), while CAR and CNR were well below average. In other words, although that participant had high rates of correct conclusions, that came at the cost of errors: This participant made 12 erroneous *Written* conclusions in the baseline dataset (3 of which were repeated, for a total of 15, as further discussed in the repeatability analyses below).

The interrelations of these rates are instructive when reviewing the three participants with the highest FPRs. The participant shown with a blue diamond rarely made qualified conclusions and could be seen as overly decisive. The participant shown with a red diamond made both definitive and qualified conclusions but was disproportionately incorrect on all types of conclusions. The participant shown with a gold diamond made many *Written* conclusions (both correct and erroneous), was wrong on most *ProbWritten* conclusions (13/17 *ProbWritten* were on nonmates), but made few *NotWritten* or *ProbNot* conclusions; this participant could be seen as aggressive on *Written* and *ProbWritten* conclusions but cautious on *NotWritten* and *ProbNot* conclusions.

There is not a notable association between erroneous *Written* and erroneous *NotWritten* conclusions (Fig. 4*A*, *Top Right Quadrant*): Of the eight participants with the highest FPRs, five had a 0% FNR. There is an association between erroneous *Written* and incorrect *ProbWritten* conclusions (Fig. 4*A*, *Top Left Quadrant*): Most participants who had an FPR above average also had an above-average IAR; interestingly, two of the participants with the highest FPR (blue and red diamonds) had low IARs, and the participant with the highest IAR had a 0% FPR. There is not a notable association between erroneous *NotWritten* and incorrect *ProbNot* conclusions (Fig. 4*A*, *Bottom Right Quadrant*): Most of the participants with an above-average INR had a 0% FNR. There is not a notable association between incorrect *ProbWritten* and incorrect *ProbNot* conclusions (Fig. 4*A*, *Bottom Left Quadrant*).

Fig. 4*B* shows rates of conclusions consistent with ground truth. In the top right quadrant of Fig. 4*B*, we see that TNR and TPR were generally associated, but some participants (shown in gold) who often made *Written* conclusions made few *NotWritten* conclusions. Participants with high TNR and high TPR often had high error rates. There are only five blue open circles, indicating participants who were above average on TNR and TPR and below average on all error rates. One participant (Fig. 4*B*, *Top Right Quadrant*, blue dot, TNR = 98%, TPR = 97%) made only definitive conclusions, resulting in one false positive and one false negative, and all other responses were either true positives or true negatives.

*NoConc* responses are considered neither correct nor incorrect, as they are neutral with respect to ground truth. Participants who disproportionately make *NoConc* responses would avoid errors (low FPR, FNR, IAR, and INR), but would also avoid making correct conclusions (low TPR, TNR, CAR, and CNR), and would thereby be ineffective. Eight participants made *NoConc* responses on over 25% of trials, two of whom made *NoConc* responses on the majority of trials (55% and 71%).

Some participants avoided specific conclusions. Out of the 70 participants in the examiner comparison dataset, only 45 used every type of conclusion. Three participants never made any definitive conclusions (*Written* or *NotWritten*), and an additional 12 never made any *NotWritten* conclusions. One participant only made definitive conclusions (i.e., never made any *ProbWritten*, *ProbNot*, or *NoConc* responses). Nine participants never made *NoConc* responses. These tendencies would affect rates of accuracy and consensus. For example, the participants who never made definitive conclusions would necessarily have TPR, TNR, FPR, and FNR all equal to 0%. Consensus among examiners is necessarily limited by this—in effect, over a third of participants (25/70) did not use the full conclusion scale. See *SI Appendix*, *Appendix J2.1* for details.

### 3.5. Associating Participant Performance with Background.

The accuracy of participants was notably associated with participants’ training. As part of the background survey (*SI Appendix*, *Appendix C1*), participants were asked a number of questions about their training. Using their responses, participants were grouped based on the 2013 SWGDOC recommendations for minimum training requirements (25) and the 2018 American National Standards Institute National Accreditation Board (ANAB) guiding principles regarding competency and proficiency (26):

• Training group A comprised 63 participants who received two or more years of training under the supervision of a principal trainer and did not testify during their training;
• Training group B comprised 23 participants who received less than 2 y of formal training or noted they were self-trained, and/or those who testified as an expert in handwriting examinations prior to completing their handwriting training.

Although 69 participants indicated that they had two or more years of formal training, 3 of the 69 said that they were “self-trained” [contrary to SWGDOC guidelines (25)], and an additional 3 said that they testified as an expert during training [contrary to ANAB principles (26)]; see *SI Appendix*, *Appendix K1* for discussion.

Fig. 1 shows a notable association between the training groups and conclusion rates. In general, participants in training group A were less likely to make definitive conclusions than training group B: Training group A responses were 35% definitive, 51% qualified, and 14% no conclusion; training group B responses were 60% definitive, 33% qualified, and 7% no conclusion. As a result, training group B showed significantly higher rates of both erroneous (FPR, FNR) and correct (TPR, TNR) conclusions. In contrast, the participants in training group A appeared to be more cautious, rendering more qualified and inconclusive decisions (*ProbWritten*, *ProbNot*, and *NoConc*). Accordingly, the training group A participants had lower rates of both erroneous (FPR, FNR) and correct (TPR, TNR) conclusions, but the decisions they made were more likely to be correct, resulting in higher predictive values than their training group B counterparts (PPV: 95.2% vs. 89.6; NPV: 99.0% vs. 95.2%). Differences between the training groups were significant [compared using a χ^2^ analysis with Bonferroni-adjusted Pearson residual post hoc tests (27); see *SI Appendix*, *Appendix K1* for details]. The five participants with the highest FPRs were in training group B, as were the six participants with the highest FNRs (for a total of nine participants: Two participants overlapped). The trend seen in training group B in which a larger proportion of conclusions were definitive, resulting in more correct answers but higher error rates, was also seen in Kam et al.’s (5) 1997 study, which compared FDE and layperson performance. In that study, laypeople were found to “overmatch,” providing both a high TPR and high FPR as well. While participants in this study are not laypeople, a statistically significant difference in error rates is apparent based on the type of training received.

To further characterize the performance of FDEs in this study, we utilized a multiphasic approach for detecting and reporting any associations between participants’ background and performance. In order to assess performance in light of correctness, definitiveness, and the relative value/cost of making definitive vs. qualified conclusions, we developed four weighted performance ratios (detailed in *SI Appendix*, *Appendix J1*). These combine the rate for a definitive conclusion (e.g., FPR) and the rate for the corresponding qualified conclusion (e.g., IAR) by weighting the qualified conclusion as half the definitive conclusion (e.g., “weighted FP–IA ratio” is based on FPR+IAR/2). Given there is no absolute means of assessing the relative values of definitive vs. qualified conclusions, this makes the simple assumption that costs and benefits of qualified conclusions are half those of definitive conclusions: For example, this assumes that the benefit of a correct *ProbWritten* conclusion is half the benefit of a correct *Written* conclusion, and the cost of an incorrect *ProbWritten* conclusion is half the cost of an erroneous *Written* conclusion.

These four weighted measures of performance were evaluated with respect to 27 background attributes using two complementary approaches: variable importance analysis (VIA) and attribute-specific significance testing. VIA considers all attributes simultaneously and was executed by coupling linear regression and random forest analysis to yield importance scores. On the other hand, significance testing was conducted for each attribute individually using the Kruskal–Wallis test to yield *P* values and *q* statistics [see *SI Appendix*, *Appendix K2* and (34) for additional details]. Using these importance scores, *P* values, and *q* statistics, association thresholds were set and used to develop a hierarchy of reporting criteria to determine which (if any) of the background attributes exhibited sufficient support to indicate an association with any of the weighted performance ratios. Because these analyses are based on conclusion rates calculated for each participant, we limit these analyses to the 70 participants who completed at least 50 comparisons (examiner comparison dataset); the remaining 16 participants completed as few as 20 comparisons each, which would be inadequate for reliable estimates of individual rates.

Based upon this analysis, we did not detect support for an association with performance for 18 of the 27 background attributes considered, including education, experience, and examination frequency. However, two attributes exhibited strong support for an association with weighted FP-IA ratios: training group (as discussed above) and training provider. Participants in training group A (two or more years of formal training) had significantly lower weighted FP–IA ratios (i.e., reported fewer *Written* and/or *ProbWritten* on nonmated pairs) than group B. Similarly, with regard to training provider, participants who did not have formal training and/or were self-trained had significantly higher weighted FP–IA ratios than participants trained by US state agencies, and marginally higher ratios than participants trained by US federal and US local agencies. Note that training program and training provider are highly correlated attributes, with a bias-corrected Cramer’s V (28, 29) of 0.60. For further details and discussion of seven additional attributes that exhibited limited support for association with performance, see *SI Appendix*, *Appendix K2*.

### 3.6. Repeatability and Reproducibility.

The reliability of FDE conclusions can be assessed in terms of repeatability (intraexaminer consistency) and reproducibility (interexaminer consistency): Repeatability refers to the extent to which a participant provides the same conclusion when given the same QKset on different occasions, whereas reproducibility refers to the extent to which different participants agree on conclusions when given the same QKset. The lack of repeatability and reproducibility can be described as system noise, which Kahneman et al. (30) define as “unwanted variability in judgements that should ideally be identical.” The lack of repeatability (also known as occasion noise) affects the reliability of the comparison process: It may be a point of concern if examiners provide notably different conclusions on exactly the same evidence on different occasions.

In this study, each participant who completed the study was assigned 10 QKsets on two different occasions (separated by a median of 70 QKsets). These repeats resulted in 620 pairs of conclusions by 65 participants on 20 distinct QKsets (repeatability dataset). Overall, 68% of conclusions were repeated exactly, and 92% were repeated within ±1 conclusion category; only 1% of conclusions were diametrically opposed (i.e., *Written* vs. *NotWritten*). These results suggest that the majority of the occasion noise lies within participants’ inconsistent assessments of the strength of their conclusions rather than differences in their interpretations of source. Conclusions consistent with ground truth (*Written* and *ProbWritten* on mated data; *NotWritten* and *ProbNot* on nonmated data) were repeated at least 73% of the time. Participants generally did not repeat their own *NoConc* responses, suggesting that these assessments are less stable than the other conclusions. Of the 47 participants who each completed 10 repeated QKsets, only one participant repeated every conclusion exactly.

Most errors and incorrect responses were not repeated. On mated QKsets, no erroneous *NotWritten* conclusions were repeated, but one incorrect *ProbNot* was repeated, and one *ProbNot* was changed to *NotWritten* on the second assignment. On nonmated QKsets, 20 erroneous *Written* conclusions (FPs) were made on the first assignments of repeated QKsets, of which 8 (40%) were repeated on the second assignments, and another 3 (15%) were changed to *ProbWritten*. Overall, there were a total of 121 FPs on 55 distinct QKsets: 84 on assignments that were not repeated and, within the repeatability dataset, 20 FPs on first assignments and 17 on second assignments; there were 8 FPs repeated (i.e., on both first and second assignments). Three of the repeated FPs were made by the participant with the highest rate of erroneous *Written* conclusions (shown as a blue diamond in Fig. 4). That participant made 12 FPs on first assignments, 3 of which were represented as second assignments, and all 3 of those FPs were repeated, for a total of 15 FPs by this individual. Repeated FPs were disproportionately associated with the twins data. Out of the eight repeated FPs, seven were made on QKsets from twins (three distinct QKsets). The 10 nonmated QKsets from twins resulted in a total of 38 FPs on 8 QKsets: 11 on assignments that were not repeated and, within the repeatability dataset, 14 FPs on first assignments and 13 on second assignments; there were 7 FPs repeated (i.e., on both first and second assignments). The QKset shown in Fig. 3 resulted in a total of 23 erroneous *Written* conclusions by 18 distinct participants: 13 on first assignments (*Baseline Dataset*) and an additional 10 on second assignments. See *SI Appendix*, *Appendix H* for more detailed repeatability results.

We evaluated the reproducibility of responses based on all pair-wise combinations of responses from different participants on the same QKsets (236,366 pairwise combinations of responses derived from the 6,576 responses in the baseline dataset). For example, for every participant who responded *Written* on a mated QKset, 60% of the other participants also responded *Written*, 33% responded *ProbWritten*, and 5% responded *NoConc*. The reproducibility of responses is much lower than the repeatability of responses. One may expect that knowing an expert’s conclusion would be a good basis to predict the conclusions of other experts, but the only instance in which a majority of participants reproduced conclusions exactly was for *Written* conclusions on mated data. Overall, 40.4% of conclusions are reproduced exactly (46.4% mated; 35.8% nonmated), and 84.5% are reproduced within ±1 conclusion. However, only 1.2% of conclusions resulted in contradictory conclusions, with one examiner reporting *Written* and a second examiner reporting *NotWritten* (0.9% on mated data; 1.5% on nonmated data). Similar to the occasion noise observed for repeated responses, much of the variability between different examiners’ responses can be attributed to disagreements regarding the strength of conclusions (within ±1 conclusion) rather than disagreements regarding interpretations of source. In the reproducibility results, the distributions of responses are remarkably similar regardless of conclusion type: Particularly for nonmated data, knowing one participant’s response is a very weak predictor of other participants’ responses. Repeatability and reproducibility are both associated with training groups, in that training group B has a greater proportion of conclusions that differ by three or four categories, which could be expected from the higher error rates and increased proportions of definitive conclusions. There is no notable effect on reproducibility if limited to the participants who reported all conclusions (e.g., omitting participants who never reported definitive conclusions). High levels of reproducibility are generally desirable, but are problematic if errors are reproduced. For erroneous *Written* conclusions, 7.9% were reproduced exactly, and an additional 11.7% resulted in the second examiner reporting *ProbWritten*. For erroneous *NotWritten* conclusions, 2.2% were reproduced exactly, and an additional 5.7% resulted in the second examiner reporting *ProbNot*. See *SI Appendix*, *Appendix I* for more detailed reproducibility results.

**Fig. 5.**
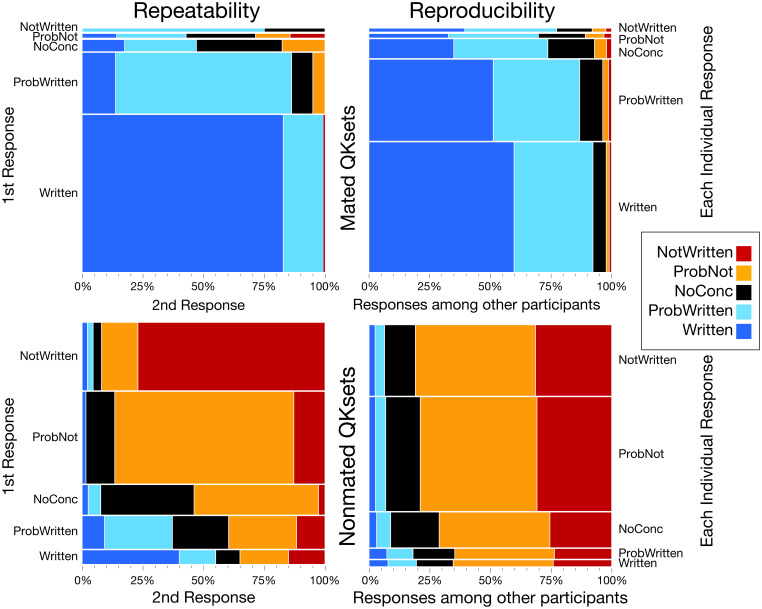
Repeatability and reproducibility of conclusions (repeatability dataset: 620 first responses [313 mated, 307 nonmated], 620 second responses [313 mated, 307 nonmated]; reproducibility: 236,366 pairwise combinations of responses [103,398 mated, 132,968 nonmated] derived from the 6,576 individual responses in the baseline dataset [2,863 mated, 3,713 nonmated]).

Fig. 5 summarizes the repeatability and reproducibility results. For example (in the bottom row of the mated repeatability chart in Fig. 5), if a participant responded *Written* on the first assignment of a mated QKset, on the second assignment of that QKset, that participant responded *Written* 83% of the time, or *ProbWritten* 17% of the time. In terms of reproducibility (in the bottom row of the mated reproducibility chart), for every participant who responded *Written* on a mated QKset, 60% of the other participants also responded *Written*, 33% responded *ProbWritten*, and 5% responded *NoConc*. In comparing the repeatability and reproducibility results, note that the reproducibility distributions of responses are remarkably similar regardless of conclusion type: Particularly for nonmated data, the categories are close to vertical rather than the diagonals seen in the repeatability results.

### 3.7. Effect of Quantity and Comparability of Writing.

To evaluate the effect of sample-related factors on accuracy, this study conducted a methodical analysis of the effects of three factors: quantity of questioned writing, quantity of known writing, and content comparability. The quantity of the questioned writing was divided into three categories: long (QL, approximately half a page or about 130 words), short (QS, approximately one sixth of a page or about 45 words), and address (QA). The quantity of known writing was limited to one, three, or five pages of known writing (labeled K1, K3, and K5). Content comparability was divided into same (S, at least one page of identical content between the Q and K, generally a London or CEDAR-FOX letter) and different (D, no standardized content in common). To conduct the analysis of the effects of quantity and comparability of writing, 32 pairs of QKsets were created, with each pair varying a single factor but otherwise keeping all factors consistent. For example, a factor analysis evaluating the effect of content comparability presented a short questioned sample and three pages of known writing with either the same or different content as the questioned writing (QS-K3-x). Two QKsets were created that contained the same questioned sample, but one QKset contained content in the known writing that was identical to the questioned (QS-K3-S), whereas the other QKset contained known writing content that was different from the questioned (QS-K3-D). Each participant was assigned one QKset from each of the 32 pairs, so that the effect of each factor could be assessed by comparing half of the participants with the other half.

Two of the factor evaluations exhibited significant effects: On mated comparisons, increasing the quantity of known writing or going from different to the same content resulted in a significant increase in correct conclusions. When participants were presented with more known samples (K5 vs. K1), there was a significant increase in correct *Written* or *ProbWritten* conclusions and a significant decrease in *NoConc* conclusions—on mated QKsets that contained a long questioned sample and different content between Q and K (QL-Kx-D). When participants were presented with the same vs. different content, there was a significant increase in correct *Written* conclusions, a significant decrease in *NoConc* conclusions, and a significant decrease in erroneous *NotWritten* and incorrect *ProbNot* conclusions—on mated QKsets that contained a short questioned sample and three pages of known writing (QS-K3-x). Both of these results show that more known samples and adequate comparability to the questioned writing samples increase the probability of accurate conclusions. In practice, FDEs can often request additional known writing samples (or request known samples that are directly comparable to the questioned samples), but, in this study, they were restricted to the samples provided. Overall, significant differences in accuracy were not detected for the majority of the factor pairings, but there were trends observed for a number of factors that were not statistically significant due (possibly) to the limited sample size.

When considering all QKsets included in the study (and not just restricted to the 32 pairs described above), the same general trends are observed, particularly with respect to content comparability. When the known writing contained the same content as the questioned writing, there was a notable increase in correct *Written* conclusions and a notable decrease in *NoConc* conclusions; this result persisted even when the questioned writing was extremely limited and contained only an address. This same effect was observed for nonmated comparisons, but only when the QKset contained extremely limited questioned writing: There was an increase in correct *NotWritten* conclusions and a decrease in *NoConc* conclusions for comparisons in which the questioned writing contained only addresses. For additional details and discussion regarding the effects of amount and comparability of writing, see *SI Appendix*, *Appendix G*.

### 3.8. Cursive vs. Printed Writing.

In the *United States v. Johnsted* case (31), a federal judge sought to understand whether there was a meaningful difference in the error rates of cursive versus hand printing examinations. Based on the evidence presented at that time, the judge noted “the limited testing that exists is inconclusive as to the reliability of hand printing analysis” and decided to exclude the testimony of the handwriting expert in that case (31). Therefore, we designed this study to probe whether there was a difference in performance of participants based on writing style. The style of writing of the Q in each QKset was assessed by each participant using a five-category scale ranging from disconnected printing to connected cursive (see *SI Appendix*, *Appendix D6* for details). Using a majority of participants to assess the writing style of the Q in each QKset, 37% were disconnected printing, 29% were connected cursive, and the remaining 34% were considered “mixed” (see *SI Appendix*, *Appendix L* for details). We did not observe any association between writing style and rates of errors or incorrect conclusions. We detected one significant difference in conclusion rates: On mated QKsets in which the content of the questioned and known writing were the same, the conclusions reported for cursive samples were significantly more conservative than those for mixed or print samples (i.e., fewer conclusions of *Written* and more determinations of *ProbWritten* or *NoConc*). Conclusion rates did not vary significantly as a function of writing style for nonmated trials, or on mated trials with different content; there was no difference in rates for mixed vs. print writing. For further details and discussion regarding conclusions by writing style, see *SI Appendix*, *Appendix L1*.

## 4. Conclusions

The rates measured in this study are intended to provide estimates to inform decision-making and guide future research. In order to maximize the relevance of the reported rates, the samples were selected to span a range of quality, quantity, and attributes found in casework. A supermajority of participants responded that the samples in the study (both questioned and known) were representative of casework, and that the overall perceived difficulty of the comparisons in this study corresponded to casework (*SI Appendix*, *Appendix C2*).

The participants represented a broad cross-section of the FDE community. We observed notable associations between training and performance: FDEs with less than 2 y of formal training generally had higher error rates, but they also had higher rates of correct conclusions (TPR and TNR) because they tended to give more definitive conclusions; FDEs with at least 2 y of formal training were less likely to make definitive conclusions, but, when made, those conclusions were more likely to be correct (higher PPV and NPV). The performance of participants varied widely in terms of rates of errors and of correct conclusions, as well as in proportions of definitive vs. qualified conclusions. Of the 86 participants, 42 made at least one erroneous *Written* conclusion in the baseline dataset, for an overall FPR of 3.1%; 17 participants made at least one erroneous *NotWritten* conclusion, for an overall FNR of 1.1%. Nonmated samples from twins were disproportionately associated with errors: The FPR for comparisons of twins’ handwriting samples was 8.7%, as opposed to 2.5% for samples from nontwins. The variation in rates among participants and among samples underscores the limitations of overall error rates in studies such as this: Although overall error rates are beneficial in providing baseline estimates to serve in the understanding of a discipline, such rates cannot be assumed to apply to any particular examiner or any particular sample.

Knowing one participant’s response is not a strong predictor of other participants’ responses: Participants reported exactly the same conclusion 40.4% of the time, but 84.5% of conclusions were reproduced within ±1 conclusion, suggesting that most of the variability between participants’ conclusions can be explained by differences in strengths of conclusions and generally does not indicate differences in assessments of authorship. Only 1.2% of the responses resulted in contradictory definitive conclusions (*Written* vs. *NotWritten*). The results suggest that blind verification by a second examiner would be expected to detect most—but not all—erroneous conclusions prior to reporting, given that 7.9% of erroneous *Written* conclusions and 2.2% of erroneous *NotWritten* conclusions were reproduced. As shown in previous studies (32, 33), a lack of reproducibility can be explained, in part, by a lack of repeatability: In this study, participants only repeated 68% of their own conclusions exactly, which limited the ability to reproduce the conclusions of other participants. The results regarding reproducibility and consensus on appropriate conclusions highlight the importance of having a quality assurance program that includes procedures such as verification and technical review; note that 88% of participants require technical review and/or verification in their casework. Limited reproducibility could be of particular concern in operational workflows in which a single examiner’s conclusion is reported without verification.

Note that these results may not be representative of all forensic document casework, do not account for operational quality assurance such as technical review and verification, did not allow for consultation with other subject matter experts as needed, and did not allow for requests for additional known writing from contributors. The examination procedure used may differ from that used by some FDEs, particularly regarding the conclusion scale and use of digital images.

This study was conducted for use by policy makers, laboratory managers, the legal community, FDEs, and the forensic science research community. These results may be used, in part, to assess the scientific validity of handwriting comparisons, to provide data for use in assessing changes in operational procedures, to enhance FDE training, and to enhance quality assurance procedures.

## Supplementary Material

Supplementary File

## Data Availability

The dataset of participants’ responses for all 7,196 trials, summary of responses by participant, and de-identified survey responses for the 86 participants is included in the supporting information for this article. Due to IRB requirements requiring confidentiality of participants, the survey data includes no identifiers, which could have been used to re-identify participants.

## Footnotes

^*^Throughout this paper, rates are calculated based on all presentations (“PRES”) of QKsets (11, 23); see *SI Appendix*, *Appendix F1* for discussion.
