# Supplementary material for: Accuracy and reliability of forensic handwriting comparisons
Source: Proc Natl Acad Sci U S A. 2022 Aug 1;119(32):e2119944119. doi: 10.1073/pnas.2119944119 (PMC9371688; doi:10.1073/pnas.2119944119)
Supplement: Supplementary File [file pnas.2119944119.sapp.pdf]

Accuracy and Reliability of Forensic Handwriting Comparisons

Supplemental Information — Appendices

Contents

Appendix A Materials and Methods 3
Appendix A1 Study description 3
Appendix A1.1 Comparison responses 3
Appendix A1.2 Schedule 4
Appendix A2 Participants 4
Appendix A2.1 Awards for participation 5
Appendix A3 Handwriting data 5
Appendix A3.1 Sources of handwriting data 5
Appendix A3.2 Sample content 7
Appendix A3.3 Selection of nonmated QKsets 8
Appendix A3.4 Selection of mated QKsets 12
Appendix A3.5 Quantity and content of questioned and known samples 12
Appendix A3.6 Assignments 12
Appendix A3.7 Image preparation 13
Appendix B Previous Work 14
Appendix C Survey Responses 15
Appendix C1 Participant background survey responses 15
Appendix C2 Participant post-test survey responses 26
Appendix D Study Instructions 27
Appendix D1 Overview 27
Appendix D1.1 Digital Subtest 27
Appendix D1.2 Physical Subtest 27
Appendix D2 Eligibility and Registration 27
Appendix D3 Participation Conditions 27
Appendix D4 Comparison Sets 27
Appendix D5 Handwriting Black Box Software (HBBS) Website 28
Appendix D6 Comparisons 29
Appendix D7 Submitting Responses for a Comparison Set 32
Appendix D8 Completion 32
Appendix D9 Frequently Asked Questions (FAQ) 32
Appendix E Test Yield 35
Appendix F Conclusion rates 37
Appendix F1 Defining Terms 37
Appendix F2 Accuracy and Error Rates 37
Appendix F3 Conditional probabilities 39
Appendix F4 False positives (FPs)—Erroneous "Written By" conclusions 40
Appendix F5 Challenges in eliminating writers ("Not Written By" conclusions) 41
Appendix F6 False negatives (FNs) —Erroneous "Not Written By" conclusions 42
Appendix F7 Repeatability of Errors and Incorrect Responses 43
Appendix G Effect of quantity and comparability of writing 45
Appendix G1 Analysis of pairs of factors assessing quantity and comparability 45
Appendix G1.1 Major takeaways – general 46
Appendix G1.2 Effect of quantity of questioned writing 46
Appendix G1.3 Effect of quantity of known writing 46
Appendix G1.4 Effect of same/different content 47
Appendix G2 Addresses 47
Appendix G3 Questioned and known writing amount and content comparability 47
Appendix H Repeatability of conclusions 50
Appendix I Reproducibility of conclusions 53
Appendix J Participant effects 57
Appendix J1 Comparing participants 57
Appendix J2 Conclusions by participant 59
Appendix J2.1 Avoidance of specific conclusions by participants 62
Appendix J2.2 Definitiveness among participants 62
Appendix K Associations with participant background 64

Appendix K1 Effect of 2 years of formal training....64
Appendix K2 Associating Participant Background Attributes with Performance....66
Appendix K2.1 Strong Support for Association with Performance.... 68
Appendix K2.2 Limited Support for Association with Performance.... 69
Appendix L Writing style.... 72
Appendix L1 Conclusions by writing style....72
Appendix M Effect of Perceived Difficulty.... 74

Supplemental Data:

- Handwriting Black Box Public Data: Spreadsheet containing participants' responses for all 7196 trials, summary of responses by participant, and de-identified survey responses for the 86 participants.*

** Due to IRB requirements requiring confidentiality of participants, the survey data includes no identifiers, which could have been used to re-identify participants. Note this means that this spreadsheet does not include data that could be used to associate participants' background information with their performance.*

Appendix A Materials and Methods

Appendix A1 Study description

This study was designed to assess the accuracy and reliability of forensic document examiner (FDE) conclusions. Each participant was asked to perform 100 handwriting comparisons, over a period of approximately ten months, using digital images. For each comparison set (QKset), participants were asked to compare one questioned item (Q) with up to five samples from one known writer (K). This study was conducted using custom web-based software that presented images and recorded participant responses.

The test comprised 100 handwriting comparisons. Participants were unaware of the mated and nonmated prevalence: each participant was assigned 44 mated and 56 nonmated QKsets. To assess intra-examiner repeatability, each participant was assigned ten QKsets (out of the 100 total) that contained repeated imagery from an earlier comparison. These repeated sets were assigned different QKset numbers and were separated from the initial comparisons by a median of 70 QKsets (average 66.6; range 18-93), to prevent participants from recognizing the samples. Upon consent to participate in the study, each examiner agreed to conduct the evaluations with the same diligence employed in their operational casework, not to collaborate on any examinations, and to discard/delete all study-related notes and images upon completion of each comparison set. Participants were limited to a maximum of ten comparisons per 24 hours, and could have up to ten unsubmitted comparisons open at a time.

The full study protocol was approved by the Federal Bureau of Investigation's Institutional Review Board for Human Subjects Research. Anonymity was maintained through multiple levels of anonymization, data segregation and information flow control. Participant anonymity was provided through the use of randomly assigned ParticipantID numbers. ParticipantIDs were anonymized prior to data analysis, precluding the analysis team's ability to cross associate participants, personally identifying information, questionnaire responses or test results. Destruction of existing cross-reference indices occurred prior to public presentation of results (e.g., indices correlating ParticipantIDs with packet delivery postal addresses). Therefore, participant identities could not be associated with the results at any point during analysis, or subsequently, such as for discovery. Participants were assumed to be volunteers. However, pressure to participate from employers or other entities cannot be precluded, nor the performance effect of such a factor calculated.

The following sections provide additional details on the study design and data selection.

Appendix A1.1 Comparison responses

For this study, participants were asked to use a five-level scale, with the instructions "Select the conclusion that best characterizes your determination, based on the comparison of the questioned and known samples." In parentheses we indicate the abbreviations used throughout this document:

- The questioned sample was written by the known writer (***Written***)
- The questioned sample was probably written by the known writer (***ProbWritten***)
- No conclusion (***NoConc***)
- The questioned sample was probably not written by the known writer (***ProbNot***)
- The questioned sample was not written by the known writer (***NotWritten***)

The conclusion scale used for this Handwriting Black Box (BB) Study is similar to the scale used in proficiency tests that are widely used across the FDE community. This scale was selected as it was expected to be familiar to a wide number of domestic and international examiners.

In addition to the comparison conclusion, participants provided additional responses for each QKset (Table S1).

Basis of exclusion	For *NotWritten* and *ProbNot*, indicates whether the decision was primarily a holistic decision, primarily based on individual characteristics, or a mix of both.
Limitations	Indicates whether the examination process was limited by clarity/detail of the questioned/known writing, quantity of the questioned/ known writing, insufficient individualizing characteristics, comparability of the writing, presence of unexplained characteristics, and/or distorted writing.
Difficulty	Assessment of the level of difficulty of the comparison.
Skill level	Assessment of the writer's skill level.
Type of writing	Indicates the type of writing in the questioned sample – printing, connected printing, mix of printing and cursive, disconnected cursive, or cursive.
Variation in the questioned sample	Assessment of the amount of variation in the questioned sample.
Variation in the known sample(s)	Assessment of the amount of variation in the known sample.
Comparability	Assessment of the comparability of writing content between the questioned and known samples.
Most influential features	Indicates the two most influential features for the comparison (only on samples that did not result in a decision of *NoConc*).
Need for original documents	Indicates whether the rendered conclusion would be stronger if provided with original documents.

Table S1. Responses collected from participants for each QKset comparison. See Appendix D for additional details.

Appendix A1.2 Schedule

Registration for this study remained open from March 2019 through March 2020, and test access was available from 1 August 2019 through 1 June 2020. Participants were asked to complete 100 handwriting comparisons over a period of approximately ten months. Participants were only allowed to view and submit responses to ten QKsets at a time, and this was the maximum number of submissions accepted in a 24-hour period; otherwise, participants were permitted to complete comparisons at their own pace and convenience.

Appendix A2 Participants

Participation was open to practicing forensic document examiners who had conducted operational casework within the two years preceding the study announcement. For the purposes of this study, a practicing examiner was defined as *an examiner who has performed handwriting evidence comparisons in operational casework within the last two years, and makes conclusions using a conclusion scale of at least 5 levels*.

Participation was solicited at relevant conferences, via email communications and direct requests to laboratory management, and via professional organization announcements:

- American Academy of Forensic Sciences (AAFS)
- American Board of Forensic Document Examiners (ABFDE)
- Association of Forensic Document Examiners (AFDE)
- American Society of Questioned Document Examiners (ASQDE)
- American Society of Crime Laboratory Directors (ASCLD)
- Australasian Society of Forensic Document Examiners (ASFDE)
- Board of Forensic Document Examiners (BFDE)
- Canadian Society of Forensic Science
- Council of Federal Forensic Laboratory Directors (CFFLD)
- European Network of Forensic Science Institutes (ENSFI) European Network of Forensic Handwriting Experts (ENFHEX)
- International Association of Document Examiners (IADE)
- Mid-Atlantic Association of Forensic Scientists (MAAFS)
- Midwestern Association of Forensic Scientists (MAFS)
- National Association of Document Examiners (NADE)
- NIST Organization of Scientific Area Committees (OSAC) Forensic Document Examination subcommittee
- Special Advisory Group on Document Examination (DocSAG)
- Southeastern Association of Forensic Document Examiners (SAFDE)
- Scientific Association of Forensic Examiners (SAFE)

- Southwestern Association of Forensic Document Examiners (SWAFDE)

No participants who met the requirements were barred from participation. Participants were required to complete an IRB-approved informed-consent form and background questionnaire prior to starting the study. The questionnaire responses were used to assess performance relative to examiner variables such as training, experience, and certification, as well as to inform an understanding of the participating examiners' operational procedures, casework profiles, and affiliated agencies (e.g., agency function, accreditation, adopted conclusion scale).

Although participation cannot be considered truly random, the pool of participants encompassed a diverse range of background and experience. Overall, the participant pool of US and international handwriting examiners reflected a broad range of education, employer type, training and experience levels. Questionnaire responses are profiled in *Appendix C1*.

Appendix A2.1 Awards for participation

To encourage participation, the study initially included four awards for randomly selected participants who completed all assigned comparisons: one $1,000 and three $500 awards. Participants were instructed that these prizes would only be awarded after all results were collected and that rapid completion of the test would not improve the chances of winning to decrease the likelihood of participants rushing. In addition to these four awards, participants were later offered the opportunity to be paid $5 per comparison as an additional incentive to encourage study completion (see announcement below).

> *Dear Handwriting Decision Analysis Study Participant,*
>
> *Registration for the study was closed earlier today, Monday March 16, 2020.*
>
> *We have over 100 participants, however, our overall completion rates are lower than we had hoped. After numerous internal discussions, we have decided to add another incentive: we will pay participants $5 for every comparison that is submitted by the study end date of May 17th, with a minimum of 20 required. This translates to $100 (for 20 comparisons), and up to $500 (if all 100 comparisons are completed). We will use your data during analyses if you submit at least 20 comparisons. This incentive is in addition to the raffle incentive for participants who complete all comparisons (one $1000 and three $500 prize).*
>
> *Please note that in registering for this study, you have already agreed to conduct the comparisons in this study with the same regard and diligence used when conducting handwriting evidence comparisons in operational casework, and not to conduct the comparisons in this study collaboratively.*
>
> *If you have any questions, please email handwriting@idealinnovations.com. We will compile the questions and disseminate answers to all participants in the FAQ (Frequently Asked Questions). We can anticipate some questions:*
>
> - *No data will be accepted after May 17th, at 11:59pm Eastern Daylight Time (23:59, UTC-4).*
> - *We closed registration prior to announcing these incentives: they are only available to participants who are already registered.*
> - *The incentives apply to comparisons already submitted, as well as any received by the end date.*
>
> *We thank you for your energy and endurance thus far — the results will provide important data to assess the scientific basis of handwriting examination, for use within the document examination community, by laboratory management, and the legal community.*

Appendix A3 Handwriting data

The handwriting samples were selected to span a range of attributes and difficulty found in casework: the study was designed to have each participant receive a distribution of samples that collectively could be considered to be similar to those encountered in casework, to maximize the relevance of the results.

This study leveraged handwriting data that was both collected for previous studies as well as specifically requested from donors for purposes of this study. These data originated from four sources and contained both transcribed and free-form text. The following sections offer additional details regarding the sample collection, sample content, and selection of QKsets for inclusion in this study.

Appendix A3.1 Sources of handwriting data

The handwriting samples used in the study comprised 230 distinct subjects, selected from a total of 4601 subjects from four sources, as described in Table S2 and the following sections.

Dataset	*Total subjects*	*Subjects used in study*
NARA	2797	24
Requested	394	47
Twins	410	34
WVU	1000	125
Total	4601	230

Table S2. Sources of handwriting data, including the total number of subjects in the dataset and the number of subjects retained for use in this study.

A3.1a West Virginia University (WVU) dataset

The "WVU" dataset was collected by West Virginia University (WVU) at locations in West Virginia under a cooperative agreement with and with technical direction by the FBI in 2011. Participation was solicited by WVU through web and local announcements; participation was open to all—no affiliation with WVU was required. Each participant was asked to provide three samples of the modified London letter (see Appendix A3.2a) and one freeform writing sample containing at least 2,500 characters (typically 3 pages or more) in their natural handwriting. The participants were given printed text containing the modified London Letter from which to copy. For the freeform sample, participants were allowed to write anything conceived in their minds, but they were provided prompts—such as a letter to a friend or a childhood memory—that they could use to focus their attention toward writing a coherent sample.

Participants were given a 30-minute pizza and soda break between writing the modified London Letter samples and the freeform sample, and were incentivized with a small gift card ($50) for completing all of the samples. Perhaps due to the incentives and the fact that three different sample collection events were held, instances of duplicate participants were detected across events. Suspected duplicates could be detected and removed by comparing the demographic survey information provided by the participant prior to each session. In such cases of detected duplication, the demographic information provided was either identical or extremely similar. However, if repeat participants provided notably different questionnaire responses they might remain undetected.

The collection of this data set was originally designed in accordance with recommendations from Saunders et. al. (1) for follow-on statistical studies. There were 6,290 total samples of handwriting collected from 1,000 subjects. The content of these samples included 2,784 modified "London letters" and 3,506 samples with free text: each subject usually had three modified London Letters and at least three pages of freeform text. Writing was in black ink on plain paper and was scanned in grayscale at 300 ppi (pixels per inch).

A3.1b National Archives (NARA) dataset

The "NARA" dataset was received from the National Archives and Records Administration (NARA) and scanned for a previous study. This collection is a set of handwritten letters from citizens of the United States containing their opinions on the government using Electronic Fund Transfers to deposit checks directly to a bank (aka direct deposit). Samples ranged from one to thirteen pages per subject. The NARA dataset was from public records available in 1976, containing 2,797 subjects. The content is free text with the National Commission on Electronic Fund Transfer address included. Writing was in a variety of colors of ink (some pencil), on a variety of types of paper. Images were scanned at 300 ppi (24-bit color).

A3.1c Twins dataset

It has long been established in the forensic handwriting community that the handwriting of individuals who are taught to write in the same school system may share similar characteristics. Similarities are most likely to be seen between class characteristics of the specific copybook style (e.g., Zaner-Blosner, Palmer, D'Nealian, etc.) taught in the school system and influenced by the same teachers. Twins, or individuals of multiple births, are increasingly likely to share some handwriting characteristics, as they are usually brought up in the same school system, influenced by the same individuals, and exhibit high genetic similarity. Past studies have investigated whether the handwriting of twin writers can be distinguished (2) and if monozygotic (identical) or dizygotic (paternal) twin writing is more/less similar (3). Boot (4), Beacom (5) and Gamble (6) each noted that while the handwriting of some twins demonstrates a "marked degree of similarity", none of them wrote exactly alike and their writing patterns were distinguishable. Nevertheless, these marked similarities can present challenges to FDEs when only

a small amount of both questioned and known writing is provided for examination and no context is given as to any relationship between writers.

Accordingly, this study opted to include writing samples from twins in order to evaluate performance of FDEs when presented such samples. The "Twins" dataset used in this study was received from the United States Secret Service (USSS). The images were collected at a Twins Day Festival in Twinsburg, Ohio in 2003. The twins came from 30 different states and five foreign countries. This data contains one iteration of a one-page letter, known as "the CEDAR-FOX" letter (see Appendix A3.2b) from 205 pairs of twins (410 subjects). The CEDAR-FOX Letter includes two handwritten addresses. This dataset is the same dataset as used in Shrihari's study testing the discriminability of twin writing sets (2). Writing is in blue or black ink on plain paper, scanned at 300ppi (24-bit color).

Note that since each subject had a single CEDAR-FOX letter, this prevented creating mated QKsets from the Twins dataset. (One mated QKset was created for one of the subjects from the Twins dataset, but the Q was limited to an address.)

Note that while the examination of the handwriting of twins is interesting to study, the low percentage of twins in the overall population indicates that scenario in casework would be relatively infrequent. A recent study reports that monozygotic twin rates are 4 per 1000 deliveries and dizygotic twin rates are 12 per 1000 deliveries worldwide, with varying rates by country (7).

A3.1d Requested dataset

Sample handwriting packets were requested from various sources to obtain original handwriting to be used in this study. Participation to provide handwriting was voluntary and the packets were distributed at professional meetings, federal and state government law enforcement agencies, and government contractors, among others. The sample packets to collect handwriting were distributed to volunteers willing to participate along with a demographic questionnaire. These sample handwriting packets returned original handwriting that was comparable in wording to the NARA addresses, as well as the modified London letter, CEDAR-FOX letters, and free text. Writing was in black or blue ballpoint pen, on plain (occasionally lined) paper. Samples from the Requested dataset were scanned at 600 ppi (24-bit color), but downsampled to 300 ppi for consistency with the other sources.

Appendix A3.2 Sample content

Sample content datasets were selected from four sources: the London Letter, the CEDAR-FOX Letter, the four mailing addresses that were part of the NARA dataset, and free text.

A3.2a London Letter

The "London letter" has been used for many years as a standard handwriting exemplar because it contains each numeric digit, at least one instance of every uppercase letter, and relatively representative proportions of each lowercase letter (1). Note that some (early) versions of the London letter end at "…tonight." More recent versions include the last two sentences ("My daughter chastised…"), which were added to include specific letter combinations (1).

> *Our London business is good, but Vienna and Berlin are quiet. Mr. D. Lloyd has gone to Switzerland and I hope for good news. He will be there for a week at 1496 Zermott Street and then goes to Turin and Rome and will join Colonel Parry and arrive at Athens, Greece, November 27th or December 2nd. Letters there should be addressed 3580 King James Blvd. We expect Charles E. Fuller Tuesday. Dr. L. McQuaid and Robert Unger, Esq., left on the "Y. X. Express" tonight. My daughter chastised me because I didn't choose a reception hall within walking distance from the church. I quelled my daughter's concerns and explained to her that it was just a five-minute cab ride & it would only cost $6.84 for this zone.*

A3.2b CEDAR-FOX Letter

The "CEDAR-FOX letter" was developed in 1999 by the Center of Excellence for Document Analysis and Recognition (CEDAR) at the University at Buffalo, State University of New York, for NIJ project 1999-IJ-CX-K010 (8). For the Requested dataset, the addresses were collected separately from the body of the letter to provide more options when compiling QKsets.

Nov 10, 1999

From
Jim Elder
829 Loop Street, Apt 300
Allentown, New York 14707

To
Dr. Bob Grant
602 Queensberry Parkway
Omar, West Virginia 25638

We were referred to you by Xena Cohen at the University Medical Center. This is regarding my friend, Kate Zack.

It all started around six months ago while attending the "Rubeq" Jazz Concert. Organizing such an event is no picnic, and as President of the Alumni Association, a co-sponsor of the event, Kate was overworked. But she enjoyed her job, and did what was required of her with great zeal and enthusiasm.

However, the extra hours affected her health; halfway through the show she passed out. We rushed her to the hospital, and several questions, x-rays and blood tests later, were told it was just exhaustion.

Kate's been in very bad health since. Could you kindly take a look at the results and give us your opinion?

Thank you!
Jim

A3.2c NARA Mailing Addresses

This address was included in most samples from the NARA dataset. These two variations (complete and abbreviated) were included in the Requested dataset.

National Commission On Electronic Fund Transfers<br>Suite 900<br>100 Connecticut Ave NW<br>Washington DC 20036	*Nat'l Comm. On EFTS<br>Suite 900<br>100 Connecticut Ave NW<br>Washington DC 20036*

A3.2d CEDAR-FOX Mailing Addresses

The CEDAR-FOX letter includes these two addresses. These addresses were included separately from the body of the CEDAR-FOX letter in the Requested dataset to provide more options when compiling QKsets.

Jim Elder<br>829 Loop Street, Apt 300<br>Allentown, New York 14707	*Dr. Bob Grant<br>602 Queensberry Parkway<br>Omar, West Virginia 25638*

A3.2e Other addresses

Some of the samples in the Requested dataset included the following addresses.

Senator Z. Augustus<br>Truman Office Building<br>Washington, D.C. 21212	*The Portificarial Corporation<br>84 Fifth Avenue, Suite 1379<br>New York, NY. 12742*	*Bob & Jenny Gleberheim<br>1752 Brookhome Way #24G<br>Minneapolis, Minnesota 46287*

Appendix A3.3 Selection of nonmated QKsets

The relative difficulty of any forensic test is driven in part by the methods used to select nonmated subjects. Randomly selecting subjects for comparison out of a very large population will overwhelmingly result in subjects with unrelated styles of writing that can easily be excluded even by a layperson. One challenge in designing a test such as this is in determining how nonmates are selected, as well as how the data selection applies to operational casework:

- In the latent print black box study (9), nonmates could be selected based on searches of the FBI's national automated fingerprint identification system (AFIS), which at the time had a database of approximately 58 million persons with criminal records, or 580 million distinct fingers. This approach both provided a means to select nonmates for the study and provided a baseline for the applicability of the study to operational casework: comparisons in that study were selected to reflect the types of comparisons that would be encountered in AFIS searches.
- For forensic tests of manufactured items (when large database searches are not available), nonmated samples can be selected based on make, model, or other manufacturing class attributes. For example, in a footwear test, nonmated Qs and Ks can be selected based on the model and size of the shoe; in a firearms test, nonmated Qs and Ks can be selected based on the make, model, and caliber of both the firearm and ammunition. Comparisons in such tests could then state the baseline for applicability to casework would be comparisons with identical manufacturing class characteristics.

For handwriting, neither approach is readily available. There is no large-scale equivalent to an AFIS in general use. Although handwriting has class characteristics, they are not clearly and distinctly defined as in manufactured items.

In this study, the baseline we used was selecting subjects that appeared to be similar to a layperson ("Close nonmates").

Nonmated QKsets were selected using a three-step process:

1. We used laypersons (on the study team and via Amazon Turk) to categorize samples, resulting in groups of potentially similar writers.
   - In parallel, the handwriting comparison system FLASH ID® was used to select potentially similar writers.
   - All pairs of twins from the Twins dataset were considered potentially similar writers.
2. Layperson members of the study team reviewed potentially similar writers and selected pairs of "close nonmates."
3. The FDEs on the study design team selected the final set of nonmated samples included in the study from the larger pool of close nonmates provided by the laypersons: FDEs assessed these as "easy", "moderate", or "difficult", and only the nonmated samples assessed as "moderate" or "difficult" were included in the study. In general, the close nonmates selected by the FDEs were chosen to present samples that would be challenging for another expert to distinguish. Relatively few were rated as "difficult" by the FDEs: of the 102 nonmated QKsets used in the study, 91 were assessed as "moderate" and 11 were assessed as "difficult" by the FDEs on the study design team.

These assessments were conducted on all the writing samples from an individual, while the QKsets provided to participants of the study were generally a subset of the writing available from that individual. In creating the nonmated QKsets, the specific samples were selected to avoid grossly different characteristics, and some individual writing sample images in the nonmated QKsets were manually cropped after the FDE review to remove grossly different characteristics.

A3.3a Categorization using Amazon Turk

To avoid comparing all subjects to each other (cross-comparing four thousand subjects would have involved eight million comparisons), we categorized the subjects using the rubric shown in Table S3. This rubric was implemented in Amazon Turk and used for categorizing the Twins, NARA, and WVU datasets. Amazon Turk is a crowdsourcing marketplace that makes it easier for individuals and businesses to outsource their processes and jobs to a distributed workforce who can perform these tasks virtually on a piecework basis. This rubric was used to sort the samples into roughly similar, binned categories. Multiple Amazon Turk ratings of each sample were used to make the process more fault tolerant and reduce the probability of poor-quality or nonconsensus responses.

Is this on lined paper?	• Yes<br>• No
Writing style:	• Cursive<br>• Mixed cursive and printed<br>• Printed
Connectedness:	• Always connected: all letters in each word are always connected to each other<br>• Usually connected: the letters in each word are usually connected to each other<br>• Usually disconnected: the letters in each word are usually separated from each other<br>• Always disconnected: all letters in each word are separated from each other
Capitalization:	• All upper case (ALL CAPS)<br>• Mostly upper case<br>• Some words are all upper case<br>• Normal use of upper and lower case<br>• Mostly lower case<br>• Always lower case
Skill:	• Highly skilled and artistic writing<br>• Normal skill in writing<br>• Unskilled
Uniformity and Consistency:	• Very consistent: letters and spacing are very uniform<br>• Usually consistent: letters and spacing are usually uniform<br>• Usually inconsistent: letters and spacing often differ<br>• Very inconsistent: letters and spacing differ extensively
Legibility:	• Good legibility (much more than usual)<br>• Normal legibility<br>• Poor legibility (much worse than usual)
Slant:	• Very consistent slant: letters are always in the same direction<br>• Consistent slant: letters are usually in the same direction<br>• Inconsistent slant: letters are often in different directions<br>• Very inconsistent slant: letters are angled in lots of different directions
Direction of slant (optional: only if slant is consistent or very consistent):	• Extreme left slant<br>• Left slant<br>• Upright<br>• Right slant<br>• Extreme right slant

Table S3. Handwriting categorization rubric.

The results of the categorization rubric were distilled into the bins shown in Table S4:

- Type (combining the Writing style, Connectedness, and Capitalization questions from Table S3)
- Skill (combining the Skill, Uniformity and Consistency, and Legibility questions)
- Slant (combining the Slant and Direction of Slant questions)

For our purposes, this was adequate in reducing the overall population into bins with a relatively manageable number of samples for cross comparison. However, we found that the delineation between categories was often inconsistent, so the resulting classifications could not be considered rigorous — therefore, for the Requested dataset (which was completed late in the process), we bypassed the Amazon Turk process and staff on the study team classified the samples.

Type	Skill	Slant	Total samples
Cursive	HighSkill	SlantLeft	27
Cursive	HighSkill	SlantRight	352
Cursive	HighSkill	SlantUpright	150
Cursive	LowSkill	SlantInconst	36
Cursive	LowSkill	SlantLeft	8
Cursive	LowSkill	SlantRight	77
Cursive	LowSkill	SlantUpright	50
Cursive	NormalSkill	SlantInconst	149
Cursive	NormalSkill	SlantLeft	185
Cursive	NormalSkill	SlantRight	1163
Cursive	NormalSkill	SlantUpright	809
Mixed-Connected	HighSkill	SlantLeft	1
Mixed-Connected	HighSkill	SlantRight	15
Mixed-Connected	HighSkill	SlantUpright	22
Mixed-Connected	LowSkill	SlantInconst	16
Mixed-Connected	LowSkill	SlantLeft	1
Mixed-Connected	LowSkill	SlantRight	5
Mixed-Connected	LowSkill	SlantUpright	8
Mixed-Connected	NormalSkill	SlantInconst	43
Mixed-Connected	NormalSkill	SlantLeft	76
Mixed-Connected	NormalSkill	SlantRight	82
Mixed-Connected	NormalSkill	SlantUpright	213
Mixed-Disconnected	HighSkill	SlantLeft	3
Mixed-Disconnected	HighSkill	SlantRight	2
Mixed-Disconnected	HighSkill	SlantUpright	8
Mixed-Disconnected	LowSkill	SlantInconst	21
Mixed-Disconnected	LowSkill	SlantLeft	5
Mixed-Disconnected	LowSkill	SlantRight	2
Mixed-Disconnected	LowSkill	SlantUpright	15
Mixed-Disconnected	NormalSkill	SlantInconst	68
Mixed-Disconnected	NormalSkill	SlantLeft	50
Mixed-Disconnected	NormalSkill	SlantRight	26
Mixed-Disconnected	NormalSkill	SlantUpright	146
Print	HighSkill	SlantLeft	4
Print	HighSkill	SlantRight	5
Print	HighSkill	SlantUpright	36
Print	LowSkill	SlantInconst	5
Print	LowSkill	SlantLeft	3
Print	LowSkill	SlantRight	2
Print	LowSkill	SlantUpright	11
Print	NormalSkill	SlantInconst	30
Print	NormalSkill	SlantLeft	35
Print	NormalSkill	SlantRight	30
Print	NormalSkill	SlantUpright	179
Print-AllCaps	HighSkill	SlantRight	4
Print-AllCaps	HighSkill	SlantUpright	2
Print-AllCaps	NormalSkill	SlantInconst	2
Print-AllCaps	NormalSkill	SlantRight	9
Print-AllCaps	NormalSkill	SlantUpright	16

Table S4. Bins used in grouping subjects for preselection of potentially similar writers. (4207 subjects from the WVU, Twins, and NARA datasets)

A3.3b Selection of similar subjects using FLASH ID®

The Forensic Language Independent Analysis System for Handwriting Identification (FLASH ID®, Sciometrics LLC, Chantilly, VA) is a handwriting derived biometric analysis software used to quantify features of handwriting. This software evaluates spatial and geometric aspects of the graphemes in the handwriting and compares it to a database of writing samples, ultimately providing a ranked similarity score for writers in that database. Using these similarity scores, FLASH ID® enables detection of pairs of similar writers. Although the similarity scores returned by FLASH ID® were of value in selecting potentially similar subjects, these scores are sample-specific and are not scalable, so the resulting values are not appropriate as measures of similarity across samples.

In this study, all pairs of writers within and between multiple datasets were compared (as appropriate) using FLASH ID®, and the highest scoring nonmates were selected for follow-on manual review.

Appendix A3.4 Selection of mated QKsets

In general, no special selection process was used to select mated QKsets. In order to include a range of mated comparisons, a small number of subjects were selected for higher-than-usual variability (flagged by layperson review, Amazon Turk rating inconsistency, and low FLASH ID® hit rate), resulting in five mated QKsets flagged as "high-variability mates;" an additional 15 mated QKsets were flagged as debatably high-variability mates, with the remaining 58 subjects randomly selected.

Appendix A3.5 Quantity and content of questioned and known samples

The quantity and content of the questioned and known writing used for testing were all selected from natural writing. There was no attempt to deceive the study participants with handwriting that was known to be distorted or disguised.

- Q length — The quantity of the questioned writing was divided into three categories: address (QA), short (QS), and long (QL). A short "questioned" document would contain the equivalent to ⅓ of a London letter. The long "questioned" document would contain the equivalent of a London letter.
- K length — The quantity of known writing was limited to one, three or five pages of known writing samples (labelled K1, K3, K5).
- Same vs different content — A QKset was considered the same content if the exact content of the Q was included at least once in the K(s).

Appendix A3.6 Assignments

Each participant who completed the study was assigned 90 distinct QKsets as shown in Table S5. A total of 180 QKsets were developed, and each participant was assigned half of these. Each participant received relatively balanced quantities of QKsets in terms of K length (K1, K3, K5) and same-different content (SD). Most assignments were of long Q (QL), with smaller quantities of short Q (QS) and addresses (QA) to assess their effects. The only assignments that differed between participants were the addresses (QA): each participant received either two QA-K1-D or two QA-K1-S.

Q amount	K amount	Same-Diff	Total	Mated	Nonmated
QA	K1	[D or S]	2	1	1
QL	K1	D	12	4	8
QL	K1	S	14	6	8
QL	K3	D	12	5	7
QL	K3	S	12	5	7
QL	K5	D	11	5	6
QL	K5	S	11	5	6
QS	K1	D	2	1	1
QS	K1	S	2	1	1
QS	K3	D	4	2	2
QS	K3	S	4	2	2
QS	K5	D	2	1	1
QS	K5	S	2	1	1
QA			2	1	1
QS			16	8	8
QL			72	30	42
	K1		32	13	19
	K3		32	14	18
	K5		26	12	14
		D	43	18	25
		S	45	20	25
		D or S	2	1	1

Table S5. Assignments of distinct QKsets for each participant.

Each participant who completed the study was re-assigned ten QKsets to assess repeatability of conclusions, as shown in Table S6. These were limited to the same content to limit the likelihood of remembering repeated samples (same content was generally London Letters or CEDAR-FOX letters; different content was generally free text, which would presumably be memorable based on content).

Q amount	K amount	Same-Diff	Total	Mated	Nonmated
QL	K1	S	6	3	3
QL	K3	S	4	2	2

Table S6. Assignments of repeated QKsets for each participant.

Note that we explicitly chose not to assign all QKsets to all participants. By assigning each QKset to half of the participants, we collected results from a larger number of samples, limiting the impact of sample-specific effects. Each QKset in the *Baseline Dataset* resulted in responses from 31-48 participants (mean 36.5, median 37), which is more than sufficient to allow analyses of consensus and reproducibility; if we assigned all QKsets to all participants we would not improve the measurability of those factors, but would only have had data on 90 QKsets. Because handwriting has so many complexities, if we had used fewer QKsets, we would have been less comfortable about representativeness, and more concerned that a single unusual QKset would have undue impact.

Appendix A3.7 Image preparation

The following steps were followed in preparing images:

- Histogram equalization was used to guard against participants using image attributes in making their comparisons.
- Samples were reviewed for reverse text (on the back of paper); if present it was removed, except in cases in which the front and back of the same page were both included as knowns.
- QS and QA samples were cropped to the defined length.
- Images were cropped close to the text, and preprinted or extraneous text was cropped or redacted.
- After subjects were selected for use in a QKset, a layperson selected among the samples available for those subjects; for nonmated QKsets, samples were selected to avoid writing that appeared obviously different from a layperson's view.
- Some samples were cropped if a layman assessed some characteristics were unusually distinctive (as shown in Fig S1).

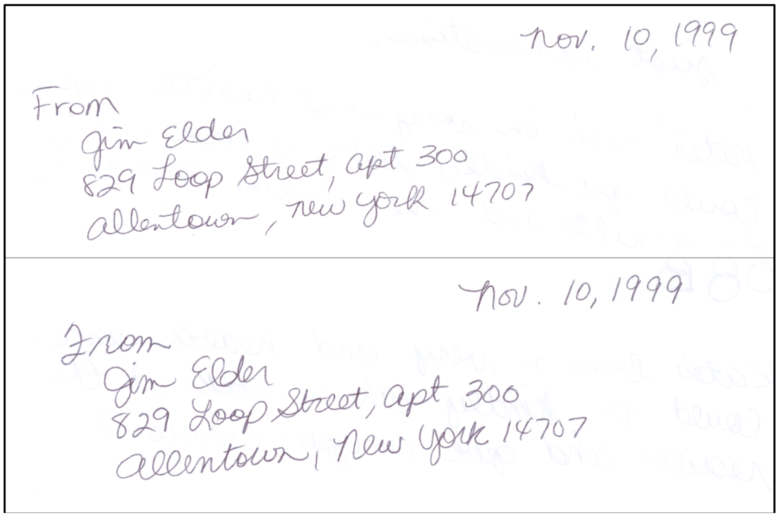

Fig S1. Tops of samples from which QK472 (Figure 3 in the main paper) was cropped. Note these samples also show (faintly) reverse text that was removed in image preparation. The remaining portion of the letter in both samples carried over onto another page; the bottom of the samples was not cropped.

Appendix B Previous Work

The accuracy and reliability of decisions made by FDEs have been the focus of several studies over the years (Table S7), which each vary in purpose, scope, and experimental design (including conclusion scales) and thus preclude direct comparison of their results with this study. Synopses for some of these earlier studies are presented below for the purposes of illustrating the difficulties in comparing error rates.

	Conclusion Scale	*Open Set*	*# Total Writers*	*Test Packets*	*# of FDEs*
Handwriting					
(Kam, Wetstein, & Conn, 1994) (10)	Sort into piles of same writer	No	20	86 Q, 20 K	7
(Kam, Fielding, & Conn, 1997) (11)	ASTM standard E1658 (9 level scale)	Yes	150	6 Q, 24 K	105
(Durina & Caligiuri, 2009) (12)	Forced to call identification or elimination	No	52	43 Q, 52 K	49
This study (2021)	5 level scale (modeled after proficiency tests)	Yes	230	180 total,<br>100 per participant	86
Cursive only					
(Kam & Lin, 2003) (13)	ASTM standard E1658 (9 level scale)	Yes	150	6 Q, 24 K	105
Hand printing only					
(Kam & Lin, 2003) (13)	ASTM standard E1658 (9 level scale)	Yes	150	6 Q, 24 K	105
(Mitchell & Merlino, 2016) (14)	ASTM standard E1658 (9 level scale)	Yes	Not specified	25 Q, 3 K per packet	53
Signatures					
(Kam, Gummadidala, Fielding, & Conn, 2001) (15)	Genuine, nongenuine or indeterminable	Yes	64	6 Q, 1 K	69
(Sita, Found, & Rogers, 2002) (16)	Genuine, simulated, or inconclusive	Yes	10	150 Q, 10 K	17

Table S7. Results summary for previous work as compared to the currently study.

Appendix C Survey Responses

Appendix C1 Participant background survey responses

The percentages below are based on 86 participants and may not sum to 100% due to rounding.

**Select the following statement which best describes your employment as an FDE.**			
	I'm currently employed as an FDE.	85	99%
	I'm not currently employed as an FDE, but I have been within the last 2 years.	1	1%
	I'm not currently employed as an FDE, but I was more than 2 years ago.	0	0%
	I'm an FDE trainee (and I have never been qualified in handwriting examination by a previous employer or certified in handwriting examination by an external certifying body).	0	0%
	I was never employed as an FDE.	0	0%
	Prospective participants who answered any of the last 3 options for question 1 were not permitted to participate in the study.		
**1. How old are you?**			
	Under 30	3	3%
	30-39	24	28%
	40-49	24	28%
	50-59	21	24%
	60+	14	16%
**2. Select all levels of education completed and provide the primary field associated with each degree.**			
	Highest degree		
	High school diploma (or equivalent)	3	3%
	Associate degree	3	3%
	Bachelor's degree	35	41%
	Master's degree	45	52%
	Doctoral degree	0	0%
**3. Provide the number of years of experience you have as an FDE, after completing your FDE training.**			
	<1	0	0%
	1-4	9	10%
	5-10	26	30%
	11-15	8	9%
	16+	43	50%
**4. Select the statement that best describes the frequency with which you currently perform handwriting examination casework? (not to include training)**			
	I conduct handwriting examination casework daily. (...)	35	41%
	I conduct handwriting examination casework a few times a week. (...)	22	26%
	I conduct handwriting examination casework a few times a month.	18	21%
	I conduct handwriting examination casework a few times a year.	7	8%
	I no longer conduct casework but have previously conducted handwriting examinations a regular basis.	1	1%
	I have never conducted handwriting examinations on a regular basis	0	0%
	(No response)	3	3%
**4A. What percentage of your typical work week is spent conducting handwriting comparisons?**			
	1-25%	5	6%
	26-50%	8	9%
	51-75%	16	19%
	76-100%	31	36%
	Question 4A was only asked of participants who conduct handwriting exams daily or weekly, but due to a software error, also includes responses from 3 participants who did not record a response to question 4. Percentages are based on all 86 participants.		
**4B. About how many handwriting comparisons do you conduct in a typical month?**			
	Less than 10	29	34%
	10-20	21	24%

20-40	7	8%
More than 40	0	0%

Question 4B was only asked of participants who replied to question 4 that they conduct handwriting exams daily or weekly.

5. Of your forensic handwriting casework, approximately what percentage falls in each of the following categories? (should total 100%)

	Criminal cases (prosecution side)	Criminal cases (defense side)	Civil cases	Other (e.g. administrative)
Min	0	0	0	0
Q1	30	0	0	0
Mean	70	0	10	0
Median	70	0	10	0
Q3	100	10	40	10
Max	100	50	90	100

6. How often have you testified as an expert in handwriting examination in a legal setting (including depositions and administrative hearings)?

Never	9	10%
1-9 times	31	36%
10-19 times	11	13%
20+ times	35	41%

7. Select the statement(s) that best describes your training. (Check at least one, and all that apply.)

I completed a formal, in-person program of instruction for 2 years or more. (...)	69	80%
I completed a formal, in-person program of instruction <2 years. (...)	9	10%
I received informal, on-the-job training.	17	20%
I completed courses and/or workshops.	21	24%
I completed correspondence courses (i.e. the trainer was distant)	4	5%

7A. Who was primarily responsible for providing your formal program of instruction in handwriting examination?

Current employer (...)	44	51%
Previous or past employer (...)	27	31%
An agency or company other than your current employer where you were not employed (...)	5	6%
Self-trained	3	3%

Question 7A was only asked of participants who replied to question 7 that they completed a formal, in-person program of instruction. Percentages are based on all 86 participants.

7AA. Select the category which best describes the agency or company responsible for providing your formal program of instruction in handwriting examination.

U.S. local agency	11	13%
U.S. state agency	16	19%
U.S. federal agency	25	29%
U.S. private company	2	2%
International government agency (i.e. nonUS)	22	26%
International private company	0	0%

Question 7AA was only asked of participants who replied to question 7A that they were not self-trained. Percentages are based on all 86 participants.

8. Are you certified as a handwriting examiner by an external certifying body?

Yes (...)	41	48%
No, I was previously certified (...)	2	2%
No, but I have been qualified by a current or former employer	39	45%
No, and I have never been certified or qualified by my current or former employer	4	5%

8A. Provide the certifying body for any current or past forensic document certifications. (Check all that apply. Check at least one.)

American Board of Forensic Document Examiners (ABFDE)	29	34%
Board of Forensic Document Examiners (BFDE)	1	1%
International School of Forensic Document Examination (ISFDE)	0	0%
National Association of Document Examiners (NADE)	1	1%
Scientific Association of Forensic Examiners (SAFE)	1	1%

American College of Forensic Examiners International (ACFEI)	1	1%
International Association of Document Examiners (IADE)	3	3%
Other	12	14%

Responses entered as text under "Other":

• Chartered Society of Forensic Sciences	1	1%
• Council for the Registration of Forensic Practitioners	1	1%
• Forensic Science Society - UK	1	1%
• Gesellschaft fur Forensische Schriftuntersuchung (GFS)	1	1%
• License from Texas Forensic Science Commission	1	1%
• previously I was a member of IADE	1	1%
• qualified assessor for ASCLD/LAB 2012	1	1%
• Taiwan Accreditation Foundation	1	1%
• Taiwan Accreditation Foundation (TAF)	1	1%
• Texas Forensic Science Commission (TFSC)	1	1%

Question 8A was only asked of participants who replied to question 8 that they are or were previously certified. Percentages are based on all 86 participants.

9. Did you ever testify as an expert in handwriting examination while still in handwriting training?

Yes	5	6%
No	81	94%

10. When did you last complete a proficiency test in handwriting examination?

Within the past year (...)	77	90%
Within the past 2 years (...)	5	6%
Within the past 5 years (...)	0	0%
More than 5 years (...)	2	2%
Never	2	2%

10A. Who prepared your proficiency test(s)? (Check all that apply. Check at least one.)

Employer (internal)	7	8%
Collaborative Testing Services (CTS)	70	81%
Forensic Science Foundation (FSF)	0	0%
European Network of Forensic Handwriting Experts (ENFHEX)	13	15%
National Institute of Forensic Science (NIFS)	0	0%
Other	8	9%

Responses entered as text under "Other":

• Association	1	1%
• Gesellschaft fur Forensische Schriftuntersuchung (GFS)	1	1%
• Hong Kong SAR Lab (Rubber Stamps) and handwriting	1	1%
• IADE	3	3%
• In house	1	1%
• ST2AR	1	1%

Question 10A was only asked of participants who replied to question 10 that they had completed proficiency tests. Eight participants indicated two proficiency test providers; three indicated three proficiency test providers.

11. Provide an estimate of the percentage of your work that involves cursive, printing, signatures, or mixed forms of handwriting. (The sum of all responses should equal 100.)

	Cursive	*Printing*	*Signatures*	*Mixed*
Min	0	0	0	0
Q1	10	10	20	10
Mean	16.6	19.5	36.7	27.0
Median	20	20	35	20
Q3	20	30	50	38
Max	60	60	100	100

12. Is your practice (i.e. you and/or your organization) involved with the following forensic science associations, as members or frequent attendees? (Check all that apply. Leave blank if none.)

American Academy of Forensic Sciences (AAFS)	48	56%
American College of Forensic Examiners International (ACFEI)	0	0%

	Association of Forensic Document Examiners (AFDE)	2	2%
	Australian Society of Forensic Document Examiners (ASFDE)	6	7%
	European Network of Forensic Handwriting Science Institutes / European Documents Experts Working Group (ENFSI/EDEWG)	14	16%
	International Association for Identification (IAI) Questioned Document Section	13	15%
	International Association of Document Examiners (IADE)	4	5%
	International Graphonomical Society (IGS)	1	1%
	Midwestern Association of Forensic Scientists (MAFS) Questioned Document Section	24	28%
	National Association of Document Examiners (NADE)	1	1%
	New Zealand Forensic Laboratories	0	0%
	American Society of Questioned Document Examiners (ASQDE)	48	56%
	Canadian Society of Forensic Science Questioned Document Section	5	6%
	Mid-Atlantic Association of Forensic Scientists (MAAFS) Questioned Document Section	22	26%
	Scientific Association of Forensic Examiners (SAFE)	2	2%
	Southeastern Association of Forensic Document Examiners (SAFDE)	17	20%
	Southwestern Association of Forensic Document Examiners (SWAFDE)	15	17%
	Special Advisory Group on Document Examination - Senior Managers of Australia	0	0%
	Other:	13	15%
	Responses entered as text under "Other":		
	• ABFDE	3	3%
	• ASB, OSAC	1	1%
	• Asian Forensic Sciences Network (AFSN, QDE working group)	1	1%
	• Asian Forensic Sciences Network (AFSN)	1	1%
	• ENFSI/ENFHEX	1	1%
	• Gesellschaft f¸r Forensische Schriftuntersuchung (GFS)	1	1%
	• Hellenic Association of Forensic Sciences	1	1%
	• NEAFS	1	1%
	• none	1	1%
	• OSAC	2	2%
	Number of associations checked:		
	• 0 associations	6	7%
	• 1 associations	24	28%
	• 2 associations	17	20%
	• 3 associations	12	14%
	• 4 associations	12	14%
	• 5 associations	6	7%
	• 6 associations	5	6%
	• 7 associations	1	1%
	• 8 associations	2	2%
	• 10 associations	1	1%
**13. What scale (or categories) do you use in reporting your conclusions?**			
	SWGDOC 2013 Standard Terminology for Expressing Conclusions of Forensic Document Examiners? (9 levels: Elimination, Strong probability did not, Probably did not, Indications did not, No conclusion, Indications to suggest, Probable, Strong probability, Identification)	46	53%
	Another scale with at least 7 levels (…)	21	24%
	Another scale with 5-6 levels (…)	17	20%
	Another scale with 3-4 levels (…)	1	1%
	A 2-level scale (such as Identification vs Exclusion) (…)	1	1%
	We do not use a categorical scale (…)	0	0%
	Responses entered as text (Another scale with at least 7 levels):		
	• "7 point"		
	• "11 levels, Kˆller / Nissen / Riefl / Sadorf: Probabilistische Schlussfolgerungen in Schriftgutachten, 2004"		
	• "A pseudo evaluative reporting approach"		

- "ASTM 1658-08 (same terminology as SWGDOC)"
- "Conclusive - Strong - Limited - Inconclusive (both positive/negative)"
- "Conclusive positive, strong, limited, inconclusive, limited, strong, Conclusive negative."
- "Elimination, Probably Did not, Indications Did not, No Conclusion, Indications to Suggest, Probable, Identification *(2 participants)*"
- "Elimination, probably did not, indications did not, no conclusion, indications to suggest, probably by, identification"
- "Identification - Probably wrote - Indications wrote - Neither Nor - Indications did not write - probably did not write - Elimination"
- "Identification, Highly Probable, Inconclusive, Not a basis for identification,Highly probable not, Elimination"
- "Remove strong probability did/did not from the 9 pt scale."
- "Same as SWGDOC 2013 but without Probably did not and Probable."
- "SWGDOC 2013 Standard Terminology for Expressing Conclusions of Forensic Document Examiners (7 levels: Elimination, Probably did not, Indications did not, No conclusion, Indications to suggest, Probable, Identification) *(4 participants)*"
- "SWGDOC 2013 Standard Terminology for Expressing Conclusions of Forensic Document Examiners (7 levels: Elimination, Strong probability did not, Indications may not have written, No conclusion, Indications may have written, Strong probability, Identification)"
- "SWGDOC 2013 Standard Terminology for Expressing Conclusions of Forensic Document Examiners (7 levels: Elimination, Strong probability did not,Indications did not, No conclusion, Indications did,Strong probability did, Identification"
- "The evidence provides moderate support for proposition X over proposition Y."
- "1) AUTOGRAPHY [In absence of limits to the survey and fundamental differences, valued the risks of complexity, elements which are decisive to the support of the theory that the considered subject has traced the text which is object of verification] ñ [It is the highest level of confidence expressed by the expert who has no reserves and is certain, on the basis of the analytical evidence, that the handwritings in comparison have the same origin] 2)PROBABLE AUTOGRAPHY [In absence of fundamental differences, strong elements to support of the hypothesis that the considered subject has traced the text which is object of verification] ñ [The evidence of the data allows the expert to consider probable that the handwritings in comparison have the same origin] 3)INDICATIONS OF AUTOGRAPHY [In absence of fundamental differences, weak elements to support of the hypothesis that the considered subject has traced the text which is object of verification] ñ [It is the lowest level of confidence expressed by the expert to whom the analytical evidence suggests that the handwritings in comparison can have the same origin] 4)NO CONCLUSION [Qualitative limitations of the graphic materials to be identified or in comparison or absence of homogeneous terms of comparison] ñ [It is the zero point of the scale of confidence. The analytical evidence doesn't permit the expertise to reach any conclusion] 5)INDICATIONS OF ETEROGRAPHY [Weak elements to support the hypothesis that the subject considered hasn't traced the text which is object of verification; analogies can be present] ñ [It is the lowest level of confidence expressed by the expert to whom the analytical evidence suggests that the handwritings in comparison have a different origin] 6)PROBABLE ETEROGRAPHY [Strong elements to support of the hypothesis that the subject considered hasn't traced the text which is object of verification; analogies can be present] ñ [The evidence of the data allows the expert to consider probable that the handwritings in comparison have a different origin] 7)ETEROGRAPHY [In absence of limits to the survey, found fundamental differences, decisive elements to support of the hypothesis that the considered subject hasn't traced the text which is object of verification; analogies can be present] ñ [It is the highest level of confidence expressed by the expert who has

no reserves and is certain, on the basis of the analytical evidence that the handwritings in comparison have a different origin] - (This scale of conclusion is being reviewed and implemented in the levels)”

Responses entered as text (Another scale with 5-6 levels):

- “5 levels: ID, MH, NC MNH, Elimination”
- “5 levels: Identification, May have prepared, No conclusion, May not have prepared, Elimination”
- “5 levels:Elimination, probably did not write, no conclusion, probably did write, identification”
- “5 point scale: ID, MH, NC, MNH, Elimination”
- “A scale made by dr.Sabol from Croatia, its in use in states from ex Yugoslavia.”
- “Elimination, Characteristics are different , No conclusion, Need to collect more standard writing materials, Characteristics are the same, Identification”
- “Elimination, Strong probability did not, No conclusion, Strong probability, Identification (2 participants)"
- “Identification, May Have, No Conclusion, May Not Have, Elimination” *(2 participants)*
- “Identification, Probably wrote, No conclusion, Probably did not write, Elimination”
- “Identification/May Have Prepared/No Conclusion/May Not Have Prepared/Elimination”
- “Modular Method”
- “nc, may have, ID, nc, may not have, elimination”
- “We use the SWGDOC 2013 scale, however, we only use five levels of the scale (identification, probable identification, unable, probable elimination, elimination).”

14. Do you use likelihood ratios or other probability measures in reporting conclusions?

Yes: we use likelihood ratios or other probability measures in addition to (or in support of) the conclusion scale	9	10%
Yes: we use likelihood ratios or other probability measures instead of a conclusion scale	1	1%
No	76	88%

15. Does your practice require a verification of handwriting examination conclusions (by a second handwriting examiner)?

Yes (...)	65	76%
No	21	24%

15A. Which conclusions require verification?

All conclusions	52	60%
Identification and Exclusion only	7	8%
Identification only	3	3%
Other	3	3%

Question 15A was only asked of participants who replied to question 15 that verification is required.

Responses entered under “Other”:

- “Criteria of number of knowns/questioned writings”
- “Probable through identification or elimination”
- “We have two indenpendent examinations”

15B. How are the verifications performed? (Check all that apply)

Verifier knows primary examiner’s conclusion	49	57%
Blind (verifier does not know primary examiner’s conclusion)	30	35%
Double blind (verifier believes he/she is primary examiner)	1	1%

Question 15B was only asked of the 65 participants who replied to question 15 that verification is required.

Combinations:

• Verifier knows conclusion (only)	35	41%
• Blind (only)	16	19%
• Double blind (only)	0	0%
• Verifier knows conclusion AND Blind	13	15%
• Verifier knows conclusion AND Blind AND Double blind	1	1%

Note that one participant who indicated they only conduct blind verifications also indicated (in #15A) that only identification conclusions are verified.

16. Does your practice/organization require a technical review of handwriting examination conclusions? (Technical review includes a review of all of the case documentation.)

Yes, always (…)	60	70%
Yes, but only for Identification and Exclusions (…)	3	3%
No	18	21%
Other (…)	5	6%

Responses entered as text under "Other":

- "(I disagree with the question's definition of Tech Review and feel it is confused with Admin Review)"
- "60-100%, based on primary examiner's experience"
- "Periodically I exchange cases for review with peers in private work."
- "sometimes"

Ten participants (12%) indicated they require neither technical review (#16) nor verification (#15).

16A. Does the technical reviewer have to be a qualified handwriting examiner?

Yes	66	77%
No	2	2%

Question 16A was only asked of participants who replied to question 16 that technical review is required. Percentages are based on all 86 participants.

16B. Is the technical reviewer the same person as the verifier?

Yes, always	34	40%
Yes, sometimes	12	14%
No	22	26%

Question 16B was only asked of participants who replied to question 16 that technical review is required. Percentages are based on all 86 participants.

17. Does your handwriting practice require continuing training and education?

Yes	79	92%
No	7	8%

18. Does your practice require handwriting proficiency tests?

Yes	78	91%
No	8	9%

19. Does your practice view the examination of photocopies or photographs as a limitation?

Yes, always (...)	39	45%
Sometimes (…)	42	49%
No	5	6%

19A. Please explain how the examination of photocopies or photographs is a limitation.

Text responses received:

- "1) it's not possible to establish if the "original" document exists or if it is a copy from digital composition (scanned + copy and paste);2) it's impossible and not reliable to express judgments about the pressure, the quality of the line and the quality of start and end stroke, the connections between (inter) and intra letters, the type of pen used, the presence of traced and so on."
- "as a rule examiners won't eliminate or identify a writer when the writing submitted is nonoriginal since the reprographic process can conceal evidence of manipulation and obscures detail; the underlying objective is to reduce or eliminate false conclusions due to artifacts of the copying process appearing in the nonoriginal writing or due to a forger concealing false writing via the copying process"
- "As we are not able to fully visualize the line quality and pen pressure, photocopies and photographs are seen as a major limitation during our examinations."

- "Assess quality and clarity to determine if having a photocopy or photograph is a limitation and factor accordingly. Include notes in case file and report wording to address limitations."
- "because it precludes a thorough examination of the traits of handwriting to include pen pressure, direction, pen lifts, etc."
- "Because photocopies or photographs can be manipulated, one cannot be absolute in the conclusion."
- "Can not rule out cut and paste manipulations, limited clarity and detail."
- "Can't always see pen pressure, indentations, direction of stroke, etc."
- "Can't conduct latent writing impression examinations, pre-liminary ink analysis, and limited on handwriting examination microscopic analysis."
- "Cannot determine pen pressure, true connecting strokes, number of strokes to complete a character, possibility of manipulation."
- "Categorical conclusions need original documents"
- "Certain dynamic characteristics of handwriting, such as speed, pen lifts, etc. are not necessarily reproduced in photocopies. Furthermore, it is possible to leave behind minimal evidence of a digital insertion in a created/altered document hence the necessary limitation when examining a copy that the copy must be representative of some original, writing ink on paper document."
- "Check the characteristics of handwriting are clear or able to be recognized."
- "Copies may not adequately depict critical features for proper assessment of similarities/ dissimilarities. Copies may be cut-and-paste creations and depict documents that do not exist."
- "Dependent upon the quality and resolution of the submitted documents"
- "Depending on the quality of the copy or image, we may only be able to offer a qualified opinion (nondefinitive) or be inconclusive."
- "Depending on the quality of the photocopy or photograph, the conclusions for identification or exclusions will be limited by the clarity of the examined writing. Sometimes the quality of such documents may be so poor that a conclusion can not be made until an original or better photocopy has been provided for examination."
- "Depending on the quality of the photograph/photocopy, being able or unable to see pen movement, speed, other characteristics, etc."
- "Depends on quality of photocopies."
- "Depends on the nature of the copy but it is usually a limitation because the fluency and fine detail cannot be assessed. However there may be instances when a conclusion is possible so it is not always an issue."
- "Depends on the quality"
- "Depends on the quality and detail present in the submitted documents for examination."
- "Depends on the quality and the question being asked."
- "Depends on the quality of the reproduction."
- "Details of the writing act are often masked by the photocopying process."
- "Due to lack of detail and possible misinterpretation of foreign matter reflected in copies we do not base positive identification or elimination on reproductions."
- "Each case is evaluated for quality of photocopies or photographs, when provided."
- "Examination of photocopies is not possible if the quality of the copies is very poor."
- "Examination of photocopies is not possible, if the quality of photocopies is very poor."
- "I do not examine handwriting cases from photocopies because of the lack of their clarity. On the other side I always -try to- perform microscopic examination on handwriting cases. Photocopies can be examined if there is a question concerning the identification of an individual photocopier or other related subject."
- "Identification and Elimination are no longer available conclusions due to possibility of digital manipulation, etc."
- "Identification of copies are reported as "virtually certain ..."“
- "If photocopies or photographs are not of good quality or resolution, then this will be viewed as a limitation and subsequently reflected in the report through a qualified conclusion."
- "If the authenticity of the document bearing the questioned handwriting cannot be established by some legally acceptable means, then the handwriting will be eliminated or identified at a probable level of certainty.The handwriting, handprinting, numbers, or signatures may be identifiable, but their placement on the questioned document remains uncertain; therefore, the opinion is lowered, as a rule, giving notice of the problem of authenticating the document as a whole. When asked, it is explained that cut-and-paste cannot be eliminated as the means of placing the identifiable writing on the photocopy, print-out, or photograph.Of course, if alignment defects of the questioned writing vis a vis the formatted document are detected, the opinion may be inconclusive or some level of elimination, despite the identifiable writing. The alignment defects provide evidence of cut and paste."
- "If the images are not clear enough for examination"
- "If the photocopies are very unclear."
- "In case of the photocopies are very unclear."
- "In the case of poor quality copies when all relevant details cannot be discerned, the examination can be limited to such an extent that a definite conclusion cannot be reached.The possibility that forgery or cut and paste of signatures took place cannot be excluded on copies and limit the conclusion."

- “It can limit the examination of speed, presure, rythm etc”
- “It depends about the photocopy quality.The opinion/finale conclusion never be the 100 %...it is only on the probability scale of the conclusions.”
- “It depends on the image quality of the copies. More often there is a limitation, but sometimes we receive really nice copies that are very good image quality.”
- “Lack of clarity and detail.”
- “Lack of fine detail and possibility of manipulation/transplanting”
- “lacks details of the original document, such as pen pressure and fine detail (drag marks).Cannot conduct indented writing examinations, or determine tracing, writing instrument, etc.”
- “limited examination possibilities”
- “May lack detail and clarity; Cannot be sure writing wasn't digitally or manually manipulated onto nonoriginal; copies may mask simulation or tracing characteristics”
- “Nonoriginal questioned document nearly always a limitation - limits characteristics such as pen pressure, stroke sequence, & masks evidence of simulation, tracing, & manipulations. Nonoriginal known writing may be a limitation depending on the quality of the image/copy.”
- “Nonoriginals can mask the detail needed for a full examination and could also contain unknown manipulation.”
- “Photocopies and images often mask fine details and characteristics that may not be readily discernible depending on the quality of the copy or image. This often limits the ability to be conclusive or even to observe characteristics indicative or tracing or simulation”
- “Photocopies and photographs are a 2-dimentional representation of a 3-dimentional body of writing. It lack representation of pen pressure and may lack detail and clarity with regards to connection strokes, number of strokes, guide lines (traced signatures), or other relevant information for the handwriting comparison.”
- “Photocopies and photographs do not always show the characteristics of pressure and pen movement.”
- “Photocopies do not show all of the fine and subtle details that original writing shows.”
- “photocopies may have been tampered with, and may not represent the original document. Details of handwriting strokes, ball striations may not be visible on photocopies or photographs”
- “Photocopies or photographs limit the examination when the quality of the copies/photographs prevent the examination of subtle details in the writings”
- “Photocopies or photographs may not depict small details or characteristics. They can sometimes mask features associated with simulations or tracings, and cut and paste type scenarios whether digital or physical. However, a good quality photocopy or photograph can still provide you with enough information to give a conclusion, even if it is not conclusive.”
- “Photocopies/Photographs limit the determination of naturalness of the writing and at times limits the ability to recognize slow and deliberate writing.Details such as pen stops/lifts/hesitations, tremor in the line and pen pressure variation can be hidden.Also, characteristics of a tracing can also be hidden (guidelines, ink on the back of documents...)”
- “Photographs are not a limitation but photocopies can be, especially, if they are multi-generational.Photocopies can get distorted and they may obscure details.”
- “Photographs may be out of focus, too far away, or poorly illuminated. Copies may be of poor quality and in B/W.”
- “Poor line quality which can hide simulations and tracings and digital manipulations”
- “Research that contains graphs and documents presents difficulties and inspires care, because unlike an original document, it is not possible to verify the erasures, traces of washes, splices, additions and decals, and it is not possible to determine accurately. dash crossings, overlays, as well as the type of recording device used (pencil, pen, etc.), including some aspects of graphics, typewriters and / or montages.”
- “Resolution dependent”
- “Some features, such as fluency, may not be as clear within photocopies. Photocopies may not show features of tracing etc.”
- “The clarity is affected by the photocopying process and one cannot state that the handwriting/signature was originally placed on the original with an examination of the original.”
- “the examination of photocopies or photographs is a limitation becausethe examination of the line under magnification is a determining element. A copy does not allow the study of the line. We do not know if the document has been falsified, such as a montage”
- “The fine details of the written line cannot be fully observed as when examining the original. In addition, a allows for digital manipulation.”
- “The opinion is always qualified, "...with the best evidence available".”
- “The photocopying process may mask signs of manipulation/alteration and may limit the ability the discernment of fine line details”
- “The quality of the copy can place a limitation the examination and comparison.”
- “The quality of the photocopy or photograph may be a limitation in the examination. In addition, detail of the writing may not be captured by the photocopying or photography process.”
- “The quality of the reproduction copy may be a factor i.e. clarity and detail.”
- “Unable to exam for characteristics such as indented outlines, overwriting and breaks in line”

- "unclear photocopies"
- "we do not give deciduous opinions on photocopies or photographs"
- "We will conduct handwriting comparisons on photocopies/photographs etc., however, depending on the quality/clarity of the copy, this may limit the comparison and therefore the conclusion given in the case."
- "when line quality is important / when digital manipulation is suspected"
- "When the photocopies are very unclear"

20. If originals are not available, will you have the same range of conclusions?

Yes	51	59%
No (…)	35	41%

Text responses received:

- "a handwriting conclusion is usually reduced by one confidence level if originals are not present"
- "Because of the limitations stated above, the strongest finding on the scale would be a highly probable."
- "Cannot ID or eliminate without originals due to their limitations (as outlined in 19a)"
- "Conclusions will not be definitive;for example: May Have or May Not Have been prepared by the writer."
- "Decrease the range of conclusions."
- "Depending on the image quality of the copies, the strongest conclusion may be highly probable."
- "Depending on the quality and nature of the copied documents."
- "Depends on the quality"
- "Examining a photocopy, we can say if the signature matches thecomparison signatures.But it can't be said, that the document is signed by the person.We can't exclude the possibility of the signature to be tranferred on the document using some technical means. The opinion for photocopies is always weaker than it would be in the case of originals."
- "I conclude with caution, as the expert may have been taken to error by not examining the original. Research that contains graphs and documents presents difficulties and inspires care, because unlike an original document, it is not possible to verify the erasures, traces of washes, splices, additions and decals, and it is not possible to determine accurately. dash crossings, overlays, as well as the type of recording device used (pencil, pen, etc.), including some aspects of graphics, typewriters and / or montages."
- "I generally do not render conclusive opinions on photocopies."
- "Identification opinion with be accompanied by a qualification statement."
- "If originals are not available the opinion is subject to examination of the originals. Photocopies can mask cut and paste documents thereby limiting our ability to render a positive identification."
- "If the Q is nonoriginal a photocopy is a limitation. If the K is nonoriginal I might still have the same range of opinions depending on the quality of the K."
- "If the questioned item is not original, an identification will not be made, rather a more conservative opinion of may have prepared would be rendered."
- "In that case (not originals available) my conclusions are always qualified, expressing opinions of lesser certainty, giving -several- factors which can affect the outcome of the given case."
- "In the procedure, original documents are required to issue a categorical conclusion"
- "It will then be necessary to make reservations"
- "limitation of the identification"
- "No definitive conclusions"
- "Normally the range of conclusions were decreased if originals are not available."
- "Only original is appropriate for full examination."
- "rarely make identification"
- "see 19a"
- "See 19a."

- "The conclusion will be limited, we must consider that document was forged."
- "The examination of a reproduction requires the acknowledgement of the inherent limitation that comes with the examination of a nonoriginal document."
- "The photocopying analysis can never reach level 1 and level 7 of the conclusions scale (which is being reviewed and implemented in the levels)"
- "The range of conclusions does not change; however, I would not identify without original handwriting available."
- "The range of conclusions were decreased."
- "The range of conclusions will be decreased."
- "there are inherent limitations due to the nonoriginal nature of the items being examined and a qualified examiner knows what those limitations are and more importantly that there are situations where it's not possible for anyone to reach a definitive conclusion (i.e., nonoriginal writing) though it may be possible to reach a qualified conclusion"
- "we do not give deciduous opinions on photocopies or photographs, but we use other 7 levels"
- "Yes, but with limitations explained"

21. Please indicate how often you make the following assessments regarding handwriting evidence, in written reports and/or during testimony (check the frequency for each).

	Often	*%*	*Rarely*	*%*	*Never*	*%*
Age of Writer	14	16%	22	26%	50	58%
Alterations/erasures/obliterations	69	80%	17	20%	0	0%
Association or NonAssociation with a writer	76	88%	6	7%	4	5%
Educational level	4	5%	16	19%	66	77%
Gender	2	2%	7	8%	77	90%
Handedness	12	14%	21	24%	53	62%
Haste/speed of writing	66	77%	18	21%	2	2%
Intentionally distorted known writing	40	47%	41	48%	5	6%
Intentionally distorted questioned writing	46	53%	34	40%	6	7%
Mental state	3	3%	19	22%	64	74%
Natural vs unnatural writing	68	79%	15	17%	3	3%
Original vs nonoriginal document	80	93%	6	7%	0	0%
Personality traits	2	2%	5	6%	79	92%
Skill level of writer	67	78%	18	21%	1	1%
Tracing or simulation	59	69%	26	30%	1	1%
Veracity/Truthfulness	11	13%	10	12%	65	76%

Assessments that were highly correlated (based on correlation clustering):

- Gender, educational, veracity, age, mental state, personality, handedness
- Intentional distortion (questioned), intentional distortion (known), natural vs unnatural
- Skill, speed, original vs nonoriginal, trace or simulation, alterations

22. Select the category which best describes your current AND all past employer(s). (Check all that apply. Check at least one.)

U.S. local agency (...)	16	19%
U.S. state agency (...)	18	21%
U.S. federal agency (...)	31	36%
U.S. private company (...)	3	3%
NonU.S. government agency (i.e. nonUS) (...)	23	27%
NonU.S. based private company (...)	6	7%
Currently unemployed or retired	0	0%
Academic institution (...)	7	8%
Sole practitioner / private practice (...)	21	24%

Combinations:

• *U.S. local, state, or federal agency (i.e. 11 selected 2 of these categories)*	*54*	*63%*
• *Private company (U.S. or non-U.S.) or Sole practitioner / private practice*	*29*	*34%*

22A. Is your current practice accredited?

Yes, with ANAB or ASCLD/LAB	48	56%
Yes, with another accrediting body (in accordance with ISO 17025) (…)	9	10%
Yes, with another accrediting body (but not under ISO 17025) (…)	1	1%
Unsure	3	3%

No	25	29%
**22AA. Who is the accrediting body?**		
(no text responses received)		
**23. Select the following statement which best describes the FDE(s) working at your current practice.**		
I'm the only FDE currently working.	21	24%
1 other FDE in addition to me currently working.	14	16%
2-4 other FDEs in addition to me currently working.	19	22%
5 or more other FDEs in addition to me currently working.	32	37%

Appendix C2 Participant post-test survey responses

The percentages below are based on the 66 participants who completed the post-test survey, and may not sum to 100% due to rounding.

**1. Overall, were the QUESTIONED samples in this study representative of your casework?**		
Strongly Agree	7	11%
Agree	44	67%
Disagree	12	18%
Strongly Disagree	3	5%
**2. Overall, were the KNOWN samples in this study representative of your casework?**		
Strongly Agree	3	5%
Agree	47	71%
Disagree	14	21%
Strongly Disagree	2	3%
**3. How does the overall difficulty of the comparisons in this study correspond to your casework?**		
Easier	8	12%
Similar	53	80%
Harder	5	8%
**4. When you performed comparisons in this test, did you do them in such a way that your results would have been the same if we had swapped which samples were labelled "questioned (Q)" and "known (K)"? In other words, when you do a comparison, is it symmetric, treating the Q sample the same as the K sample(s)?**		
Yes: results would be the same if Q and K were swapped	32	48%
It depends: whether I treat the Q and the K the same depends on the samples	28	42%
No: I always treat the Q and the K differently, so results may be different if Q and K were swapped	6	9%
**5. In casework, how often do you perform unknown-to-unknown comparisons?**		
Never	3	5%
Rarely	42	64%
Often	21	32%

Appendix D Study Instructions

This section includes the instructions provided to each participant.

Appendix D1 Overview

Appendix D1.1 Digital Subtest

As a participant in the Handwriting Examiner Decision Analysis Study (aka Handwriting Black Box Study), you will be asked to perform 100 handwriting comparisons over a period of approximately eight months, using digital images. For each comparison set, you will be asked to compare one questioned item with one or more known items of handwriting. The Handwriting Black Box Study (HBBS) website (https://handwriting.idealinnovations.com) will provide you access to the digital images as well as a user interface for reporting your conclusions.

Appendix D1.2 Physical Subtest

Participants in the Handwriting Black Box Study may optionally participate in the "Physical Subtest," which will be conducted in person using originals, but otherwise follows the same procedure as the Digital Subtest. The Physical Subtest will be conducted at document examination conferences, and in the Washington DC area. Details and instructions will be provided in a separate document and sent to all registered participants.

NOTE: the Physical Subtest was cancelled due to lack of sufficient participation.

Appendix D2 Eligibility and Registration

Participation in the study is open to examiners who have performed handwriting evidence comparisons in operational casework within the last two years, and make conclusions using a conclusion scale of at least 5 levels.

To register for the study, you must complete the following:

- Online registration form
- Online survey
- Signed research consent form

After these have been received, you will be provided a Participant ID, which you will use along with the password you create during the registration process every time you login to the HBBS website.

Appendix D3 Participation Conditions

When you first log into the HBBS site, you will be presented a "Terms and conditions" screen. To participate you must agree to the following:

- to conduct the comparisons in this study with the same regard and diligence used when conducting handwriting evidence comparisons in operational casework,
- not to conduct the comparisons in this study collaboratively,
- not to share or distribute the test materials associated with this study to anyone (including coworkers and colleagues),
- to discard all handwritten notes and/or printed materials prepared by you when you submit each comparison set, and
- to delete all digital images downloaded by you when you submit each comparison set.

Appendix D4 Comparison Sets

Fig S2 shows examples of two comparison sets. Each comparison set contains:

- A proof sheet showing exactly the images contained in the comparison set
- One questioned document, which will be up to one page.

- One to six known documents, each of which will be up to one page. All of the known samples in a given comparison set are definitively from a single person.
- The questioned and known documents will include the same text in about half of the comparisons.

To the best of our knowledge all samples included were freely and naturally prepared.

The quality of the images resulting from scanning of the samples varies significantly — as it does in casework. If the quality of a given sample affects your conclusion, please note that in the limitations (question #3). Note that handwriting samples from a given subject often used different imaging processes, so the digital image characteristics should not be a factor in your conclusions.

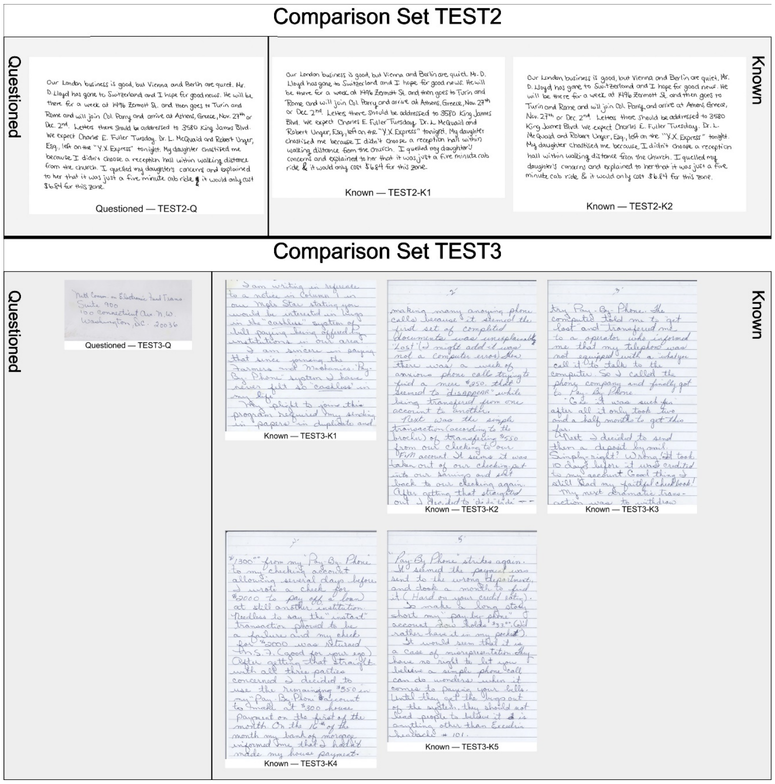

Fig S2. Examples of two proof sheets for comparison sets.

Appendix D5 Handwriting Black Box Software (HBBS) Website

The HBBS website (https://handwriting.idealinnovations.com) is accessible using an ordinary web browser. There is no need to download or install any additional software or plugins.

The HBBS website was built for Google Chrome (version 69), Internet Explorer (version 11), Edge (version 16), or Mozilla Firefox (version 62). Using older versions may result in errors. Safari is not supported.

In order to login to the HBBS website, you will need the Participant ID provided to you, and the password you created during registration. If necessary, use the “Forgot password?” link in the Participant Login form to reset your password.

If you leave the HBBS website, your answers will be saved. Until you submit your responses, they are changeable so you may return to a comparison set and continue working. Once you submit your responses for a comparison set, they are final and submitted to the study administrator. You may not access submitted comparison sets again.

The HBBS website allows you to download the digital images in either TIFF or JPEG formats. Both the TIFF and JPEG images are high quality and contain the same number of pixels, but the JPEG images are less than half the size of the TIFF images due to lossy compression.

Appendix D6 Comparisons

Each comparison set has a *QKset number* (QK001 through QK999) that you will use to associate the materials and your responses. The QKset number is on every image and proof sheet.

You report your decisions for each comparison set in the HBBS website. Your decisions will be recorded by responding to the questions and statements below. Note that your answers affect which questions you are shown: for example, question #2 is only applicable as a follow-up to some answers to #1, and is otherwise hidden.

In order to limit possible misunderstandings, please complete at least five comparisons before submitting any of them. The software will enforce this.

If any questions are unclear, please email the study administrator at handwriting@idealinnovations.com for clarification before submitting.

**#1 — Conclusion**		***Always shown***
1	Conclusion	*Select the conclusion that best characterizes your determination, based on the comparison of the questioned and known samples.*
1A	The questioned sample was written by the known writer	*This is the highest degree of confidence expressed by document examiners in handwriting comparisons that the writer of the known material wrote the writing in question. The conclusion is certain, with no reservations or doubts. (includes Identification)*
1B	The questioned sample was probably written by the known writer	*A less than definite opinion in which the evidence contained in the handwriting points toward the questioned and known writings having been written by the same individual; limitations should be indicated. (includes may have, strong probability, highly probable, very probable, probable, and indications)*
1C	No conclusion	*This is the zero point of the confidence scale. It is used when there are significantly limiting factors, and the examiner does not have even a leaning one way or another. (includes inconclusive, indeterminable)*
1D	The questioned sample was probably not written by the known writer	*A less than definite opinion in which the evidence points away from the questioned and known writings having been written by the same individual. (includes may not have, indications did not, probably did not, strong probability did not)*
1E	The questioned sample was not written by the known writer	*This is the highest degree of confidence expressed by the document examiner in handwriting comparisons that the questioned and known writings were not written by the same individual. The conclusion is certain, with no reservations or doubts. (includes elimination and exclusion)*
**#2 — Basis of exclusion**		***Only shown if (1) Conclusion = (1D) "Was probably not written by" or (1E) "Was not written by".***
2	For your conclusion of "not written by" or "probably not written by" was the basis:	*Select the option that best characterizes your reason for your conclusion of (1D) "Was probably not written by" or (1E) "Was not written by".*
2A	Primarily a holistic decision (e.g. based on class characteristics)	
2B	Primarily a decision based on individual characteristics (i.e. specific details)	
2C	A mix of both	
**#3 — Limitations**		***Always shown***
3	Limitations: Select any of the following factors if they limited the examination process in a way that affected your conclusion. (Leave blank if none)	*Please indicate any limitations that kept you from making a more definitive conclusion OR that were a notable source of difficulty in making the comparison.*<br><br>*Check all that apply. Leave blank if not applicable.*
3A	Clarity and detail of the questioned sample	*There is limited clarity, quality or reproduction of significant details in the questioned sample.*
3B	Clarity and detail of the known sample(s)	*There is limited clarity, quality or reproduction of significant details in the known sample(s).*

3C	Quantity of writing in the questioned sample	*There is an insufficient amount of writing in the questioned sample.*
3D	Quantity of writing in the known sample(s)	*There is an insufficient amount of writing required to assess the writer's range of variation.*
3E	Insufficient individualizing characteristics	*There are insufficient marks or properties in the specimens that uniquely characterize writing.*
3F	Comparability	*The questioned and known samples do not contain the same types of writing with similar characters, words, and combinations and/or the writing was not contemporaneous.*
3G	Presence of unexplained characteristics	*The questioned writing includes characteristics that were not demonstrated in the available known writing, but do not yet rise to the level of a difference.*
3H	Distorted writing	*The writing does not appear to be natural, but may be natural due to voluntary or involuntary factors.*
**#4 — Difficulty**		***Always shown***
4	Difficulty	*Select the option that best characterizes your assessment of the difficulty of this comparison.*
4A	Obvious (Very Easy)	*The conclusion was obvious.*
4B	Easy	*The comparison was easier than most comparisons.*
4C	Moderate	*The comparison was typical of most comparisons.*
4D	Difficult	*The comparison more difficult than most comparisons.*
4E	Very Difficult	*The comparison was unusually difficult.*
**#5 — Skill level**		***Always shown***
5	How would you assess the skill level of the questioned writer?	*Select the option that best characterizes your assessment of the skill level of the questioned writer.*
5A	High skill level	*The writer displays a high degree of letter consistency, uniformity of handwriting appearance, and general artistic qualities.*
5B	Medium skill level	*The writer displays some letter consistency, uniformity of handwriting appearance, and general artistic qualities.*
5C	Low skill level	*The writer generally does not display letter consistency, uniformity of handwriting appearance and very little artistic qualities.*
**#6 — Type of writing**		***Always shown***
6	Assess the type of writing in the questioned sample.	*Select the option that best characterizes your assessment of the type of writing in the questioned sample.*
6A	Predominantly disconnected (typical) hand printing	
6B	Connected hand printing	
6C	A mix of hand printed characters and cursive characters	*Use this category if none of the other categories apply.*
6D	Disconnected cursive	
6E	Predominantly connected (typical) cursive writing	
**#7 — Variation in Questioned sample**		***Always shown***
7	How much variation is there within the QUESTIONED writing? (this may include character formations, writing styles, etc.)	*Select the option that best characterizes your assessment of the amount of variation in the QUESTIONED sample.*
7A	Large amount of variation	
7B	Medium amount of variation	
7C	Limited amount of variation	
**#8 — Variation in Known sample**		***Always shown***
8	How much variation is there within the KNOWN writing? (this may include character formations, writing styles, etc.)	*Select the option that best characterizes your assessment of the amount of variation in the KNOWN sample(s).*

8A	Large amount of variation	
8B	Medium amount of variation	
8C	Limited amount of variation	
**#9 — Comparability**		***Always shown***
9	How comparable is the content of the writing in the questioned and known samples?	*Select the option that best characterizes the extent to which the questioned and known samples have comparable text content.*
9A	Same text (i.e. all of the questioned text is found in the known text)	
9B	Some words in common	
9C	Some numbers and/or letter combinations in common	
9D	No (or almost no) comparability	
**#10 — Most influential features**		***Only shown if (1) Conclusion is NOT (1C) "No conclusion"***
10	Check the two features that most influenced this conclusion: (check exactly two)	*Select the TWO features or attributes that were the most important considerations in making this conclusion. (Does not apply to (1C) "No conclusion".)*
10A	Abbreviations	A shortened form of a word or title.
10B	Alignment	The spatial organization of the writing pattern, its linear arrangement or words and intervening spaces and their accommodation on the page.
10C	Arrangement	The order or organization of a written inscription on a sheet.
10D	Baseline relationships	The relationship between letters and words upon the ruled or imaginary line upon which the writing rests.
10E	Connecting strokes	A line joining two adjacent characters
10F	Height relationships	A measurement of relative relationships between letters, component parts of letters, letter combinations, or words.
10G	Initial and/or terminal strokes	The first stroke leading into a character, word or signature/the final stroke leading out of a character, word or signature.
10H	Letter/number formation	The construction of a written character or numeral.
10I	Pen lifts	An interruption in a stroke caused by removing the writing instrument from the writing surface
10J	Pen pressure	The force with which the writing instrument contacts the paper
10K	Punctuation	The use, formation or placement of marks used in writing to separate sentences and clarify meaning.
10L	Rhythm	That element of the writing movement marked by regular or periodic recurrences (i.e. smooth, intermittent, uniform quality)
10M	Size	Ratio or proportion between two or more components of handwriting (i.e. letters and words.)
10N	Skill level	Degree of ability or proficiency of someone to write based on consistency, uniformity, writing instrument pressure, and general artistic qualities.
10O	Slant	The angle or inclination of the axis of letters relative to the baseline. Also referred to as slope.
10P	Spacing	The space between letters or words.
10Q	Use of symbols	The use, formation, and placement of a character that is used to represent something that might be expressed in one or more words.
**#11 — Need for original documents**		***Only shown if (1) Conclusion is (1B) "…probably written …", or (1D) "…probably not written…".***
11	Would your conclusion have been stronger if you had the original documents?	*In this particular comparison, if on review of the original documents your interpretation of features and attributes remained the same, would you change the "probably" or "probably not" conclusion you made to "written by" or "not written by"?*
11A	Yes	
11B	No	

#12 — Comments regarding conclusion		*Always shown*
	Comments regarding any issues not addressed by the other questions (optional) [TEXT]	*Please only comment if there is an issue or a limitation in this comparison set that you could not adequately address using any of your responses above. It is not necessary to say that you would prefer to have originals: we assume that it generally true.*

Appendix D7 Submitting Responses for a Comparison Set

After you respond to all of the questions and statements for a comparison set, press "Review and Submit" and the HBBS website will show a Final Review, which summarizes your responses. If your responses are incomplete, the Final Review will indicate the incomplete response(s); you will not be permitted to submit your responses until the incomplete responses are corrected.

Once you are satisfied with your responses, select "Submit". After you submit your responses, you may not access the comparison set again.

Appendix D8 Completion

Upon completion of all comparison sets, you will be required to complete a brief post-test survey, which will allow you to provide feedback on the testing experience, quality of the test materials, and the representativeness of the test materials. The HBBS website will present you with the post-test survey immediately after you submit the last comparison set in the final packet.

We strongly encourage you to complete all 100 comparisons.

To encourage participation, four participants who complete all assigned comparisons will be randomly selected and given awards: one $1000 and three $500 awards will be given. These will only be awarded after all results are collected: rapid completion will not improve the chances of winning. If your agency does not permit you to accept such awards (or donate them to charity), we will repeat the random drawing until we select someone who accepts the award.

If you start and are unable to complete all 100 comparisons, we will include results for any participants who complete at least 30 comparisons. However, keep in mind that by not completing all 100 comparisons you will not be eligible for the awards.

Appendix D9 Frequently Asked Questions (FAQ)

In order to guarantee that participants all received identical instructions during the study, no changes to instructions were made after the start of the test period. Any questions related to the study were answered in a Frequently Asked Questions (FAQ) document that was shared with all participants. The final version of the FAQ is included below (last updated 4 April 2019).

1. ***Is the study evaluating signatures?***
   No. Signatures are NOT included in the study.

2. ***Is it possible to give an estimation of the amount of time per examiner to perform 100 handwriting comparisons? (updated 14 Dec. 2018)***
   We expect the amount of time to vary significantly from examiner to examiner, as well as by the difficulty of the comparison. We assume in general 15-30 minutes per comparison, so the total time we expect would be 25-50 hours over 8 months — or 45 to 90 minutes per week over 8 months.

3. ***Are there details of a schedule or expected time frame? (updated 4 April 2019)***
   We expect to start the study in April 2019. Participants are allowed approximately eight months to complete the 100 comparisons ("Digital subtest"), which will be provided electronically.
   Participants are encouraged to participate in the Physical Subtest — see FAQ #16.

4. ***Will the participants in the study be limited to people who completed two or more years of formal training as document examiners?***

Participation in the study is open to examiners who have performed handwriting evidence comparisons in operational casework within the last two years. One goal of the study is to assess the relationship between accuracy and factors such as training: participants will complete a questionnaire assessing type and extent of training and experience.

5. ***Can you provide more details on the types of comparisons?***
   The questioned document will be up to one page. The known documents (all from one person) will be up to six pages. The questioned and known documents will include the same text in about half of the comparisons.

6. ***Description states one or more known samples. One or two is not a suitable known sample for a determination. A concern is that you may end up with higher errors if an FDE goes ahead and accepts working with such limited knowns, or your data is going to reflect a higher percentage of weak opinions or inconclusives due to the qualification of not having an adequate number of known samples.***
   Our data will include a wide range of data, as seen in casework. One of the purposes of the study is to measure how conclusions are affected by the amount of writing in the questioned sample, and the amount of writing in the known sample(s) — the study data will vary both of these. As you say, it is a reasonable assumption that responses will be less conclusive as these decrease — but we want to measure that, as well as assess the variability of examiners in how their responses are affected.

7. ***You are welcoming international examiners only if they use the 9 point scale or similar scale of at least 5 levels. Does that mean that U.S. examiners are welcome even if they don't use any of those scales?***
   Examiners (U.S. or not) are welcome if they use a scale of at least 5 levels. We will clarify the text in the announcement.

8. ***Since we have fought the battle long and hard with respect to the 9 point scale, why are participants required to use a 5 point scale? Assuming the results of this study are favorable to the extent that the FDE profession was bolstered by the results, it would appear that only the 5 level scale would be validated and not the 9 point.***
   The purpose is to assess the accuracy and reliability of examiners, not to validate a specific scale. If the 9-point scale were in universal use, we would certainly adopt that for the study. Unfortunately, since conclusion scales vary, we had to select a scale that would be understood by the greatest proportion of examiners.

9. ***Is the study open to nonEnglish speakers?***
   Because the questions and instructions are all in English, participants must be reasonably fluent in English to participate in order to minimize the potential for misunderstandings.

10. ***How will the results be reported?***
    Results will be published in a peer-reviewed journal, and presented at appropriate forensic conferences.

11. ***Can we find out our own results?***
    No: we are required to keep the results anonymous. We have processes in place so that results are anonymous even within the analysis team.

12. ***We will not be allowed to accept any gifts associated with the study.***
    We understand. If we randomly select someone who is unable to accept the award, we will repeat until we select someone who accepts the award.

13. ***Do you have an approximation on how much time this will take? I know the study will be conducted over 8 months, but I wanted to know if there is a better breakdown or if it is just more dependent on how quickly some analysts work versus others.***
    Our plan is that we will stop accepting responses exactly 8 months after the test starts. We assume that some participants will finish much faster than this.

14. ***If we get into a situation where our case work has to be prioritized and we have to stop, is that a foreseeable problem? (updated 4 April 2019)***
    We realize that not everyone will be able to complete the study: we will report results for participants who complete at least [30] comparisons (out of the 100 assigned).
    [Ed note: two versions of the FAQs were distributed accidentally creating a contradiction, with some stating "at least 20" and some stating "at least 30." The 20 threshold was used in other communications (e.g. Appendix 0), and was the final threshold we used.]

15. ***Are there strict deadlines for participating (such as exams completed within a day, a week, or by a certain date)? (updated 4 April 2019)***
    We will not have weekly or monthly deadlines. We will put in some limits so that examiners cannot (for example) do all 100 digital comparisons in a day, but that shouldn't affect your long-term planning.

16. ***How will the Physical Subtest be conducted? (added 4 April 2019)***

    Participants in the Handwriting Black Box Study may optionally participate in the "Physical Subtest," which will be conducted in person using originals, but otherwise follows the same procedure as the Digital Subtest. The Physical Subtest will be conducted at document examination conferences, and in the Washington DC area. Details and instructions are in progress, and will be sent to all registered participants.

17. ***Do we need to consider that the samples may include tracing or simulated writing? (added 4 April 2019)***

    No. To the best of our knowledge all samples included were freely and naturally prepared. (An early draft of the instructions included a question about this, but we are no longer including that as part of the test.

18. ***Are all the known samples in a comparison set definitely from the same writer? (When I indicate known writing specimens submitted from various writers in my daily casework I do so by labeling the writers as K1, K2, K3, etc. The labeling in this research may be different from mine. I just want to make sure writings K1 and K2 submitted for test 2 are submitted for one writer, given that there is not a choice of two writers in the answer sheet). (added 4 April 2019)***

    All K samples in a given comparison set are always from a single writer.

Appendix E Test Yield

Table S8 shows the counts of participants by the number of responses per participant. Of the 86 participants, 58 completed all 100 assigned comparisons, but after omitting 3 responses based on participants comments indicating a clerical error, 13 of the 58 ended up with 98 or 99 trials in the *Analysis Dataset*; 64 completed at least 90 of the assigned comparisons (all of whom had at least 90 trials in the *Analysis Dataset*). For the subset of analyses in which we compute and compare individual rates for each participant, we limit analyses to the 70 participants who completed at least half of the assigned comparisons (*Examiner Comparison Dataset)*.

		# Participants		*Usage*	
		Analysis Dataset (n=7,196)	*Original Responses (n=7,288)*		
# QKsets	*1-19*	0	20	Omitted from all analyses	
	20-29	5	5	Analysis Dataset	
	30-39	8	8		
	40-49	3	3		
	50-59	4	4		Examiner Comparison Dataset
	60-69	0	0		
	70-79	2	2		
	80-89	0	0		
	90-99	19	6		
	100	45	58		
		86	106		

Table S8. Counts of participants. A total of 86 participants are used for analyses (*Analysis Dataset*), of whom 70 participants are used in calculating rates for individual participants and comparisons of participants (*Examiner Comparison Dataset)*.

Table S9 summarizes the number of QKsets and responses used in this study. The study was based on a total of 180 distinct QKsets. Of these, 20 QKsets were assigned twice to the same participants (identical images, but different QKset number) in order to assess intra-examiner variation—these re-assignments are termed "2nd assignments" and are generally omitted from analyses, unless otherwise specified. Each participant was assigned 90 distinct QKsets (39 mated, 51 nonmated) and 10 repeated QKsets (5 mated, 5 nonmated) from the pool of 180 distinct QKsets/200 total QKsets.

Analyses are based on 7,196 responses from 86 participants (*Analysis Dataset*). Although 7,288 responses from 106 examiners were originally received, we omitted 92 trials and 20 examiners from all analyses:

- We omitted 72 responses from 20 participants who completed fewer than 20 trials each. The decision to omit responses from participants who completed less than 20 trials was made prior to the commencement of testing.
- We omitted 17 2nd assignment responses in which the 1st and 2nd assignments were inadvertently assigned consecutively: to test repeatability, the trials need to be assigned with intervening comparisons.
- We omitted 3 responses based upon participant comments indicating that there were clerical errors (e.g., a comment indicating the previous response was for the wrong QKset).

		All	*Mated*	*Nonmated*
QKsets	Distinct QKsets	180	78	102
	Repeated QKsets	20	10	10
	Total QKsets	200	88	112
Responses	Not repeated	5,939	2,550	3,389
	1st assignment	620	313	307
	2nd assignment	620	313	307
	Analysis Dataset	7,196	3,176	4,020
	Baseline Dataset — omits 2nd assignment	6,576	2,863	3,713

Table S9. Counts of QKsets and responses both overall and by mating (mated/nonmated).

Table S10 summarizes the datasets used for analyses. Most analyses are based on the *Baseline Dataset* (6,576 trials), which omits 2nd assignments. The *Repeatability Dataset* is used for analyses specific to measuring

repeatability. When comparing performance between participants and associating performance with background, the *Examiner Comparison Dataset* is used. Since these analyses require computation and comparison of individual error rates, this data is a subset of the *Baseline Dataset* containing the 6,096 trials from those participants who completed at least half of all assigned comparisons, thereby omitting participants with relatively few trials in an effort to produce more stable estimates.

The *Baseline Dataset* contained responses from 31-48 participants per distinct QKset (median 37, mean 36.5). Over all data (including 2nd assignments), responses were received from 31-81 participants per distinct QKset (median 37, mean 40.0).

Dataset	*Trials*	*Distinct QKsets*	*Repeated QKsets*	*Participants*	*Description*
Baseline Dataset	6,576	180	0	86	Default dataset for analyses — omits 620 2nd assignments
Analysis Dataset	7,196	180	20	86	Includes 2nd assignments
Repeatability Dataset	1,240	20	20	65	620 pairs of 1st & 2nd assignments
Examiner Comparison Dataset	6,096	180	0	70	Subset of Baseline Dataset for measuring rates for each examiner & for comparing examiners. Omits 16 participants without at least 50 QKsets each.
Subset of baseline omitting QA	6,435	176	0	86	Omits addresses (QA)
Subset of baseline limited to K1	2,363	64	0	86	Omits K3 and K5
Subset of baseline limited to QL	5,309	144	0	86	Omits QA and QS

Table S10. Datasets used in analyses.

Appendix F Conclusion rates

Appendix F1 Defining Terms

Although measures of accuracy, error rates, and predictive values are longstanding and used universally in a variety of fields, they were originally formulated and are generally applied to binary decision tasks with explicit "positive" and "negative" outcomes. However, implementation becomes more ambiguous for decision tasks involving more than two levels. In the forensic literature, there has been disagreement about how to handle "inconclusive" responses for three-level conclusion scales and whether they should be included in the denominator for computing accuracy and error rates (see the following sources for some examples/discussion: (9, 17–19)). However, in 2018 the OSAC Human Factors Committee proffered the following guidance for reporting such results for performance tests of forensic examiners using three-level conclusion scales (18):

> *Importantly, false positives and false negatives are reported three ways: (1) as a percentage of all presentations (% PRES); (2) as a percentage of all comparisons, i.e., excluding those comparisons where the impressions were deemed to be of no value (% COMP); and (3) as a percentage of all conclusive calls, i.e., excluding both no value comparisons and inconclusive (% CALLS). PCAST advocates reporting error rate data as a percentage of conclusive calls (ignoring no value and inconclusive comparison), on grounds that cases where examiners reached a conclusion are those likely to be used in a criminal proceeding, and hence the rates of error for those conclusions are most relevant. Our view is that forensic scientists should be prepared to present error rate data for their methods in a variety of ways.*

More recently, the OSAC Human Factors Committee expanded their guidance to include larger categorical scales (e.g., five or seven level scales) and recommended an analogous approach for computing accuracy, wherein %CALLS includes the additional noninconclusive categories (19). For this study, we have opted to implement and expand upon the recommendations outlined by the OSAC Human Factors Committee, and we present accuracy and error rates for our five-level conclusion scale as follows:

- %PRES: includes all presentations in the denominator (does not omit any trials)
- %CALLS: omits inconclusive calls in from the denominator (omits any trials resulting in *NoConc*)[†]

In order to report rates for qualified conclusions in this study we define the following rates:

- Correct positive association rate (CAR) — Proportion of mated QKset trials resulting in *ProbWritten*
- Incorrect positive association rate (IAR) — Proportion of nonmated QKset trials resulting in *ProbWritten*
- Correct negative association rate (CNR) — Proportion of nonmated QKset trials resulting in *ProbNot*
- Incorrect negative association rate (INR) — Proportion of mated QKset trials resulting in *ProbNot*

Appendix F2 Accuracy and Error Rates

Table S11 details the distribution of conclusion rates for the *Baseline Dataset*; see Figure 1 (in the main paper) for a graphical display of the PRES results.

[†] *The Latent Print Black Box Study* (9) *and OSAC Human Factors guidance document* (19) *also define "COMP" (comparisons), which is not relevant in this study, because every presentation of a trial resulted in a comparison (unlike studies in which there is a separate suitability/value assessment).*

	Mated			Nonmated			% Mated	% Nonmated
	#	%PRES	%CALLS	#	%PRES	%CALLS		
Written	1546	54.0%	59.0%	114	3.1%	3.6%	93.1%	6.9%
ProbWritten	982	34.3%	37.5%	179	4.8%	5.7%	84.6%	15.4%
NoConc	243	8.5%	---	547	14.7%	---	30.8%	69.2%
ProbNot	60	2.1%	2.3%	1774	47.8%	56.0%	3.3%	96.7%
NotWritten	32	1.1%	1.2%	1099	29.6%	34.7%	2.8%	97.2%
Total Presentations (PRES)	2863			3713			43.5%	56.5%
Total Calls (CALLS)	2620			3166			45.3%	54.7%
Total Definitives (DEF)	1578			1213			56.5%	43.5%

Table S11. Conclusion rates for the *Baseline Dataset* as a function of the total presentations ("PRES", all trials), and total calls ("CALLS", all trials not resulting in *NoConc*), (19). The % Mated and % Nonmated values are calculated for each row; these are the basis for the conditional probabilities discussed in *Appendix F3*.

Table S12, Table S13, and Table S14 provide a variety of metrics for assessing the accuracy of conclusions reached by participants in this study, with confidence intervals (CIs), all calculated from values shown in Table S11. CIs are reported using Clopper-Pearson, a commonly utilized binomial CI approach that produces conservative estimates of the interval (20). Note that since rates are not evenly distributed by QKset or by participant (heteroscedastic), any approach for measuring CIs is necessarily imperfect. Furthermore, the Clopper-Pearson estimate, like most other CI methods, assumes independence among decisions; because our data includes commonalities of examiners and image pairs, we expect the confidence intervals presented here may be somewhat narrower than appropriate for the data.

Metric	*Abbreviation*	*Definition*	*Rate*	*C.I.*	*Counts*
True positive rate (Sensitivity)	TPR	Proportion of mated QKset trials resulting in *Writtens*			
	TPR_{PRES}	(including all mated QKset trials in the denominator)	54.0%	[52.2%-55.8%]	(1546/2863)
	TPR_{CALLS}	(omitting mated QKset trials that resulted in *NoConc*)	59.0%	[57.1%-60.9%]	(1546/2620)
Correct positive association rate	CAR	Proportion of mated QKset trials resulting in *ProbWrittens*			
	CAR_{PRES}	(including all mated QKset trials in the denominator)	34.3%	[32.6%-36.1%]	(982/2863)
	CAR_{CALLS}	(omitting mated QKset trials that resulted in *NoConc*)	37.5%	[35.6%-39.4%]	(982/2620)
True positive + Correct positive association rate	TPR+CAR	Proportion of mated QKset trials resulting in *Writtens* or *ProbWrittens*			
	$TPR+CAR_{PRES}$	(including all mated QKset trials in the denominator)	88.3%	[87.1%-89.5%]	(2528/2863)
	$TPR+CAR_{CALLS}$	(omitting mated QKset trials that resulted in *NoConc*)	96.5%	[95.7%-97.2%]	(2528/2620)
True negative rate (Specificity)	TNR	Proportion of nonmated QKset trials resulting in *NotWrittens*			
	TNR_{PRES}	(including all nonmated QKset trials in the denominator)	29.6%	[28.1%-31.1%]	(1099/3713)
	TNR_{CALLS}	(omitting nonmated QKset trials that resulted in *NoConc*)	34.7%	[33.1%-36.4%]	(1099/3166)
Correct negative association rate	CNR	Proportion of nonmated QKset trials resulting in *ProbNots*			
	CNR_{PRES}	(including all nonmated QKset trials in the denominator)	47.8%	[46.2%-49.4%]	(1774/3713)
	CNR_{CALLS}	(omitting nonmated QKset trials that resulted in *NoConc*)	56.0%	[54.3%-57.8%]	(1774/3166)
True negative + Correct negative association rate	TNR+CNR	Proportion of nonmated QKset trials resulting in *NotWrittens* or *ProbNots*			
	$TNR+CNR_{PRES}$	(including all nonmated QKset trials in the denominator)	77.4%	[76.0%-78.7%]	(2873/3713)
	$TNR+CNR_{CALLS}$	(omitting nonmated QKset trials that resulted in *NoConc*)	90.7%	[89.7%-91.7%]	(2873/3166)

Table S12. Summary accuracy rates and Clopper-Pearson confidence intervals for conclusions consistent with ground truth. Numerators and denominators for each calculation are shown to avoid ambiguities.

Metric	*Abbreviation*	*Definition*	*Rate*	*C.I.*	*Counts*
False positive rate	FPR	Proportion of nonmated QKset trials resulting in *Writtens*			
	FPR_{PRES}	(including all nonmated QKset trials in the denominator)	3.1%	[2.5%-3.7%]	(114/3713)
	FPR_{CALLS}	(omitting nonmated QKset trials that resulted in *NoConc*)	3.6%	[3.0%-4.3%]	(114/3166)
Incorrect positive association rate	IAR	Proportion of nonmated QKset trials resulting in *ProbWrittens*			
	IAR_{PRES}	(including all nonmated QKset trials in the denominator)	4.8%	[4.2%-5.6%]	(179/3713)
	IAR_{CALLS}	(omitting nonmated QKset trials that resulted in *NoConc*)	5.7%	[4.9%-6.5%]	(179/3166)
False positive + Incorrect positive association rate	FPR+IAR	Proportion of nonmated QKset trials resulting in *Writtens* or *ProbWrittens*			
	$FPR+IAR_{PRES}$	(including all nonmated QKset trials in the denominator)	7.9%	[7.0%-8.8%]	(293/3713)
	$FPR+IAR_{CALLS}$	(omitting nonmated QKset trials that resulted in *NoConc*)	9.3%	[8.3%-10.3%]	(293/3166)
False negative rate	FNR	Proportion of mated QKset trials resulting in *NotWrittens*			
	FNR_{PRES}	(including all mated QKset trials in the denominator)	1.1%	[0.8%-1.6%]	(32/2863)
	FNR_{CALLS}	(omitting mated QKset trials that resulted in *NoConc*)	1.2%	[0.8%-1.7%]	(32/2620)
Incorrect negative association rate	INR	Proportion of mated QKset trials resulting in *NotWrittens*			
	INR_{PRES}	(including all mated QKset trials in the denominator)	2.1%	[1.6%-2.7%]	(60/2863)
	INR_{CALLS}	(omitting mated QKset trials that resulted in *NoConc*)	2.3%	[1.8%-2.9%]	(60/2620)
False negative + incorrect negative association rate	FNR+INR	Proportion of mated QKset trials resulting in *NotWrittens* or *ProbNots*			
	$FNR+INR_{PRES}$	(including all mated QKset trials in the denominator)	3.2%	[2.6%-3.9%]	(92/2863)
	$FNR+INR_{CALLS}$	(omitting mated QKset trials that resulted in *NoConc*)	3.5%	[2.8%-4.3%]	(92/2620)

Table S13. Summary error rates and Clopper-Pearson confidence intervals for conclusions that contradict ground truth. Numerators and denominators for each calculation are shown to avoid ambiguities.

Appendix F3 Conditional probabilities

Although error rates are important measures for characterizing the accuracy of forensic document examiners, they require *a priori* knowledge of the ground truth mate relationship. In casework, this information is rarely, if ever, known; instead, the quantity of interest becomes the likelihood that a given conclusion is correct, which can be computed using conditional probabilities (PPV and NPV as defined in Table S14). These conditional probabilities modify the error rates by the mate prevalence (the proportion of all trials that were mated- see (9) for a more detailed discussion and necessary equations) in order to determine the chance that a conclusion is correct (e.g., the chance that a decision of *Written* was reported on a mated trial). Fig S3 plots PPV and NPV across a range of mate prevalence (from 0% to 100%) since this proportion can vary between laboratories and depending upon the case factors. A 50% mate prevalence is a useful point of comparison because it assumes uninformative priors, but it may or may not be relevant to any given operational scenario.

Metric	*Rate*	*C.I.*	*Counts*	*Rescaled to 50:50*
Positive predictive value (PPV) — Proportion of Writtens that were correct (i.e. on mated QKsets)	93.1%	[91.8%-94.3%]	(1546/1660)	94.6%
False discovery rate (1-PPV) — Proportion of Writtens that were incorrect (i.e. on nonmated QKsets)	6.9%	[5.7%-8.2%]	(114/1660)	5.4%
Negative predictive value (NPV) — Proportion of NotWrittens that were correct (i.e. on nonmated QKsets)	97.2%	[96.0%-98.1%]	(1099/1131)	96.4%
False omission rate (1-NPV) — Proportion of NotWrittens that were incorrect (i.e. on mated QKsets)	2.8%	[1.9%-4.0%]	(32/1131)	3.6%
Proportion of ProbWrittens that were correct (i.e. on mated Qksets)	84.6%	[82.4%-86.6%]	(982/1161)	87.7%
Proportion of Writtens and ProbWrittens that were correct (i.e. on mated Qksets)	89.6%	[88.4%-90.7%]	(2528/2821)	91.8%
Proportion of ProbNots that were correct (i.e., on nonmated QKsets)	96.7%	[95.8%-97.5%]	(1774/1834)	95.8%
Proportion of NotWrittens and ProbNots that were correct (i.e., on nonmated QKsets)	96.9%	[96.2%-97.5%]	(2873/2965)	96.0%

Table S14. Conditional probabilities of accuracy and error for the *Baseline Dataset* (43.5% mate prevalence), and rescaled to 50:50 mate prevalence. All rates calculated from counts shown in Table S11.

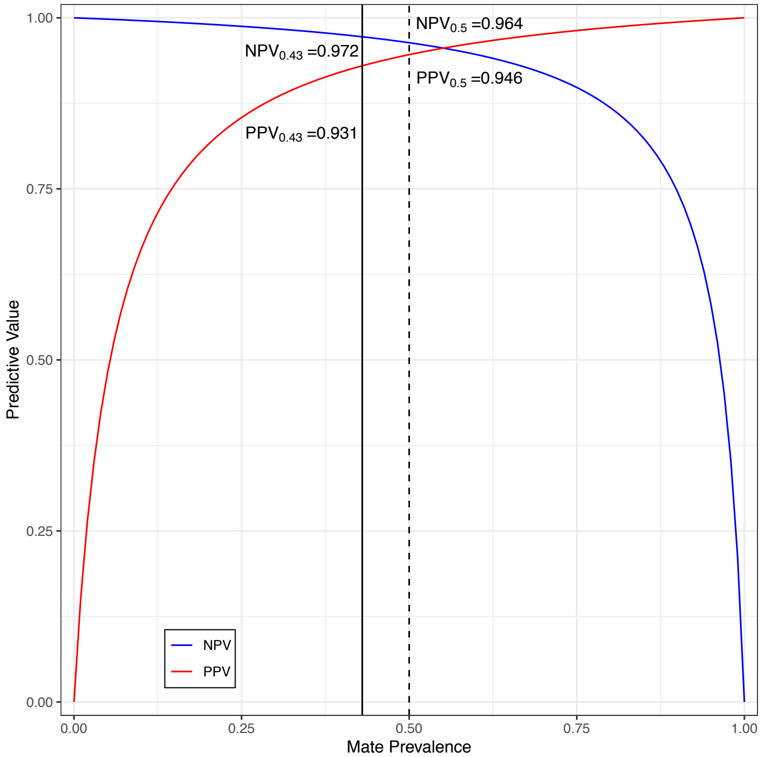

Fig S3. Positive (red) and negative (blue) predictive values estimated as a function of mate prevalence. The solid line indicates the mate prevalence for the *Baseline Dataset* (43.5% mated trials); the dashed line indicates a mate prevalence of 50.0%.

Appendix F4 False positives (FPs)—Erroneous "Written By" conclusions

Participants in this study erroneously reported *Written* 114 times across all 3,713 nonmated trials in the *Baseline Dataset*, yielding a false positive rate (FPR_{PRES}) of 3.1%. An additional 17 FPs were reported in response to the 2nd assignments in the *Repeatability Dataset*: see Appendix F7 for details for the repeatability data.

The false positives were not limited to a few individuals: 42 of the 86 participants made at least one FP conclusion for the *Baseline Dataset*. However, some individuals did have higher quantities of FPs; in fact, 53.5% of all erroneous *Written* conclusions were reported by just under 10% of the participants in the study (8 participants made 5-12 such errors each, three of whom reported 10+ such errors). All three of the participants who committed 10+ FPs have at least 5 years of experience, did not complete 2+ years of formal training, did not testify as a handwriting expert during training, conduct handwriting examinations daily, completed a proficiency test in the last year, and sometimes limit their conclusions when comparisons entail only photocopies or photographs; two of them hold at least a Bachelor's degree. A total of six participants meets these criteria—note the others who shared these criteria did not show a similarly problematic performance.

Similarly, the erroneous *Written* conclusions were not limited to a few QKsets: 55 of the 102 nonmated QKsets resulted in at least one FP error for the *Baseline Dataset* (Appendix F7 provides additional details for the repeat data). However, some specific QKsets resulted in a disproportionately high number of FPs; in particular, a single QKset (QK472) accounted for 13 FPs in the *Baseline Dataset* (and ten in the 2nd assignments in the *Repeatability Dataset*). Figure 3 (main paper) displays QKset 472, a nonmated comparison with questioned and known writing samples obtained from twins. Although trials on twins data comprise 9.9% of all nonmated trials, 28.1% of all erroneous *Written* decisions were rendered on these QKsets (Table S15). Moreover, participants were 3.7 times more likely to report a FP error on twin trials versus nontwin trials (based upon the Odds Ratio). Based upon a bootstrapped difference of proportions analysis, the FPRs between twin and nontwin trials were significantly different ($p < 0.0001$)—FP errors are disproportionately more prevalent in twins data.

	Twins	*Nontwins*	*Total*
# nonmated trials	368	3,345	3,713
# *Written* (FPs)	32	82	114
FPR	8.7%	2.5%	3.1%

Table S15. False positive errors for nonmated QKsets containing writing from twins versus nontwins. (*Baseline Dataset*)

As previously discussed, the handwriting of twins has been shown to be highly similar, although still differentiable, through research (2, 4–6). With respect to QK472 (Figure 3 (main paper)), numerous similarities are observed in style, size, slant, class characteristics and even individual characteristics. However, both class and repeated individual characteristics are present that are not in common and should not be ignored or explained away as variation. Presumably, such dissimilarities were observed by at least 11 participants who reported *ProbNot or NotWritten* for the baseline data; an additional 7 participants reported *NoConc,* suggesting that they may have observed some of these inconsistencies but were unsure about whether they were true differences or part of the natural variation in writing (particularly given the limited quantity of known writing). It should be noted that the questioned document sample shown in Figure 3 (main paper) was cropped to remove some parts of the CEDAR-FOX letter that contained notable differences in formations between the writers and may have aided in reaching a nonassociation decision. Nevertheless, it is important to acknowledge that this type of large volume, time-limited comparison test with nonoriginal documents, relatively limited quantities of known writing especially in the case of similar writers, and no technical review or consultation allowed could increase the likelihood of errors in comparison to how casework is normally performed.

For each QKset examined, participants were also asked to rate the perceived difficulty of the comparison as it was completed. The distribution of perceived difficulty for all nonmated trials in the *Baseline Dataset* is shown in Table S16. Interestingly, there were significant differences in difficulty ratings as a function of conclusion outcome (FP vs not FP) based upon a chi-square analysis with Bonferroni-adjusted standardized residual post-hoc ($p < 0.001$). In trials that resulted in an erroneous *Written* conclusion, participants were much more likely than expected to rate the comparison as Easy or Very Easy; conversely, for trials that did not result in a FP, participants were much more likely to rate the comparison as Difficult. These results seem to suggest that the difficulty ratings rendered by participants are a reflection of how hard it was to make a decision and are likely a function of perceived similarity in writing, comparability of content, and degree of expected/observed variation. Since eliminating writers is a particularly difficult task, especially when comparing highly similar, nonmated writers such as twins, when given a limited quantity of known writing, participants who did not report a false positive error (i.e., did not report *Written* and instead reported a *NotWritten* or a less confident qualified opinion or indeterminate decision) would presumably be more likely to perceive the comparison as more challenging/less easy, as we observed in these results.

	FP		*NonFP*		*Total*	
VDiff	4	4%	182	5%	186	5%
Diff	5	4%	879	24%	884	25%
Med	45	39%	1,665	46%	1,710	48%
Easy	46	40%	787	22%	833	23%
VEasy	14	12%	86	2%	100	3%
Total	114		3,599		3,713	

Table S16. QKset difficulty ratings for false positive vs not false positive conclusions. (Baseline Dataset)

Appendix F5 Challenges in eliminating writers ("Not Written By" conclusions)

A review of the accuracy and error rates reveals that unlike other pattern fields (e.g., fingerprints or footwear), the false negative rates for handwriting examination are lower than the false positive rates; similarly, the true negative rate is lower than the true positive rate (Table S12 and Table S13). These measures reflect the difficulty in eliminating writers, especially with a limited quantity of known writing in these sample sets, which ultimately results in a smaller quantity of *NotWritten* decisions and accordingly lower rates for this category. A critical difficulty is that FDEs must determine that the known writer could not have written the questioned handwriting

under any circumstances. This includes accounting for intentional or accidental distortion, the possibility of more than one writing style, writing position, drugs or any other transitory or permanent factors. As previously discussed, handwriting is a learned neuromuscular process that entails wider intra-subject variation than for disciplines based on physical characteristics (such as fingerprints) or manufactured items (such as firearms or footwear), a variation that examiners must properly characterize and account for during comparisons. Inherent in handwriting comparisons is the principle that no one writes exactly the same way twice, but that there is a pattern of repeated characteristics that can be observed given sufficient quantity and quality of writing. When a small amount of known writing is provided for a comparison, it presents only a limited number of examples of a character or combination of characters, thereby presenting a subset of the full range of the writers' capability, range of variation, and styles of writing. In addition, depending on the content of the known writing, it may or may not be comparable to the questioned writing. Without demonstration of the writer's entire range of writing habits, elimination of a writer may not be feasible.

Additional challenges in eliminating writers include the possibility of multiple writing styles, influence of questioned/known writing by external factors (environment, substrate, writing instrument) or internal factors (intent, drugs, alcohol, injury), and change of handwriting over time either due to progressive development of skill, deterioration of motor skills, or by design. The ideal set of known writing samples for a handwriting comparison includes a large quantity of writing that is both comparable and contemporaneous and may come from normal course of business as well as dictations. However, an insufficient quantity of comparable known writing is one of the most common deficiencies faced by FDEs.

Appendix F6 False negatives (FNs) —Erroneous "Not Written By" conclusions

Overall, participants in this study erroneously reported *NotWritten* 32 times across all 2,863 mated trials in the *Baseline Dataset*, resulting in a false negative rate (FNR_{PRES}) of 1.1%. Table S17 details the number of false negatives per participant and per distinct QKset in the *Baseline Dataset*.

#FNs	*# of participants* Baseline dataset	#FNs	*# of QKsets* Baseline dataset
0	69	0	56
1	11	1	15
2	2	2	4
3	2	3	3
4	1		
7	1		

Table S17. Number of false negatives by participant (86 total) and distinct mated QKset (78 total).

The majority (80.2%) of participants did not report an erroneous *NotWritten* conclusion, and another 12.8% of participants only rendered a single FN decision. The remaining 6 participants reported between two and seven FN errors. Note that the participant who reported the highest number of FNs (7) also reported the third highest number of FPs (10) in the *Baseline Dataset*.

Similarly, the majority of mated QKsets (71.8%) did not yield any FNs, and another 19.2% of QKsets only resulted in a single erroneous *NotWritten* conclusion. The remaining 7 QKsets yielded 2 or 3 FN errors each. Fig S4 illustrates QK554, which accounted for 3 false *NotWritten* conclusions as well as 11 incorrect *ProbNot* decisions, the highest number for the *Baseline Dataset*. Although the questioned sample and known sample were written by the same individual, there are apparent differences in slant and spacing; furthermore, the content comparability between the two documents is minimal, thus limiting the comparison to single words and/or letter combinations. This example illustrates the difficulties of working with limited quantity of known writing and limited comparability of known writing, circumstances that can preclude a FDE from being able to adequately characterize and account for the degree of variation in an individual's writing during a comparison. These results tend to reemphasize the importance of obtaining sufficient quality and quantity of comparable known writing to minimize the potential for errors and facilitate an effective examination.

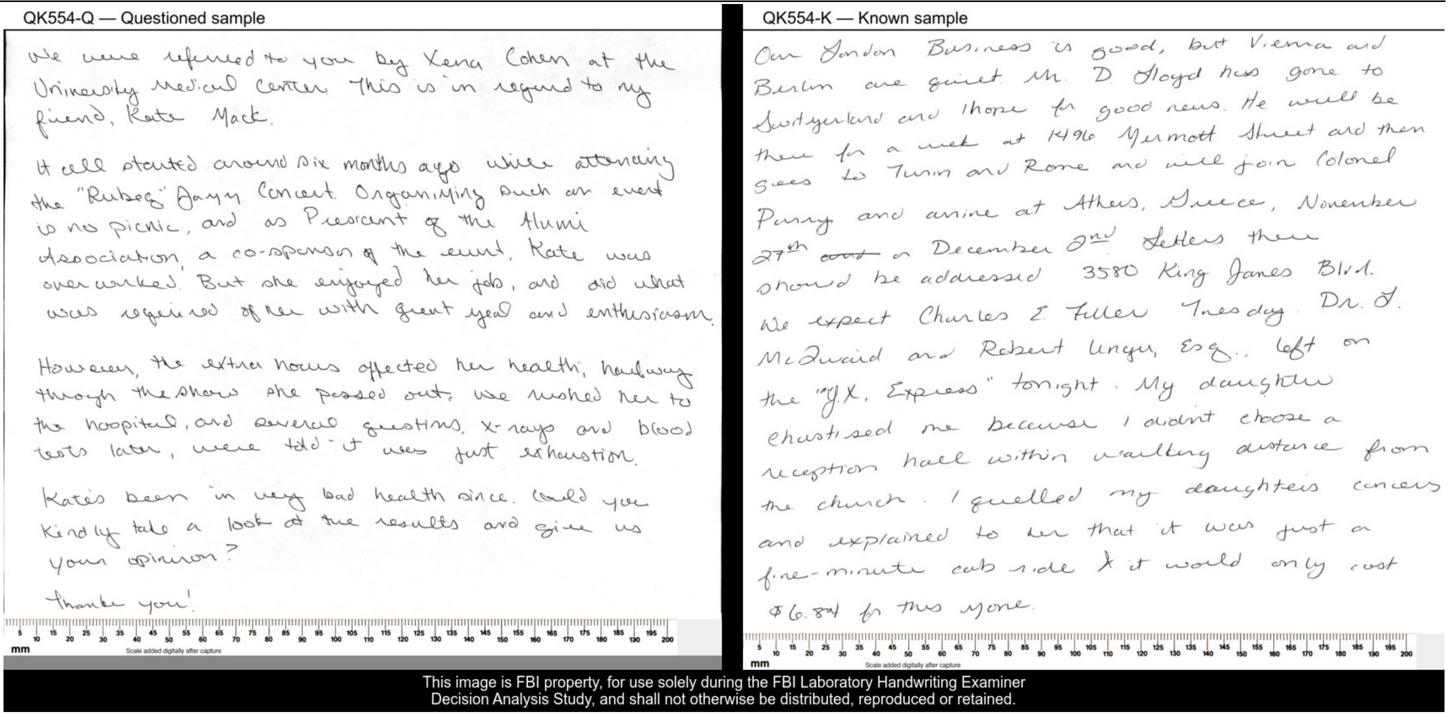

Fig S4. QK554: mated QKset that resulted in 3 FPs in the *Baseline Dataset* (and an additional ten FPs in the 2nd assignments) and also had the highest number of INs in the *Baseline Dataset*. Note the lack of content comparability as well as the differences in slant and spacing. Conclusion rates for this QKset: *Written*: 3 in baseline data; *ProbWritten*: ten in baseline data; *NoConc*: 14 in baseline data; *ProbNot*: 11 in baseline data; *NotWritten*: 3 in baseline data. Note that this QKset was not a included in the *Repeatability Dataset*.

The distribution of perceived difficulty for all mated trials in the *Baseline Dataset* is shown in Table S18. Unlike the results for FP errors, we did not detect differences in difficulty ratings as a function of conclusion outcome (FN vs not FN) based upon a chi-square analysis ($p = 0.227$).

	FN		*NonFN*		*Total*	
VDiff	1	3%	82	3%	83	3%
Diff	6	19%	394	14%	400	14%
Med	17	53%	1078	38%	1095	38%
Easy	7	22%	953	34%	960	34%
VEasy	1	3%	324	11%	325	11%
Total	32		2831		2863	

Table S18. QKset difficulty ratings for false negative vs not false negative conclusions. (*Baseline Dataset*)

Appendix F7 Repeatability of Errors and Incorrect Responses

Table S19 shows the repeatability of errors and incorrect responses. For example, there were 20 FPs in the 1st responses (i.e., *Written* responses on nonmated QKsets), of which eight were repeated in the 2nd responses. There were four FNs in the 1st responses, of which none were repeated.

The eight repeated FPs were made by six participants. One participant (shown as a blue diamond in Figure 4 in the main paper) made three repeated FPs, in addition to 12 FPs in the *Baseline Dataset*.

The eight repeated FPs were made on four QKsets, three of which were twins. Five of the eight repeated FPs were on the (twins) QKset shown in Figure 3 (main paper), which received 13 FPs in the *Baseline Dataset* and ten in 2nd assignments.

1st Response	*2nd Response*	*Mates*	*Nonmates*
Written	Written	170	8
Written	ProbWritten	34	3
Written	NoConc	0	2
Written	ProbNot	0	4
Written	NotWritten	1	3
ProbWritten	Written	11	4
ProbWritten	ProbWritten	58	12
ProbWritten	NoConc	7	10
ProbWritten	ProbNot	4	12
ProbWritten	NotWritten	0	5
NoConc	Written	3	1
NoConc	ProbWritten	5	2
NoConc	NoConc	6	15
NoConc	ProbNot	3	20
NoConc	NotWritten	0	1
ProbNot	Written	1	2
ProbNot	ProbWritten	2	0
ProbNot	NoConc	2	14
ProbNot	ProbNot	1	87
ProbNot	NotWritten	1	15
NotWritten	Written	0	2
NotWritten	ProbWritten	3	2
NotWritten	NoConc	1	3
NotWritten	ProbNot	0	13
NotWritten	NotWritten	0	67
Total		313	307

Table S19. Repeatability of errors and incorrect responses. Responses in which both the 1st and 2nd responses were contrary to ground truth are shown in orange; responses in which either the 1st and 2nd responses were contrary to ground truth are shown in yellow. (*Repeatability Dataset*)

Appendix G Effect of quantity and comparability of writing

Appendix G1 Analysis of pairs of factors assessing quantity and comparability

This study was designed to include an evaluation of whether any sample-related factors impacted the accuracy of the decisions reported by participating FDEs. This was achieved using 32 pairs of QKsets ("factor pairs"), totaling 64 of the 180 distinct QKsets. One QKset from each factor pair was presented to each participant who completed the study to isolate one variable. Each participant received one QKset from each factor pair, totaling 32 of the 90 distinct QKsets. Table S20 details the conclusion rates as a function of these factor pairs. In order to evaluate for differences in reporting between conditions, each pair of columns (sub-tables) was compared using a Bonferroni-adjusted Fisher Exact Test, which is an alternative approach to chi-square testing for small sample sizes (21).

- Abbreviations:
  - QS — Q Short — 1/3 London Letter (1/3 paragraph) or equivalent length
  - QL — Q Long — London Letter or equivalent length
  - K1 — K 1 page — 1 London Letter or equivalent length
  - K3 — K 3 pages — 3 London Letters or equivalent length
  - K5 — K 5 pages — 5 London Letters or equivalent length
  - D — Different content
  - S — Same content (e.g. the Q and at least 1 K are London Letters)
- For example, QK521-QK640 was a "QS-K3-x" factor pair of QKsets:
  - QK521 and QK640 both had QS & K3
  - Half the participants got QK521, which had different content (the Ks were free text)
  - Half the participants got QK640, which had same content (at least 1 K was identical content to the Q)

MATED	*Qx-K3-D*		*Qx-K3-S*		*QS-Kx-D*		*QS-Kx-S*		*QL-Kx-D*		*QL-Kx-S*		*QS-K3-x*		*QL-K3-x*	
	QS	*QL*	*QS*	*QL*	*K1*	*K5*	*K1*	*K5*	*K1*	*K5*	*K1*	*K5*	*Diff*	*Same*	*Diff*	*Same*
Written	42%	53%	55%	70%	33%	39%	50%	62%	18%	36%	58%	64%	43%	75%	38%	46%
ProbWritten	42%	42%	40%	26%	46%	39%	40%	33%	35%	46%	31%	28%	33%	25%	38%	35%
Written OR ProbWritten	85%	94%	95%	96%	79%	79%	90%	96%	53%	82%	89%	92%	76%	100%	75%	81%
NoConc	14%	6%	1%	3%	19%	18%	9%	3%	27%	7%	8%	4%	17%	0%	18%	11%
ProbNot OR NotWritten	1%	0%	4%	1%	1%	3%	1%	1%	19%	11%	3%	4%	7%	0%	6%	8%
ProbNot	0%	0%	0%	0%	1%	1%	1%	0%	16%	7%	1%	1%	4%	0%	6%	5%
NotWritten	1%	0%	4%	1%	0%	1%	0%	1%	4%	4%	1%	3%	3%	0%	0%	3%
QKsets	2	2	2	2	2	2	2	2	2	2	2	2	2	2	2	2
Responses	71	72	73	77	72	71	70	69	77	72	74	76	70	72	77	80
NONMATED	*Qx-K3-D*		*Qx-K3-S*		*QS-Kx-D*		*QS-Kx-S*		*QL-Kx-D*		*QL-Kx-S*		*QS-K3-x*		*QL-K3-x*	
	QS	*QL*	*QS*	*QL*	*K1*	*K5*	*K1*	*K5*	*K1*	*K5*	*K1*	*K5*	*Diff*	*Same*	*Diff*	*Same*
Written	6%	8%	3%	3%	3%	1%	0%	1%	0%	3%	0%	3%	1%	0%	3%	2%
ProbWritten	6%	1%	6%	3%	1%	0%	3%	9%	7%	5%	3%	6%	1%	3%	4%	2%
Written OR ProbWritten	12%	9%	9%	6%	4%	1%	3%	10%	7%	8%	3%	8%	3%	3%	7%	3%
NoConc	29%	18%	9%	6%	18%	20%	35%	18%	21%	8%	15%	13%	30%	19%	22%	14%
ProbNot OR NotWritten	59%	73%	83%	88%	78%	79%	63%	72%	72%	84%	83%	79%	67%	79%	71%	83%
ProbNot	45%	51%	49%	51%	53%	51%	44%	49%	46%	57%	45%	47%	46%	50%	46%	50%
NotWritten	14%	22%	34%	38%	25%	27%	18%	24%	25%	27%	38%	32%	21%	29%	25%	33%
QKsets	2	2	2	2	2	2	2	2	2	2	2	2	2	2	2	2
Responses	69	74	70	69	72	70	72	68	71	77	80	72	67	70	68	66

Table S20. Effects of quantity or content of writing. Each pair of columns shows the proportion of conclusions by a single factor: quantity of questioned writing (QS vs QL), quantity of known writing (K1 vs K5), or different or same content. Blue shading indicates instances where there was notable support of a difference according to a Bonferroni-adjusted Fisher Exact Test (α=0.05) (21). Note that Bonferroni-adjustments were made within each sub-table separately (e.g., MATED, Qx-K3-D at the top left). Yellow shading indicates instances where there was limited support of a difference according to a Bonferroni-adjusted Fisher Exact Test (α=0.15). (Subset of *Baseline Dataset*: 1,173 responses on 32 mated QKsets; 1,134 responses on 32 nonmated QKsets).

Appendix G1.1 Major takeaways – general

For the majority of comparisons, we did not detect significant differences in conclusion rates between the sample factors. However, statistically significant effects in reporting specific conclusions were observed with respect to an increase in the quantity of known writing as well as an increase in comparability, in some cases. For mated trials with a small quantity of questioned writing (MATED, QS-K3-x in Table S20), there was notable improvement in both definitiveness and accuracy when comparability increased. In addition, for mated trials with a larger quantity of questioned writing and different content (MATED, QL-Kx-D in Table S20), an increase in the quantity of known writing resulted in a notable improvement of definitiveness and limited improvement in accuracy. There was some limited statistical support that greater definitiveness was observed for nonmated pairs when the quantity of known writing increased for some specific scenarios, outlined in Table S20.

In addition, we observed several trends in the factor pair data—defined here as a difference of at least 8% for a given conclusion; we will discuss these trends in the following sections. Importantly, note that these observations were not statistically significant, possibly due to low power as a function of the small sample sizes (n=67-80); however, given this, we cannot preclude the possibility that these trends arose from chance alone.

Appendix G1.2 Effect of quantity of questioned writing

Although statistically significant differences between conclusion rates were not detected as a function of the quantity of questioned writing, possibly due to the small sample size, some trends were observed that are worth noting.

In general, when the quantity of questioned writing increased, the accuracy of conclusions likewise increased. More specifically, an increase in the TPR is observed when the quantity of questioned writing increases, regardless of whether the content is the same (+15%) or different (+11%). For mated trials, a change in the length of the Q with the same content resulted in relatively stable *NoConc* decisions and the increase in accuracy resulted from an increase in definitiveness. In contrast, for mated trials with different content, a change in the Q length resulted in a decrease of *NoConc* opinions with longer questioned writing (-8%), and as a corollary the collective percentage of TP+CA increased also (+9%), although this increase can be attributed to the increased proportion of *Written* conclusions.

A similar trend is observed for the TNR for the nonmated trials when the content is different (+8%). For nonmated trials with different content, *NoConc* opinions likewise trend down (-11%) when the quantity of questioned writing increased, yielding an increase in the collective percentage of TN+CN (+14%) for nonmated pairs with different content. Interestingly, the conclusion rates remain relatively consistent for nonmated trials with same content; this suggests that increased questioned writing length has a negligible impact on definitiveness and accuracy when the content is comparable between the questioned and known documents.

Appendix G1.3 Effect of quantity of known writing

Statistically significant differences in conclusion rates were detected as a result of increased known writing in the mated trials that contained a longer questioned document and known writing of different content. In particular, the collective TP+CA rates increased notably (+29%) and the proportion of *NoConc* decisions decreased notably (-20%) when the quantity of known writing increased from 1 to 5 pages. The TPR also exhibited limited support of a difference, with an 18% increase in accuracy with more known writing. Although not statistically significant, a similar trend was seen in the TPR when the questioned writing was short, and the content was the same (+12%).

With respect to nonmated trials, limited support for differences in definitiveness was detected in some scenarios. The *NoConc* rates were somewhat lower when additional known writing was provided in two cases: short questioned writing, same content (-17%) and long questioned writing, different content (-13%). Although not statistically significant, collective TN+CN rates also trended upwards for these factor pairs as quantity of known writing increased, likely due to the decreased inconclusive rates (QS-x-S: +9% and QL-x-D: +12%).

Appendix G1.4 Effect of same/different content

Comparability of known writing submitted for examination is of paramount importance to a forensic handwriting examination. Comparability may range from the exact same words to different content containing a sparse sampling of single characters. The level of comparability may impact the definitiveness of a conclusion and, if limited, could require a larger quantity of known writing to achieve a sufficiently comparable sample in order to be definitive.

In this study, we observed that for mated trials containing short questioned writing, the collective FN+IN rate decreased notably (-7%), the *NoConc* opinions decreased notably (-17%), the collective TP+CA rates increased notably (+24%) and the TPR increased notably (+32%) when presented with the same writing content as opposed to different content. Although not statistically significant, a positive trend was also observed in the TPR when the questioned writing was long and the same content was provided for known writing (+8%). These results suggest that comparability of content is immensely important, particularly when the length of the questioned writing is limited.

We did not detect any significant differences or interesting trends for nonmated trials with respect to writing comparability.

Appendix G2 Addresses

Sometimes in casework, questioned writing is extremely limited and only contains addresses (e.g., on a piece(s) of mail). Although it is possible to render a qualified opinion (*ProbWritten* or *ProbNot*) or even a definitive conclusion (*Written* or *NotWritten*) in this scenario, FDEs must be extremely cautious in doing so and ensure that they have sufficient quantity and quality of characteristics in the questioned writing to prevent reporting an erroneous or incorrect conclusion. Fig S5 illustrates conclusion rates when the questioned writing is an address—for mated and nonmated QKsets as well as same/different content. Not surprisingly, over half of decisions reported for QKsets presenting addresses with different content were *NoConc* (58% of mated trials; 64% of nonmated trials); further, there were very few definitive conclusions reported in this scenario (6% of mated trials; 3% of nonmated trials). However, when comparing addresses with the same content, the proportion of indeterminate decisions decreased (18% of mated trials; 16% of nonmated trials) and definitive conclusion rates increased (32% of mated trials; 34% of nonmated trials, which includes 3% FPs).

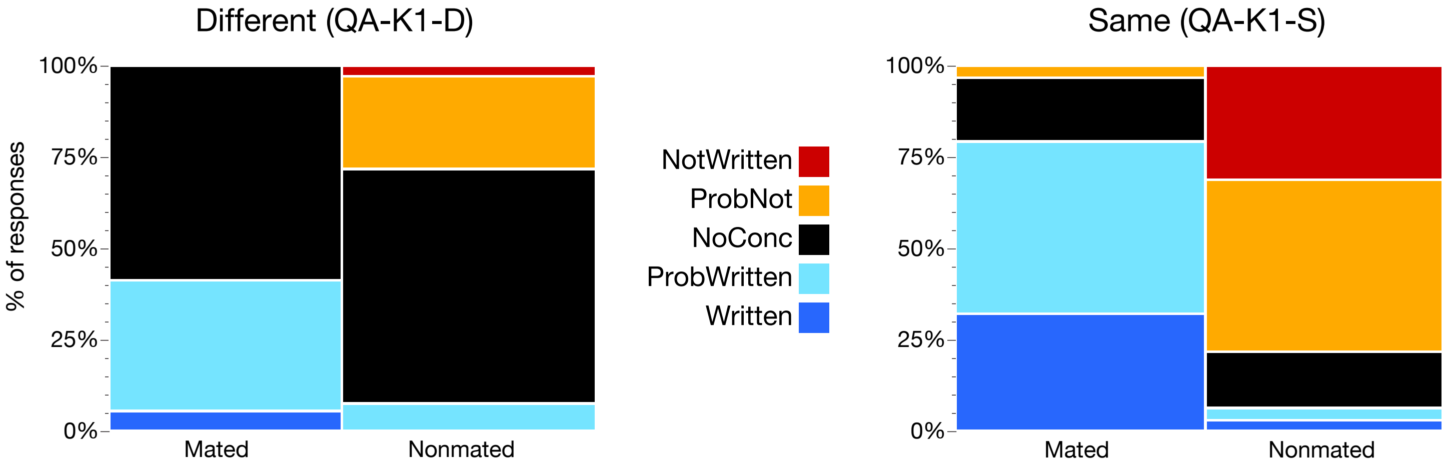

Fig S5. Conclusion rates where the Q is an address. (Subset of *Baseline Dataset*. QA-K1-D: 75 trials (36 mated, 39 nonmated). QA-K1-S: 66 trials (34 mated, 32 nonmated))

Appendix G3 Questioned and known writing amount and content comparability

Each QKset in the study, excluding addresses, was assigned into one of 12 comparison type categories describing its questioned writing length (Q length), number of known writing pages (K length), and content comparability (same/different) in order to allow for an assessment of the study-wide conclusion rates across these categories. Fig S6 displays the conclusions as a function of comparison type category for mated and nonmated trials; note

that the factor pair trials are also included in this data. These charts start with the most ideal comparison type for this study at the top (a long piece of questioned writing and 5 pages of known writing, both containing the same content) and move towards less ideal comparison circumstances (bottoming out at a short piece of questioned writing and 1 page of known writing, each containing different content).

Conclusions on mated trials were associated with comparison type—in general, as comparisons became less ideal, there were fewer *Written* conclusions and more *ProbWritten* conclusions. With respect to indeterminate decisions, comparisons with at least 3 pages of known writing containing the same content as the questioned sample had the lowest proportions of *NoConc*, while comparisons containing different content coupled with either short Q length or long Q length, but limited K length yielded the highest proportion of *NoConc*.

These trends were not nearly as striking for nonmated comparisons. However, the comparison type that resulted in the highest proportion of FP+IA conclusions was "Same-QL-K1". Of the 16 QKsets that comprise this category, 14 came from the Twins dataset, including QK472 (see Figure 3 (main paper)), which accounts for 34.0% (36/106) of the *Written* and *ProbWritten* decisions for this category.

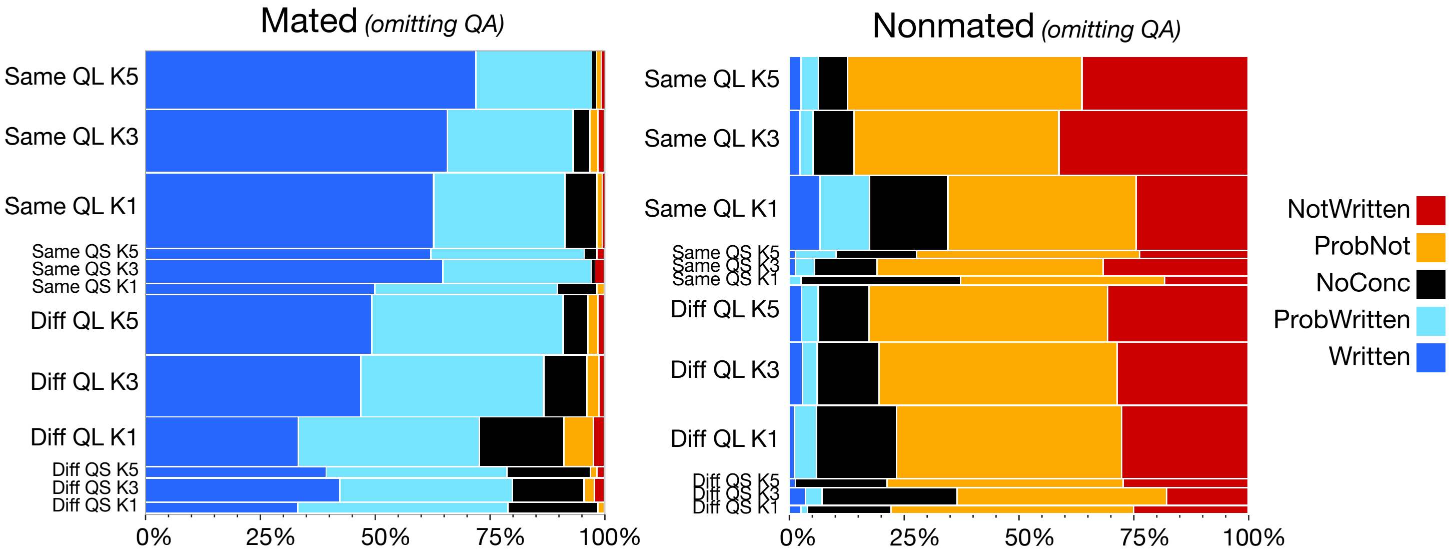

Fig S6. Conclusions by Q&K amount and same/different content. (Subset of *Baseline Dataset*, omitting 141 QA trials on 4 QA QKsets. Mated: 2,793 trials on 76 QKsets; nonmated: 3,642 trials on 100 QKsets)

To further investigate the interplay between these comparison sample factors, Fig S7 examines the impact of varying Q length and content on conclusions, while holding K length constant at 1 page (K1) — and examines the impact of varying K length and content on conclusions, while holding Q length constant at long (QL). Across both assessments, we did not observe any notable associations with errors on mated or nonmated trials.

Particularly for mated trials, the length of questioned writing and content comparability exhibit the expected trends with respect to definitiveness of conclusions: increased Q length generally yields an increase in the proportion of definitive conclusions. For mated trials, as writing content becomes more comparable (same as opposed to different), definitiveness likewise increases.

With respect to nonmated trials, these Q length trends were generally not observed (with the exception of addresses containing different content). In fact, TN+CN rates actually tended to be somewhat lower for comparisons involving short or long questioned writing (QS, QL) that contained the same content as the known writing relative to those containing different content. Conversely, TN+CN rates tended to generally increase with increased length of known writing and definitiveness was higher for the same content than for different content for the longer K's.

When only provided with a single page of known writing, TN+CN rates were somewhat lower for same content trials than different content trials. Conversely, TN+CN rates tended to generally increase with increased length

of known writing and definitiveness was higher for the same content than for different content for the longer K's. Overall, the results tend to reemphasize both the difficulty of excluding writers and the importance of obtaining sufficient known writing in terms of both content as well as quantity.

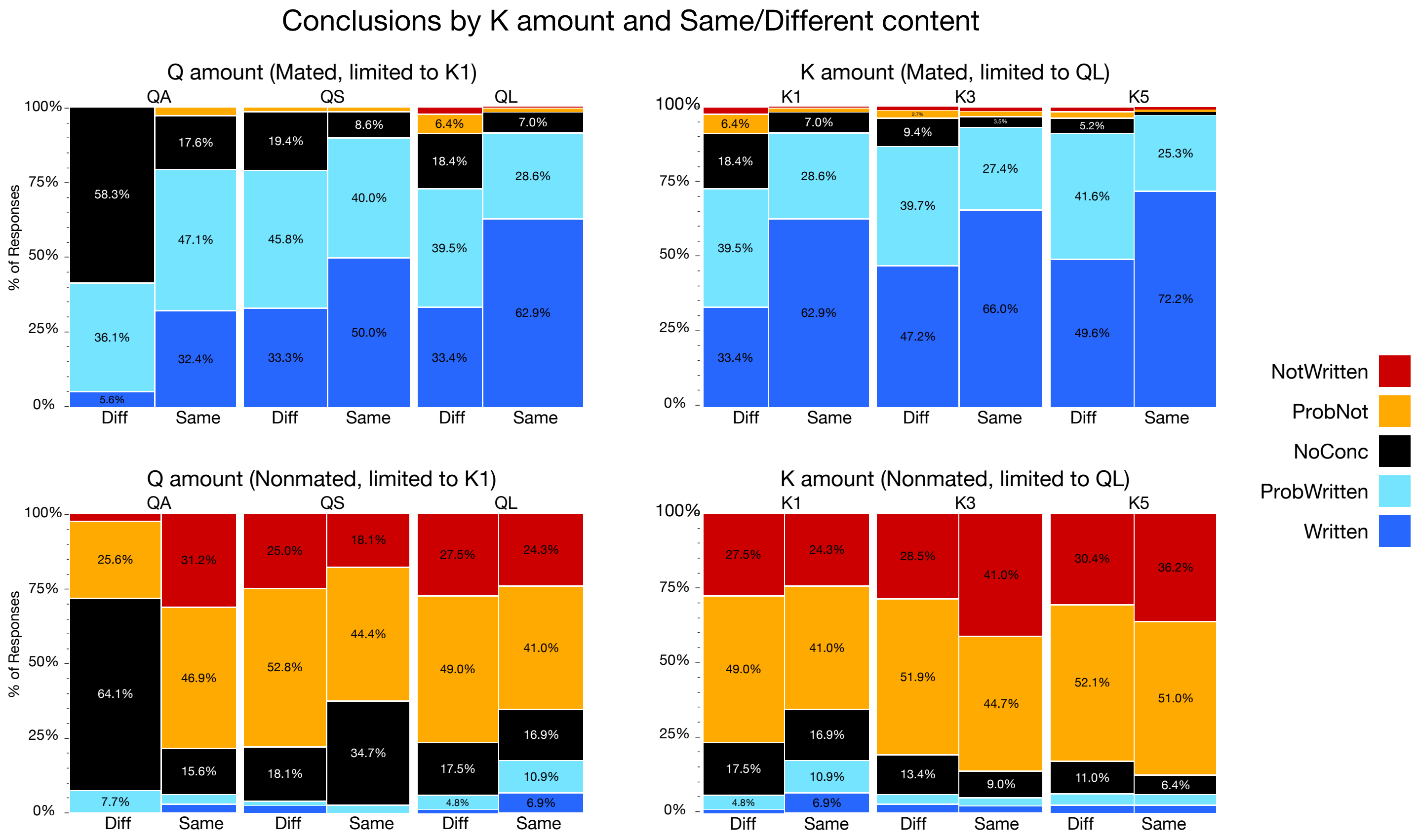

Fig S7. Q&K amount and Same/Different content. Left: effect of variation in Q amount and Same/Different content, limiting the length of the knowns to K1. Right: effect of variation in K amount and Same/Different content, limiting the length of the questioned samples to QL. (Subset of *Baseline Dataset*. Left: limited to K1; mated: 969 trials on 26 QKsets; nonmated: 1,394 trials on 38 QKsets. Right: limited to QL; mated: 2,225 trials on 60 QKsets; nonmated: 3,084 trials on 84 QKsets.)

Appendix H Repeatability of conclusions

Fig S8 and Table S21 summarize the repeatability (intra-examiner agreement) of conclusions reported by participants when given the same QKset on different occasions. For example (in the bottom row of the top chart in Fig S8), if a participant responded *Written* on the first assignment of a mated QKset, on the second assignment of that QKset that participant responded *Written* 83% of the time, or *ProbWritten* 17% of the time. As shown in Table S22 the vast majority of conclusions were either in perfect agreement (participants reported the same conclusion for both assignments) or near agreement (participants reported conclusions in adjacent categories for each assignment).

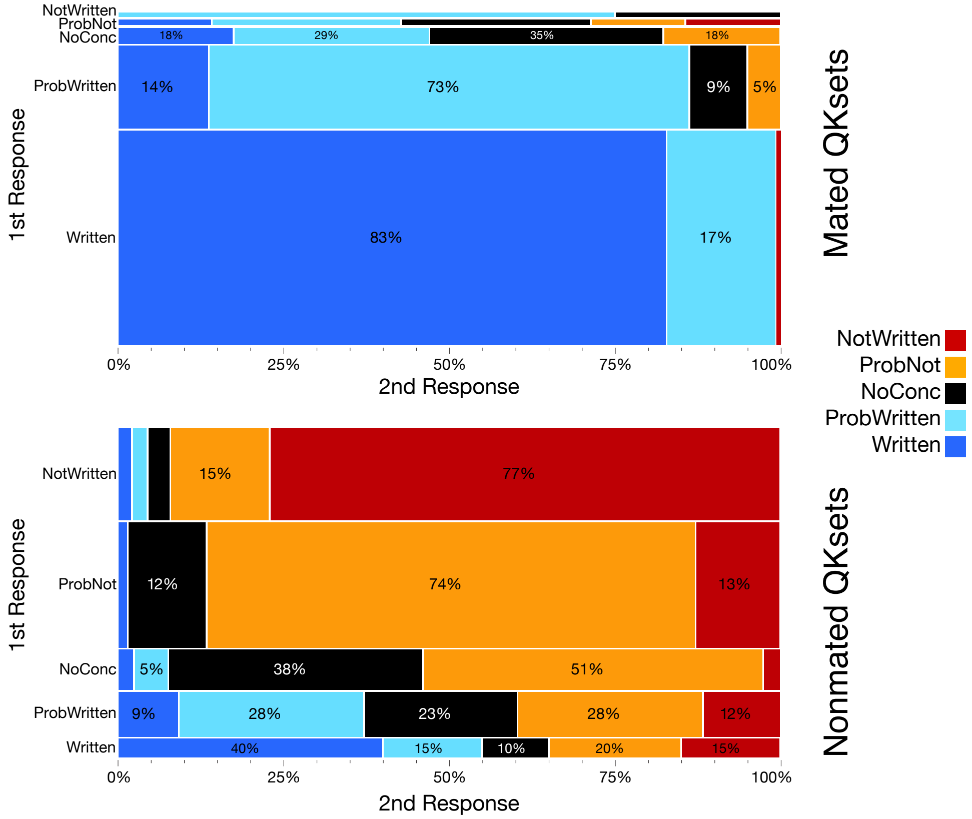

Fig S8. Repeatability of conclusions. Identical to the repeatability charts in Figure 5 (main paper) with the addition of labeled values. (*Repeatability Dataset*: 1240 responses (620 1st responses; 620 2nd responses) from 65 examiners on 20 QKsets)

Repeatability			2nd response				
			Written	*ProbWritten*	*NoConc*	*ProbNot*	*NotWritten*
1st response	*Mated*	*Written*	170	34	0	0	1
		ProbWritten	11	58	7	4	0
		NoConc	3	5	6	3	0
		ProbNot	1	2	2	1	1
		NotWritten	0	3	1	0	0
	Nonmated	*Written*	8	3	2	4	3
		ProbWritten	4	12	10	12	5
		NoConc	1	2	15	20	1
		ProbNot	2	0	14	87	15
		NotWritten	2	2	3	13	67

Table S21. Contingency table for repeatability data: data for Fig S8. (*Repeatability Dataset*: 620 pairs of responses from 65 examiners on 20 QKsets)

Repeatability	Rates			Cumulative			Counts		
	Mated	Nonmated	Total	Mated	Nonmated	Total	Mated	Nonmated	Total
Same	75.1%	61.6%	68.4%	75.1%	61.6%	68.4%	235	189	424
±1 conclusion	20.1%	26.4%	23.2%	95.2%	87.9%	91.6%	63	81	144
±2 conclusions	3.2%	6.2%	4.7%	98.4%	94.1%	96.3%	10	19	29
±3 conclusions	1.3%	4.2%	2.7%	99.7%	98.4%	99.0%	4	13	17
±4 conclusions	0.3%	1.6%	1.0%	100.0%	100.0%	100.0%	1	5	6
							313	307	620

Table S22. Repeatability of examiner conclusions by level of agreement. (*Repeatability Dataset*)

Fig S9 shows the relative effect of repeatability based on the length of the known writing for the QKset. Note that the high rate of repeated FPs in the top right chart is affected by the twins data: five of the eight repeated FPs were on QK472 (Figure 3, main paper).

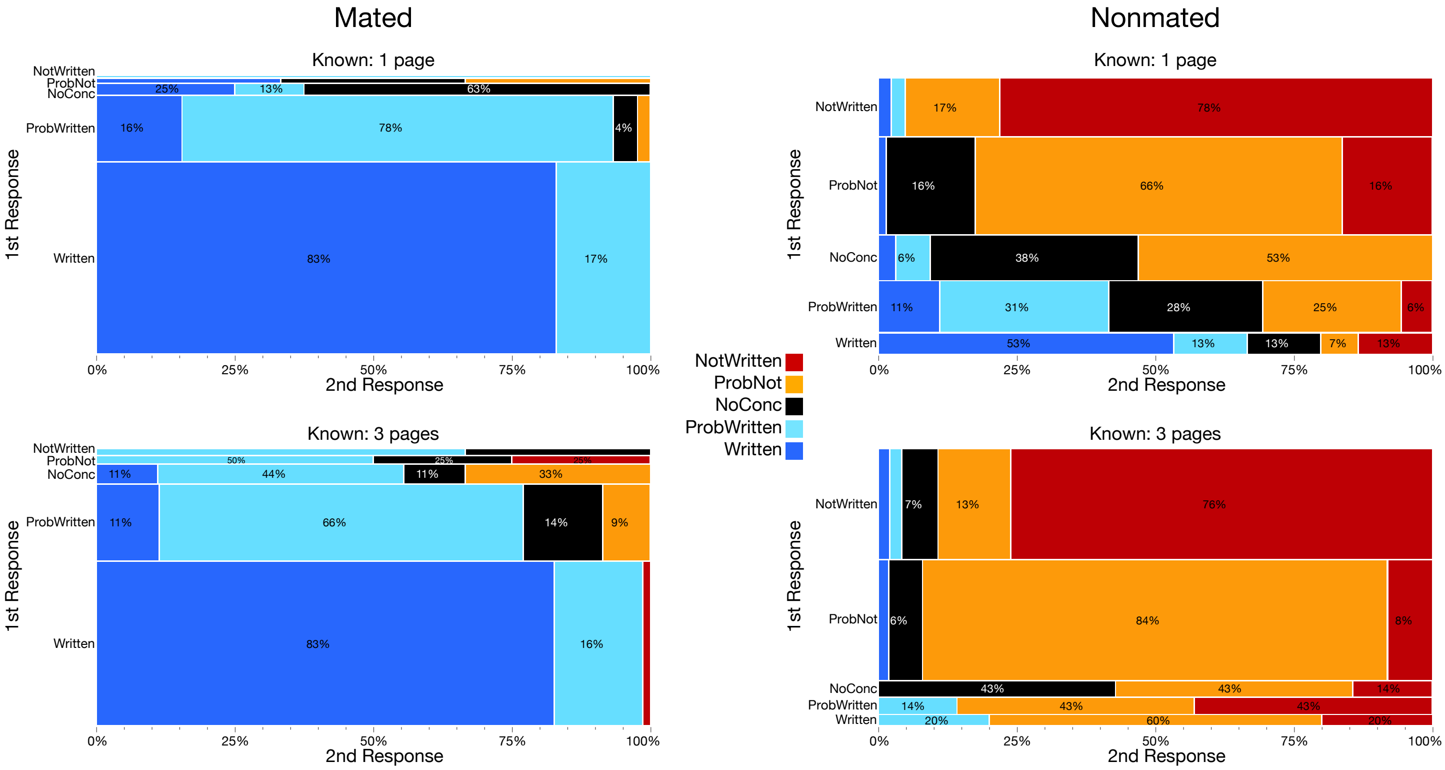

Fig S9. Repeatability of conclusions by length of known. (*Repeatability Dataset*: 620 pairs of responses from 65 examiners on 20 QKsets)

Repeatability was associated with the training groups (discussed in Appendix K1) as described in Table S23 and Fig S10: for participants with at least two years of formal training (Training Group A) 94.0% of conclusions were repeated within ±one conclusion category, as compared with 85.9% for training group B (p <0.001, based on chi-square analysis). However, this can be seen as an effect of the different error rates and rates of definitive conclusions: since Training Group A makes proportionally fewer definitive conclusions (and fewer errors), there is less of an opportunity to make conclusions that differ by two or more conclusion categories. Also see *Appendix F7* for discussion of repeatability of errors.

Delta	*Training Group*		*Total*
	A	*B*	
	2+ yrs formal training	*Less than 2 yrs formal training*	
Same	68%	69%	68%
±1 conclusion	26%	17%	23%
±2 conclusions	4%	6%	5%
±3 conclusions	1%	6%	3%
±4 conclusions	1%	2%	1%
±2 or more conclusions	6%	14%	8%

Table S23. Repeatability of examiner conclusions by training group. Of the 65 participants in the *Repeatability Dataset*, 46 were in Training Group A, and 19 were in Training Group B. (*Repeatability Dataset*)

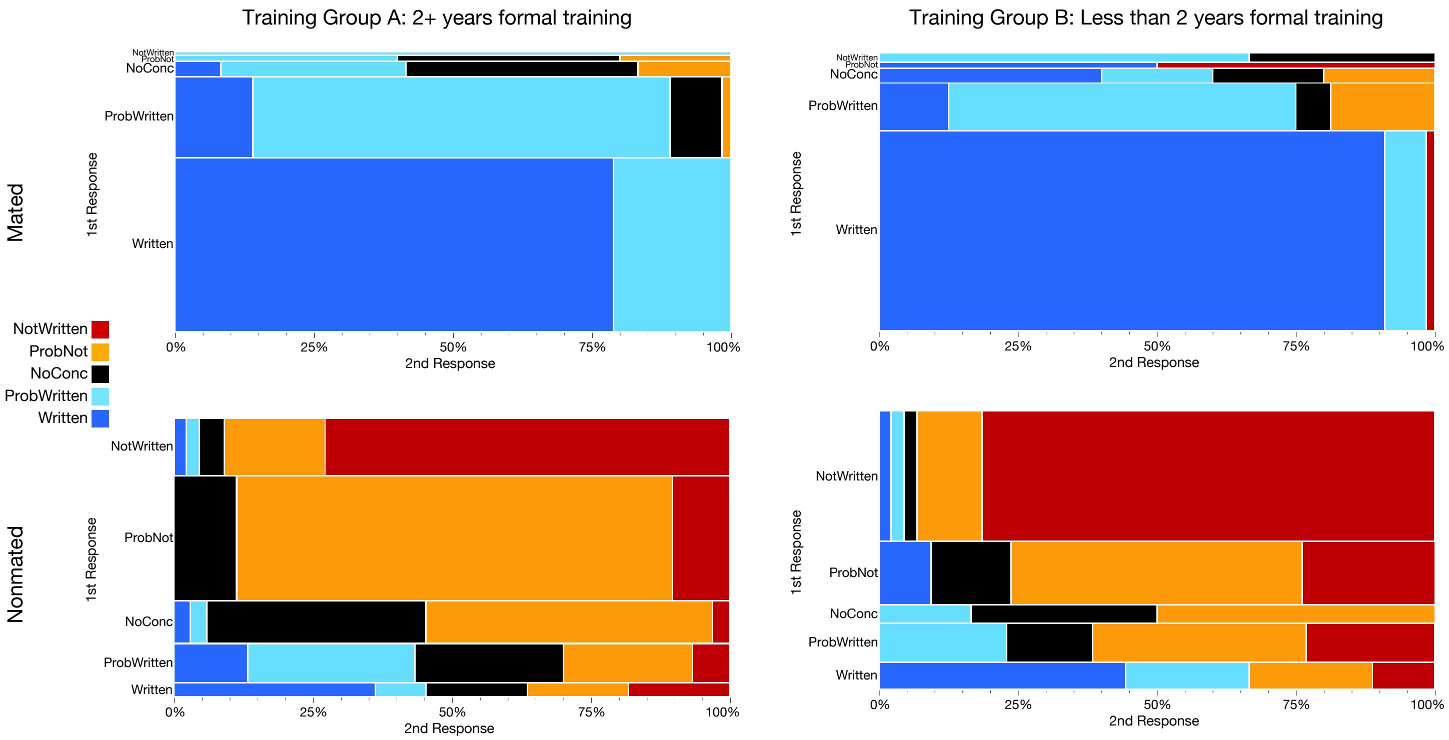

Fig S10. Repeatability of examiner conclusions by training group. (*Repeatability Dataset*. Training Group A: 220 mated and 215 nonmated pairs of responses from 63 participants; Training Group A: 93 mated and 92 nonmated pairs of responses from 23 participants.)

Appendix I Reproducibility of conclusions

Fig S11 and Table S24 summarize the reproducibility (inter-examiner agreement) of conclusions reported by participants on the same QKsets, based on all pair-wise combinations of responses from different participants on the same QKsets. For example (in the bottom row of the top chart in Fig S11), for every participant who responded *Written* on a mated QKset, 60% of the other participants also responded *Written*, 33% responded *ProbWritten*, and 5% responded *NoConc*. In contrast to the repeatability results (Fig S8), note that in general the distributions of responses in Fig S11 are remarkably similar regardless of conclusion type: particularly for nonmated data, the categories in Fig S11 are close to vertical rather than the diagonals one might expect; knowing one participant's response is a very weak predictor of other participants' responses.

As shown in Table S25, the vast majority of conclusions were either in perfect agreement (two participants reported the same conclusion) or near agreement (two participants reported conclusions in adjacent categories).

However, for both mated and nonmated comparison decision pairings, there were some conclusions that were contradictory (±4 categories; one participant reported *Written* and the other reported *NotWritten*) or contrary (±3 categories: one participant reported *Written* and the other reported *ProbNot*, or one participant reported *NotWritten* and the other reported *ProbWritten*):

- For mated comparison decision pairings: 0.9% contradictory decisions and an additional 2.3% contrary decisions
- For nonmated comparison decision pairings: 1.5% contradictory decisions and an additional 4.9% contrary decisions

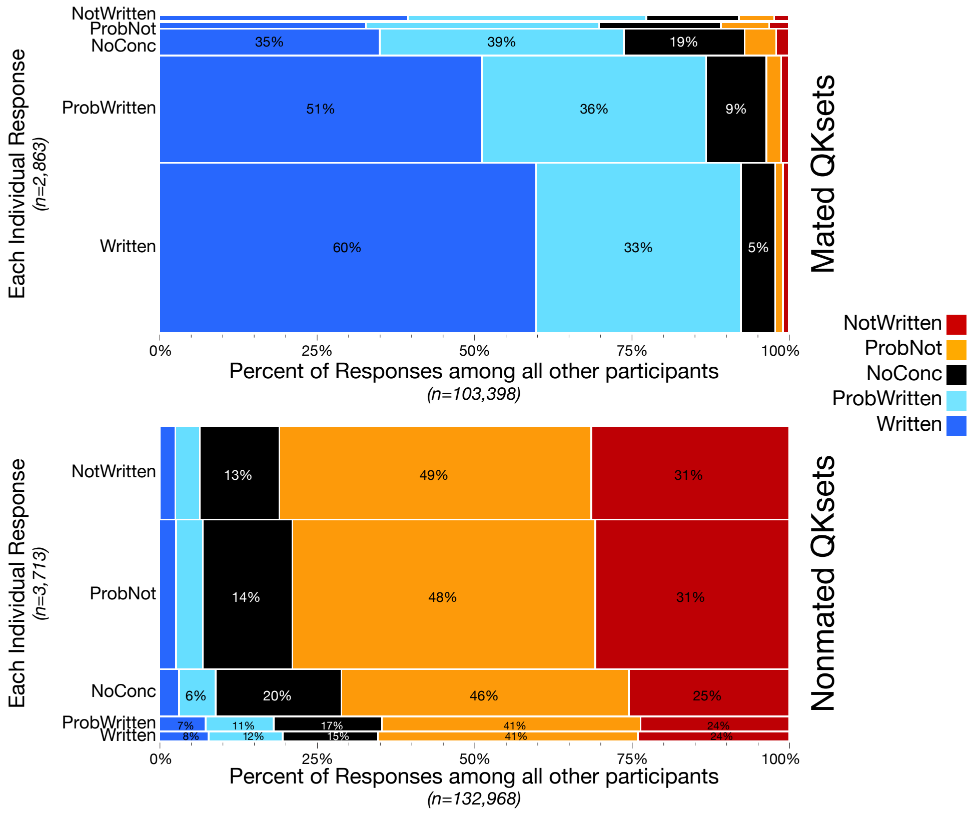

Fig S11. Mosaic displays of contingency tables for reproducibility of participants' conclusions. Identical to the reproducibility charts in Figure 5 (main paper) with the addition of labeled values. (*Baseline Dataset*).

Reproducibility			Examiner B				
			Written	*ProbWritten*	*NoConc*	*ProbNot*	*NotWritten*
Examiner A	*Mated*	*Written*	33,488	18,216	3,040	728	461
		ProbWritten	18,216	12,610	3,351	820	441
		NoConc	3,040	3,351	1,658	428	172
		ProbNot	728	820	428	170	66
		NotWritten	461	441	172	66	26
	Nonmated	*Written*	332	490	632	1,727	1,005
		ProbWritten	490	702	1,131	2,702	1,547
		NoConc	632	1,131	3,956	9,004	4,995
		ProbNot	1,727	2,702	9,004	30,342	19,440
		NotWritten	1,005	1,547	4,995	19,440	12,290

Table S24. Contingency table for reproducibility of examiner conclusions: data for Fig S11. (*Baseline Dataset*. 103,398 inter-examiner decision pairs derived from 2,863 trials on 78 mated QKsets; 132,968 inter-examiner decision pairs derived from 3,713 trials on 102 nonmated QKsets)

Reproducibility	Rates			Cumulative			Counts		
	Mated	Nonmated	Total	Mated	Nonmated	Total	Mated	Nonmated	Total
Same	46.4%	35.8%	40.4%	46.4%	35.8%	40.4%	47,952	47,622	95,574
±1 conclusion	42.7%	45.2%	44.1%	89.0%	81.0%	84.5%	44,122	60,130	104,252
±2 conclusions	7.8%	12.5%	10.5%	96.8%	93.6%	95.0%	8,064	16,658	24,722
±3 conclusions	2.3%	4.9%	3.8%	99.1%	98.5%	98.8%	2,338	6,548	8,886
±4 conclusions	0.9%	1.5%	1.2%	100.0%	100.0%	100.0%	922	2,010	2,932
							103,398	132,968	236,366

Table S25. Reproducibility of examiner conclusions by level of agreement.

Fig S12 and Table S26 show the relative effect if reproducibility is limited to the participants who reported all conclusions (discussed in Appendix J2.1), or by the training groups (discussed in Appendix K1). There is no notable effect if limited to the participants who reported all conclusions. Training Group B has a greater proportion

of conclusions that differ by 3 or 4 categories, which could be expected from the higher error rates and increased proportions of definitive conclusions (as compared to Training Group A).

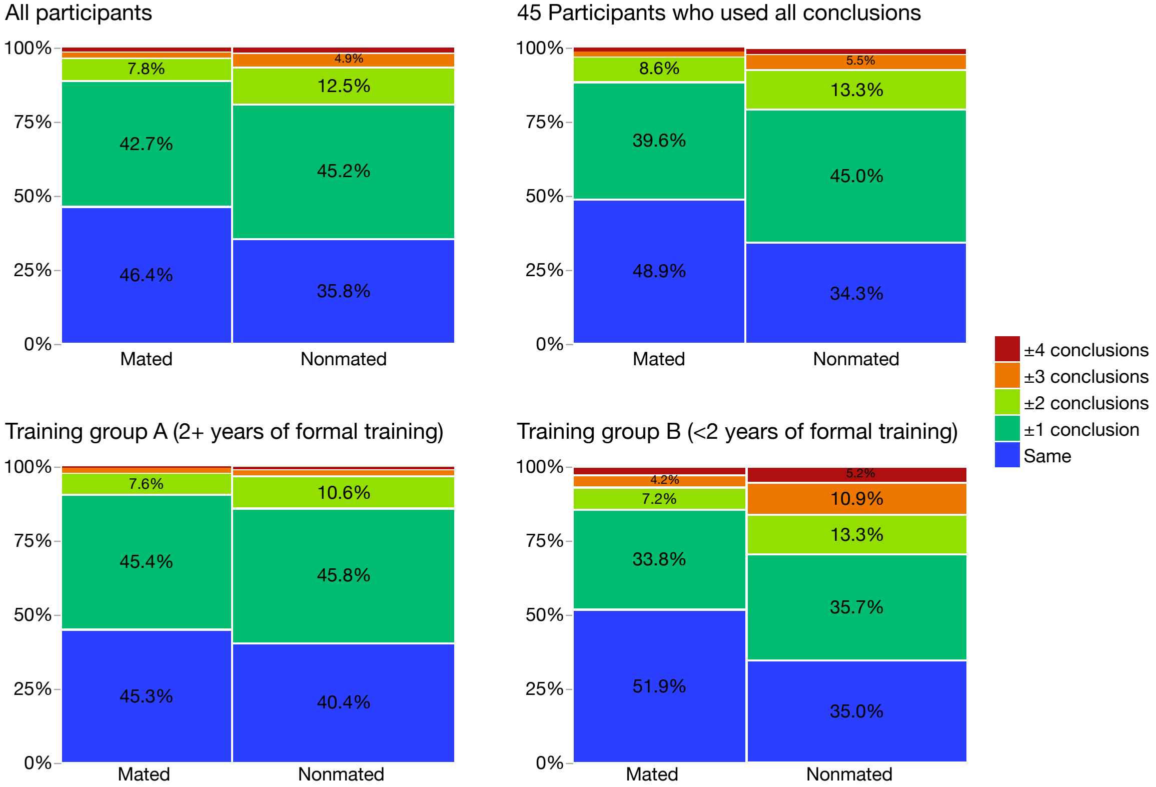

Fig S12. Reproducibility of examiner conclusions by level of agreement for various categories of participants. The top left chart depicts the data shown in Table S25. The other three charts show the effect if limited to (top right) the 45 participants who reported all conclusions (discussed in Appendix J2.1); (bottom left) participants with at least two years of formal training; (bottom right) participants with less than two years of formal training.

Delta	*All participants*	*Participants who used all conclusions*	*Training Group A*	*Training Group B*
Same	40.4%	40.7%	42.6%	42.5%
±1	44.1%	42.6%	45.6%	34.9%
±2	10.5%	11.3%	9.3%	10.6%
±3	3.8%	3.9%	2.2%	7.9%
±4	1.2%	1.5%	0.4%	4.2%
# Participants	86	45	63	23
Inter-examiner decision pairs	236,366	79,576	118,376	18,598

Table S26. Reproducibility of examiner conclusions by level of agreement for various categories of participants. (Same data as Fig S12.)

If different conclusions arise during the process of typical casework (e.g., during verification), the lack of reproducibility could help to flag errors or incorrect conclusions and might allow them to be properly addressed and rectified prior to reporting a final conclusion. However, such a verification mechanism would fail to detect potentially erroneous or incorrect conclusions if the two FDEs agreed upon the reported decision. Some errors and incorrect conclusions were reproduced in this study (i.e., if a participant made a conclusion contrary to ground truth, these are the proportions of other participants who also made a conclusion contrary to ground truth on the same QKset):

- 7.9% of erroneous *Written* decisions were reproduced exactly, and an additional 11.7% resulted in the second examiner reporting *ProbWritten*

- 2.2% of erroneous *NotWritten* decisions were reproduced exactly, and an additional 5.7% resulted in the second examiner reporting *ProbNot*
- 10.7% of incorrect *ProbWritten* decisions were reproduced exactly, and an additional 7.5% resulted in the second examiner reporting *Written*
- 7.7% of incorrect *ProbNot* decisions were reproduced exactly, and an additional 3.0% resulted in the second examiner reporting *NotWritten*

Appendix J Participant effects

Appendix J1 Comparing participants

When measuring individual participant rates, we limit analyses to the 70 participants who completed at least half of their assigned comparisons (denoted *Examiner Comparison Dataset* in Table S8).

Figure 4 in the main paper details error rates (erroneous and incorrect conclusions) and accuracy rates (true and correct conclusions) for the 70 participants included in the *Examiner comparison dataset*. However, in order to allow for a comparison of the relative performance of participants, it is necessary to simultaneously incorporate three inter-related decision factors: correctness, definitiveness, and the relative value/cost of making definitive vs qualified conclusions.

- Correctness: considers the participant's conclusions with respect to the ground truth regarding writership for the given QKset (e.g., decisions of *ProbWritten* or *Written* would be considered correct for mated QKsets).
- Definitiveness: considers the participant's reporting tendencies across the conclusion scale, not just on the extremes (e.g., a conservative participant may rarely report definitive conclusions and prefer to report qualified conclusions, but this tendency should be accounted for in the assessment of performance).
- Relative value/cost: considers the added value of a correct definitive over qualified conclusion or the added cost of an incorrect definitive over qualified conclusion (e.g., an erroneous *Written* conclusion has more potentially severe consequences than an incorrect *ProbWritten*).

For the purposes of these analyses, qualified conclusions are weighted as half of definitive conclusions in both relative value and cost; at this time, there are no recommendations in the literature that inform these weights, so these values were selected as an approximation.

In order to account for these factors, we developed four weighted rates:

- Weighted TP-CA rate = TPR_{PRES} + 0.5(CAR_{PRES})
- Weighted TN-CN rate = TNR_{PRES} + 0.5(CNR_{PRES})
- Weighted FP-IA rate = FPR_{PRES} + 0.5(IAR_{PRES})
- Weighted FN-IN rate = FNR_{PRES} + 0.5(INR_{PRES})

Because these rates have notably different ranges, we converted each of these into ***ratios*** describing each participant's performance relative to the other participants in the study (displayed in Fig S13). Each ratio contrasts a participant's individual weighted reporting rate versus the average of that weighted reporting rate across all participants: for example, a weighted TP-CA ratio of 2.0 means that participant's weighted TP-CA rate was twice the average.

As an example of these computations, consider the red asterisk located in Fig S13. This participant had a false negative rate (FNR_{PRES}) of 18% and an incorrect negative association rate (INR_{PRES}) of 8%; therefore, this participant's weighted FN+IA rate is 22% (18%+0.5(8%)). The average weighted FN+IA rate across all other participants is 1.7%. Therefore, this participant's weighted FP-IA ratio is 12.9 (22%/1.7%), as shown along the x-axis in Fig S13 (top-right). This value indicates that this participant made nearly 13x the weighted number of *NotWritten* and *ProbNot* conclusions that an average examiner made on assigned mated QKsets.

As illustrated in Fig S13, relative performance is highly variable between participants, however some trends are readily observed and highlight the inter-relatedness of the three decision factors encompassed by these ratios. Participants who were least definitive (black symbols) generally had lower than average rates of incorrect conclusions (FP-IA and FN-IN ratios), but also lower than average rates of correct conclusions (TP-CA and TN-CN ratios). On the other hand, participants who were most definitive (blue symbols) generally had higher than average ratios of correct conclusions (TP-CA and TN-CN), but often higher ratios of at least one type of incorrect conclusion (FP-IA and/or FN-IN ratios), with a few exceptions who had relatively few errors.

In addition, it is worth acknowledging that some participants' performance rates, and consequently their relative performance ratios may be impacted by the assigned QKsets. Some QKsets had notably different error rates (e.g.,

QKset #472 displayed in Figure 3 had 13 false positives on the *Baseline Dataset*); rates for participants assigned these comparisons may be impacted (e.g., higher FPR than average).

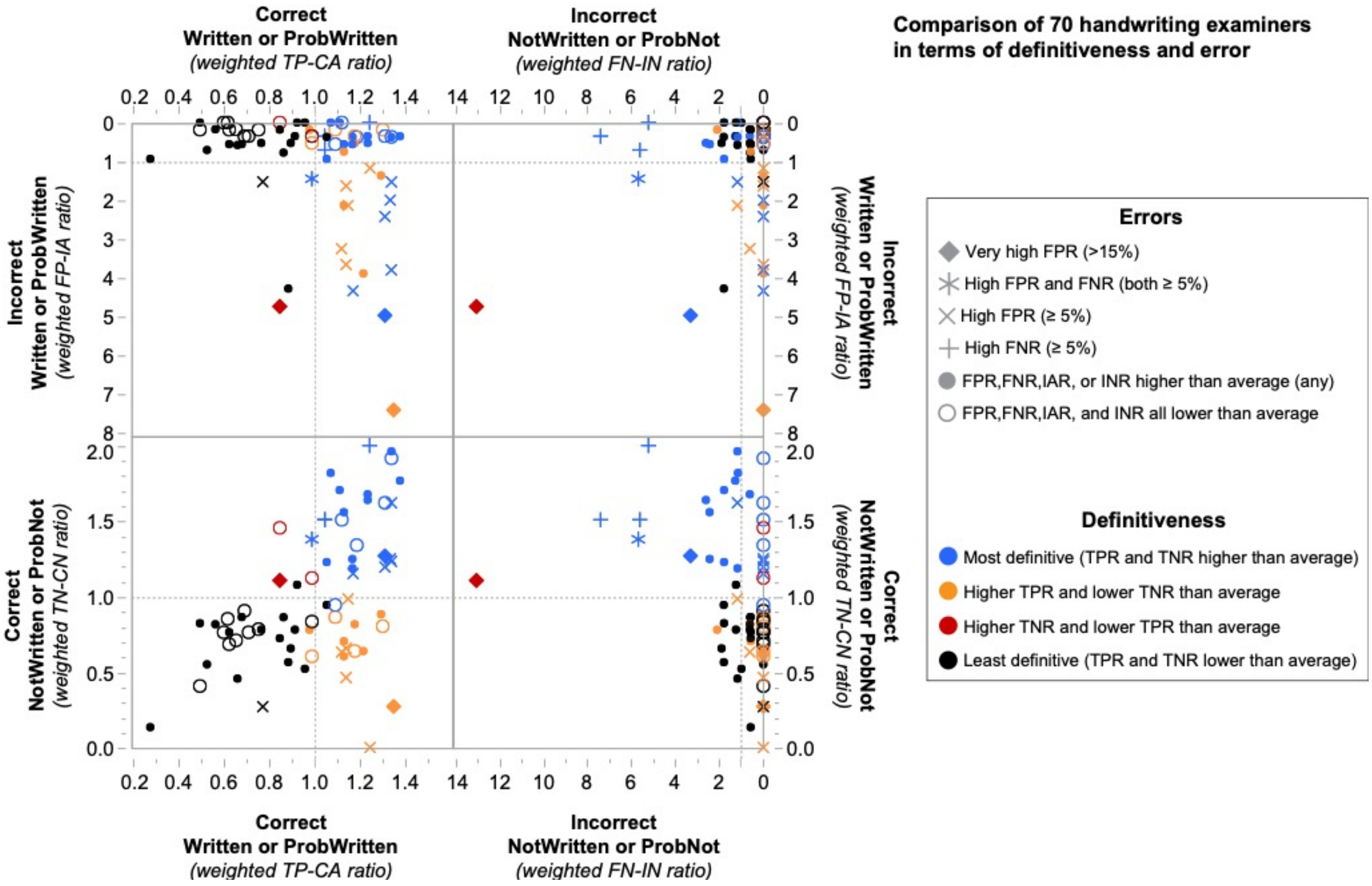

Fig S13. Comparison of participants by weighted performance ratios. The symbols and colors are the same for Fig S13 & Fig S14 (*Appendix J1*), and Figure 4 (main paper). (n=70; *Examiner comparison dataset*)

Fig S14 provides a different depiction of the data shown in Figure 4 in the main paper, and shows the proportions of indeterminate, qualified, and definitive responses with respect to ground truth. Fig S14 (left chart) plots the 70 participants in the *Examiner comparison dataset* in terms of correct qualified and definitive responses (x axis) vs. incorrect qualified and definitive responses (y axis). Note that there is an implicit third axis in this chart showing the proportion of residual indeterminate (*NoConc*) responses: participants who made no *NoConc* responses (i.e., only qualified and definitive) are shown on the solid gray diagonal (top left to bottom right), whereas participants who made a majority of *NoConc* responses are shown below the dashed gray diagonal (i.e., more indeterminate responses than definitive and qualified combined); if there were participants who made only *NoConc* responses, they would appear at the origin (0%,0%). No participants made more incorrect than correct responses (i.e., all participants are below and right of the solid green diagonal), but for a few participants more than one quarter of responses were incorrect (above the dotted green diagonal).

Fig S14 (right chart) plots the same data, but is limited to definitive responses. Here the residual (implicit third axis) is the proportion of indeterminate and qualified responses: the solid gray diagonal here indicates participants who made no *NoConc, ProbWritten, or ProbNot* responses, whereas participants below the dashed gray diagonal made more indeterminate and qualified responses than definitive responses.

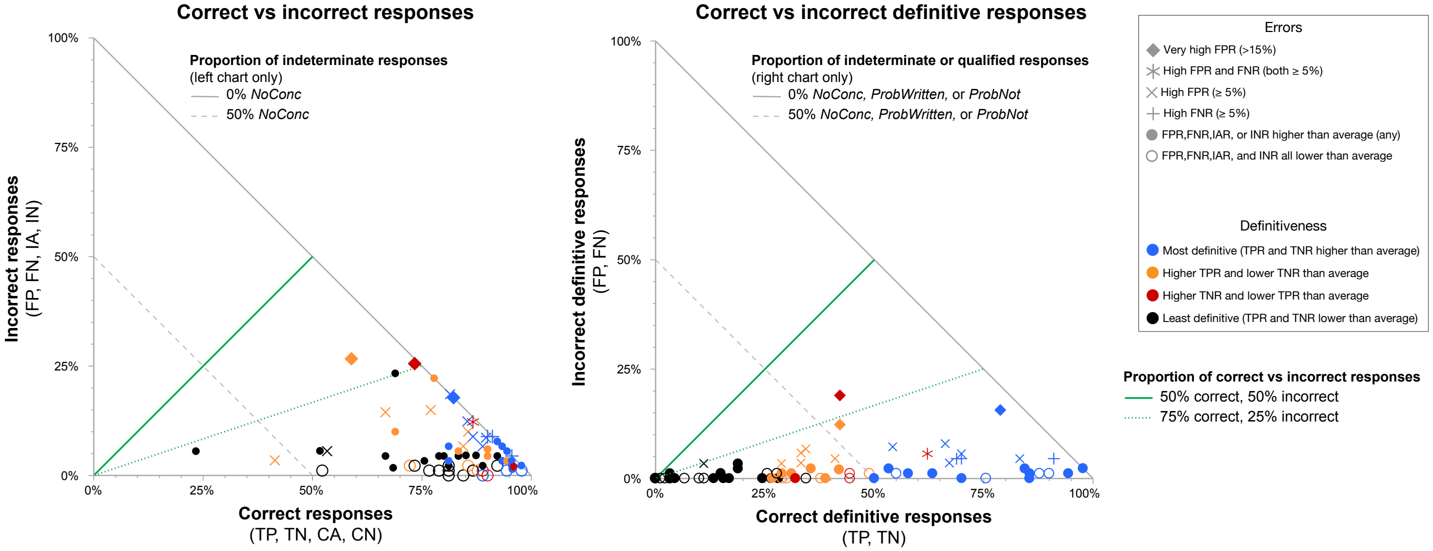

Fig S14. Comparison of participants by proportions of correct vs correct responses. The symbols and colors are the same for Fig S13 & Fig S14 (*Appendix J1*), and Figure 4 (main paper). (n=70; *Examiner comparison dataset*)

The charts in Fig S14 provide a means to consider the different dimensions by which examiner skill can be assessed. Ideally, examiners would be at or near the bottom right corner of these charts (high percentage of correct responses). To the extent that examiners do not always provide a correct response, it is far preferable to have an indeterminate response than an incorrect response: if examiners are not at the bottom right corner of these charts, then it is preferable to be as close as possible to the horizontal axis (low percentage of incorrect responses). Examiners with a high proportion of indeterminate responses are not committing errors, but are ineffective: examiners with many indeterminate responses cannot have a high proportion of correct responses.

Appendix J2 Conclusions by participant

Fig S15 and Fig S16 detail decision rates on mated and nonmated QKsets for each of the 70 participants in the *Examiner comparison dataset*, which allows for a visualization of the variation in examiner-specific reporting tendencies. The same rates are shown in table form in Table S27 (also included as a spreadsheet in *Supplemental Information SI-1*).

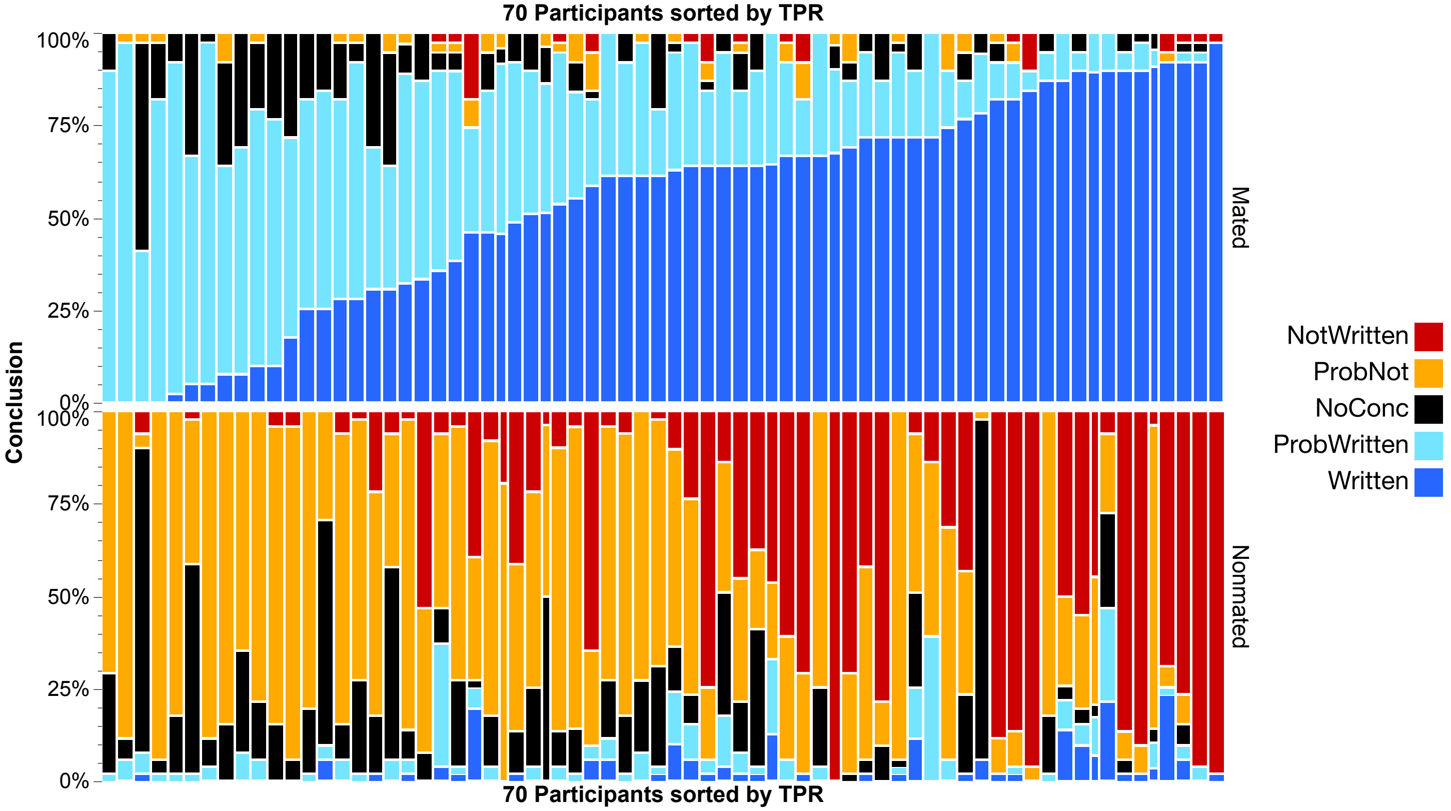

Fig S15. Decision rates by examiner, sorted by TPR. (*Examiner comparison dataset*)

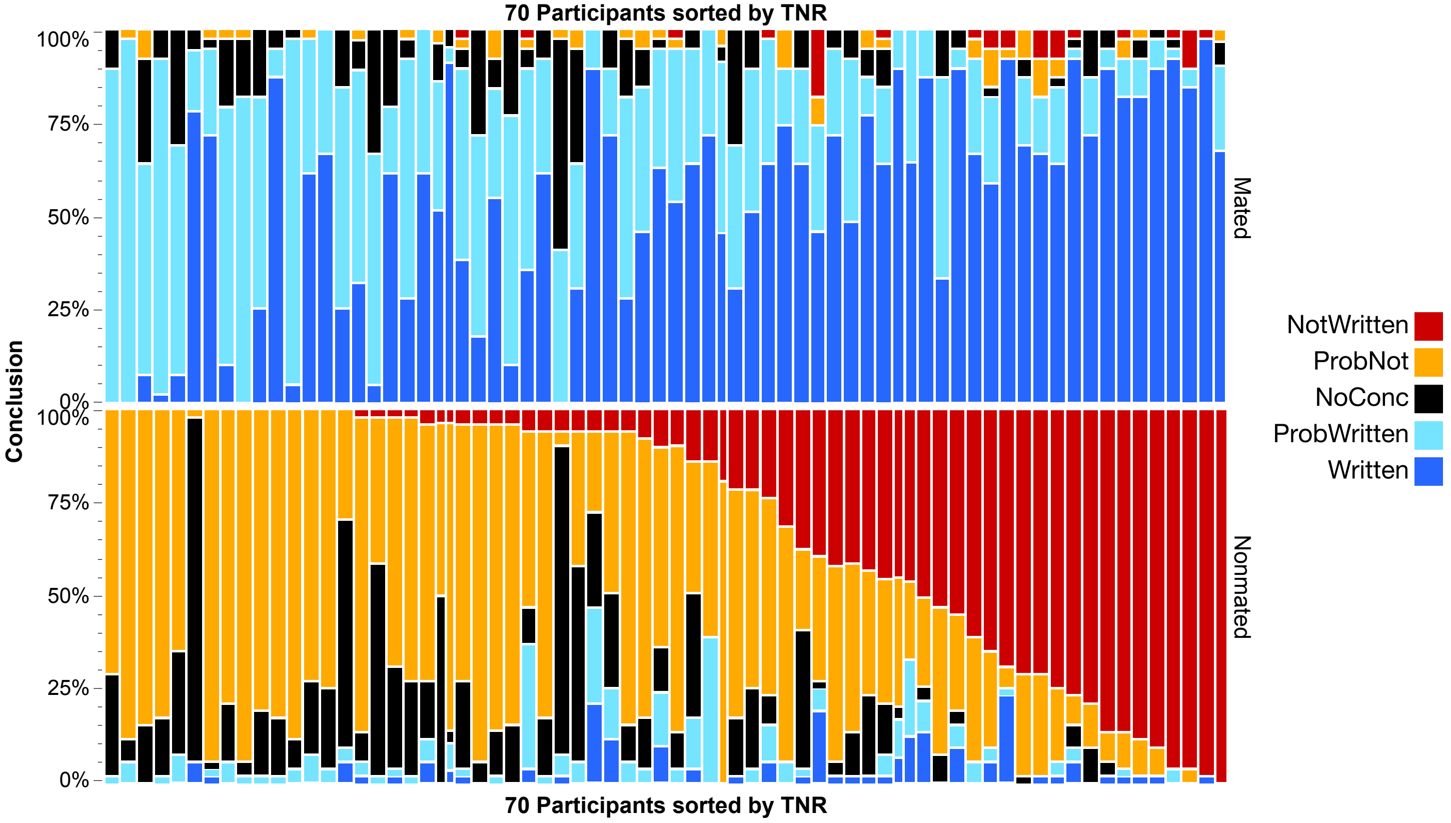

Fig S16. Decision rates by examiner; same data as Fig S15, but sorted by TNR. (*Examiner comparison dataset*)

Participant (AnonID)	*Mated assignments*	*TPR*	*CAR*	*INR*	*FNR*	*Nonmated assignments*	*FPR*	*IAR*	*CNR*	*TNR*
AH1C7C	39	46%	28%	8%	18%	51	20%	6%	33%	39%
AH1D87	39	67%	26%	5%	3%	51	0%	6%	33%	61%
AH2E03	39	0%	90%	0%	0%	51	0%	2%	71%	0%
AH2E42	39	92%	0%	3%	5%	51	24%	2%	6%	69%
AH2EEA	39	92%	3%	0%	3%	51	6%	4%	8%	76%
AH3D9C	39	0%	97%	3%	0%	51	0%	6%	88%	0%
AH4C77	39	69%	18%	8%	0%	51	0%	0%	27%	71%
AH4E27	39	36%	54%	3%	3%	51	4%	33%	47%	6%
AH7F46	39	8%	56%	8%	0%	51	0%	0%	84%	0%
AH9CBB	39	31%	38%	0%	0%	51	2%	0%	61%	22%
AH9E24	38	63%	32%	3%	0%	49	10%	14%	53%	10%
AH9E98	39	92%	3%	0%	3%	50	0%	4%	0%	96%
AH29E9	39	72%	23%	0%	0%	50	2%	0%	52%	42%
AH31B4	39	62%	38%	0%	0%	51	6%	6%	69%	4%
AH41F7	38	97%	0%	0%	3%	50	2%	0%	0%	98%
AH43D1	39	67%	15%	10%	8%	51	2%	0%	27%	71%
AH46C3	37	32%	57%	3%	0%	50	2%	4%	84%	2%
AH48AB	39	46%	38%	5%	0%	51	0%	4%	75%	8%
AH50DD	39	62%	31%	0%	0%	51	0%	2%	76%	6%
AH52B8	29	52%	34%	3%	0%	28	0%	0%	46%	4%
AH57C7	22	91%	5%	0%	0%	28	4%	7%	82%	4%
AH64FC	39	38%	51%	3%	3%	51	2%	2%	69%	4%
AH66AF	39	64%	33%	0%	3%	51	6%	10%	53%	24%
AH75E4	39	18%	54%	0%	0%	51	0%	0%	90%	4%
AH78E1	39	3%	90%	0%	0%	51	0%	2%	82%	0%
AH88DD	39	72%	15%	0%	0%	51	0%	0%	12%	78%
AH89A8	39	90%	5%	0%	0%	51	10%	6%	25%	55%
AH166B	39	82%	10%	3%	0%	51	2%	0%	10%	88%
AH418D	38	55%	29%	8%	0%	49	0%	2%	82%	4%
AH430A	39	8%	62%	0%	0%	51	0%	8%	65%	0%
AH519A	37	78%	16%	0%	0%	50	6%	0%	2%	0%
AH588E	39	64%	21%	5%	8%	51	2%	4%	20%	75%
AH668A	39	54%	41%	3%	3%	51	0%	4%	76%	10%
AH1799	39	72%	23%	3%	0%	51	2%	2%	94%	0%
AH2464	39	64%	31%	0%	0%	51	4%	14%	35%	14%
AH2823	39	77%	10%	5%	0%	51	2%	0%	33%	43%
AH4258	39	33%	54%	0%	0%	51	0%	0%	39%	53%
AH4284	39	64%	21%	3%	3%	51	2%	6%	33%	45%
AH7862	39	5%	62%	0%	0%	51	0%	2%	39%	2%
AH8112	39	10%	69%	3%	0%	51	0%	6%	78%	0%
AH9207	31	65%	35%	0%	0%	39	13%	21%	21%	46%
AH9854	29	90%	10%	0%	0%	29	7%	10%	34%	45%
AH9991	39	0%	41%	3%	0%	51	2%	6%	4%	6%
AHA6FC	31	68%	23%	3%	0%	38	0%	0%	0%	100%
AHA7B7	39	0%	82%	3%	0%	51	0%	2%	94%	0%
AHA318	39	31%	33%	5%	0%	50	0%	6%	36%	6%
AHA471	39	90%	10%	0%	0%	51	22%	25%	22%	6%
AHABB9	39	26%	56%	0%	0%	51	0%	2%	80%	0%
AHACF5	39	64%	26%	0%	0%	51	2%	2%	22%	37%
AHB94B	39	87%	8%	0%	0%	51	0%	2%	82%	0%
AHB925	39	72%	18%	0%	0%	51	12%	14%	43%	6%
AHBB3D	39	51%	38%	0%	0%	51	0%	4%	53%	22%
AHC4E4	39	49%	44%	0%	0%	51	2%	0%	45%	41%
AHC982	39	5%	92%	0%	0%	51	0%	4%	88%	0%
AHCAD2	39	90%	5%	0%	0%	51	2%	0%	8%	86%
AHD99D	39	28%	54%	3%	0%	51	0%	6%	78%	6%
AHD282	39	85%	5%	0%	10%	51	0%	0%	4%	96%
AHDCAF	39	72%	28%	0%	0%	51	0%	39%	47%	14%
AHDDD2	39	62%	36%	3%	0%	51	0%	8%	73%	0%
AHDE47	39	67%	33%	0%	0%	51	0%	4%	75%	0%
AHDFF3	39	90%	8%	0%	0%	51	2%	0%	8%	90%
AHE44F	39	62%	18%	0%	0%	51	2%	2%	67%	2%
AHE118	39	74%	15%	10%	0%	51	0%	6%	63%	31%
AHEDDA	39	10%	67%	0%	0%	51	0%	0%	80%	4%
AHEF6E	39	59%	23%	10%	5%	51	6%	4%	25%	65%
AHF048	39	87%	13%	0%	0%	50	14%	8%	24%	50%
AHF96D	39	82%	10%	5%	3%	51	2%	2%	10%	86%
AHF627	24	46%	46%	4%	0%	26	0%	0%	81%	19%
AHFA0E	39	28%	64%	3%	0%	51	0%	2%	71%	2%
AHFC73	39	26%	59%	0%	0%	51	6%	4%	29%	0%

Table S27. Decision rates by examiner. (*Examiner comparison dataset*)

Appendix J2.1 Avoidance of specific conclusions by participants

Table S28 and Table S29 show that some participants avoided specific conclusions. Out of the 70 participants in the *Examiner Comparison Dataset*, only 45 used every type of conclusion. Three participants (all with at least two years of formal training) never made any definitive conclusions (*Written* or *NotWritten*) and an additional 12 never made any *NotWritten* conclusions. One participant only made definitive conclusions (i.e., never made any *ProbWritten*, *ProbNot*, or *NoConc* responses). Nine participants never made *NoConc* responses.

	Participants who never made a given conclusion				
	Written	*NotWritten*	*ProbWritten*	*ProbNot*	*NoConc*
All participants	3	15	1	2	9
2+ years training	3	15	0	1	2
<2 years training	0	0	1	1	7

Table S28. Counts of participants who never made a given conclusion. (*Examiner Comparison Dataset*, n=70 participants)

Combinations					*Participants*		
Written	*NotWritten*	*ProbWritten*	*ProbNot*	*NoConc*	*Total*	*2+ years training*	*<2 years training*
-	-	-	-	-	45	30	15
-	-	-	-	Never	8	2	6
-	-	-	Never	-	1	1	0
-	-	Never	Never	Never	1	0	1
-	Never	-	-	-	12	12	0
Never	Never	-	-	-	3	3	0

Table S29. Counts of participants who never made combinations of conclusions (detail of Table S28). For example: (top row) 45 participants made at least one of each type of response in the study; (bottom row) three participants never made any *Written* or *NotWritten* responses. (*Examiner Comparison Dataset*, n=70 participants)

Appendix J2.2 Definitiveness among participants

Fig S17 illustrates participant definitiveness as a function of their training and experience; in particular, this figure contrasts definitive conclusion rate (proportion of decisions that were *Written* or *NotWritten*) versus probable or qualified conclusion rate (proportion of decisions that were *ProbWritten* or *ProbNot*). Participants who fall below the dashed diagonal report more indeterminate responses (*NoConc*) than definitive and qualified responses combined. The participant at the top left-hand corner of both charts was the most definitive participant in this study, with a decision of *Written* or *NotWritten* on all 88 comparisons completed by this individual in the *Baseline Dataset*; two of those conclusions were erroneous (one false negative, one false positive).

Based upon a Kruskal-Wallis analysis, definitiveness differed significantly as a function of both training and experience:

- Participants who had at least 2 years of formal training (were not self-trained, did not testify during training) tended to have higher rates of qualified conclusions than those who did not have such training ($p = 0.0064$). Alternatively, participants who did not have at least 2 years of formal training generally exhibited higher proportions of definitive conclusions ($p = 0.0015$).
- Participants with more than ten years of experience reported lower rates of qualified conclusions than those who had ten years or less of experience ($p = 0.0026$). Alternatively, participants with ten years or less of experience were significantly less definitive than their more experienced counterparts ($p = 0.0235$).

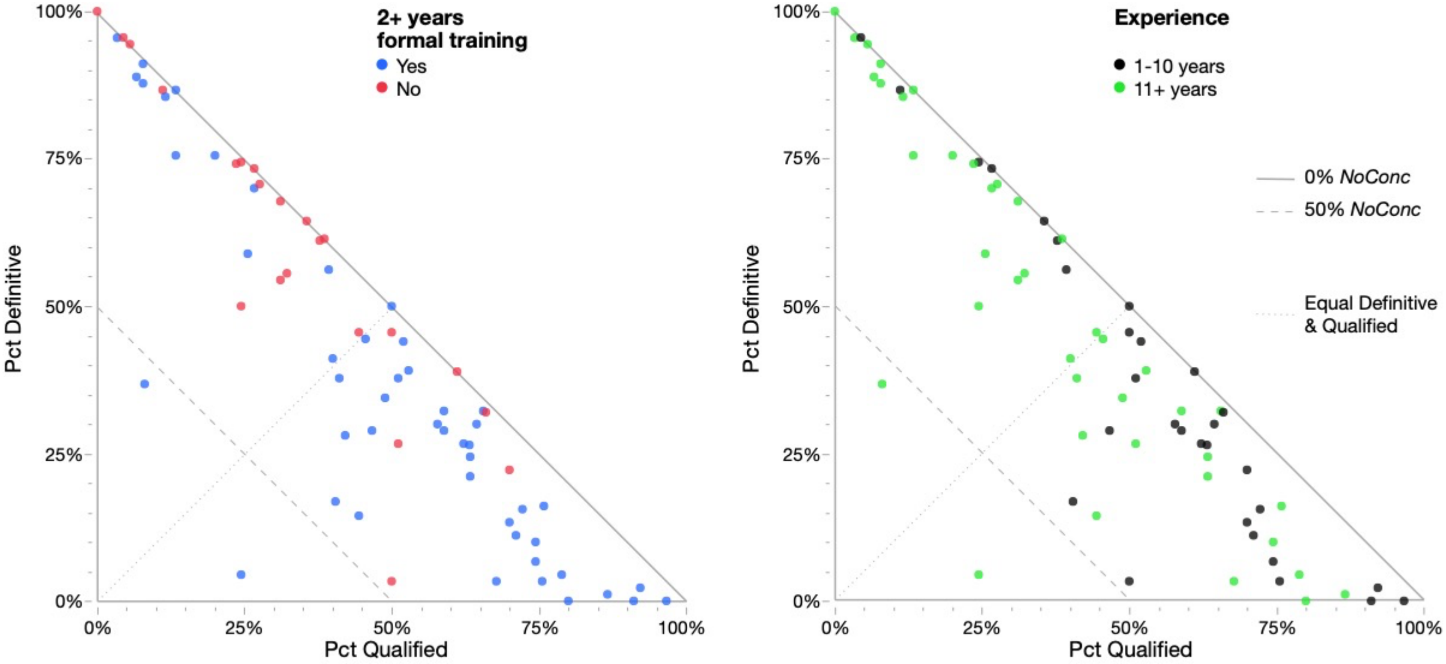

Fig S17. Definitive vs qualified conclusions among examiners as a function of training program and experience. Participants on the solid diagonal (top left to bottom right) made no *NoConc* responses. Participants below and left of the dashed diagonal made more *NoConc* responses than definitive and qualified responses combined. Participants below and right of the dotted diagonal (bottom left to top right) made more definitive responses than qualified responses. (N=70 examiners, *Examiner comparison dataset*)

Appendix K Associations with participant background

Appendix K1 Effect of 2 years of formal training

As discussed in the main paper (*Section 4.5, Associating participant performance with background),* we divided participants into two groups based on training:

- Training Group A – 63 participants who received two or more years of training under the supervision of a principal trainer and did not testify during their training.
- Training Group B – 23 participants who received less than two years of formal training, and/or noted they were self-trained, and those who testified about handwriting examinations prior to completing their training.

FDEs are largely trained through apprenticeship programs that involve a significant quantity of hands-on experience, shadowing, lecture, study of literature, and supervised casework. Though training programs have historically varied across institutions, in 2013 the Scientific Working Group for Document Examination (SWGDOC) published their *Standard for Minimum Training Requirements for Forensic Document Examiners* (22). In particular, Section 6.1 of this standard recommends that "the training program shall be the equivalent of a minimum of 24 months full-time training under the supervision of a principal trainer." In addition, the ANSI National Accreditation Board's (ANAB) *Guiding Principles of Professional Responsibility for Forensic Service Providers and Forensic Personnel* (23) particularly asserts that with respect to training "ethical and professionally responsible forensic personnel […] are properly trained and determined to be competent through testing prior to undertaking the examination of the evidence."

Sixty-three met the requirements to be included in Training Group A. It should be noted that while sixty-nine participants replied that they had 2 or more years of formal training ("I completed a formal, in-person program of instruction for 2 years or more"), we assessed 6 of these as not meeting the SWGDOC requirements for formal training: 3 of the 69 said that they were "Self-trained", and an additional 3 said that they testified as an expert during training ("Did you ever testify as an expert in handwriting examination while still in handwriting training?")

Figure 1 (main paper) and Table S30 partition the conclusion rates by training, as described above; there is a notable association between training and performance based upon a chi-square analysis. Participants in Training Group B, generally had higher error rates (FPR, FNR) and tended to give more definitive opinions (concluding *Written* or *NotWritten*), but also had higher rates of correct conclusions (TPR, TNR). In contrast, participants in Training Group A, had lower error rates (FPR, FNR) and were more conservative with definitive opinions, but they also had higher predictive values (PPV, NPV). While the TPRs and TNRs for Training Group A are lower, the groups have nearly the same rates for TPR+CAR (89.3% vs 88%) and same rate for TNR+CNR (77.4%), respectively.

The trend seen in Training Group B in which a larger quantity of definitive conclusions was given, resulting in more correct answers but higher error rates, was also seen in Kam's 1997 study (11) comparing FDE versus layperson performance. In that study, laypersons were found to "over-match" providing both a high TPR and high FPR as well. While participants in this study are not laypeople, a statistically significant difference in error rates is apparent based on the type of training received.

Differences between the training groups were compared using a chi-square analysis (with Bonferroni-adjusted Pearson residual post-hoc tests (24):

- Global effects are significant for both mated and nonmated pairs
- Bonferroni-adjusted Pearson residual posthoc for mated pairs: Training Group B exhibited many more FNs than expected ($p < 0.001$ for conclusions of *NotWritten*), while Training Group A exhibited much fewer FNs than expected; Training Group B was much more definitive than expected (more conclusions of both Written and NotWritten, $p < 0.001$), while Training Group A was much less definitive.
- PPV comparison: Training Group A had a higher proportion of correct *Written* than Training Group B ($p < 0.001$)

- NPV comparison: Training Group A had a higher proportion of correct *NotWritten* than Training Group B ($p < 0.001$)

	Baseline Dataset (86 participants)				*Training Group A 2 or more years of formal training (63 participants)*				*Training Group B Less than 2 years of formal training (23 participants)*			
	Mated		*Nonmated*		*Mated*		*Nonmated*		*Mated*		*Nonmated*	
Written	1546	54.0%	114	3.1%	1004	49.6%	51	1.9%	542	64.8%	63	5.8%
ProbWritten	982	34.3%	179	4.8%	777	38.4%	93	3.5%	205	24.5%	86	8.0%
NoConc	243	8.5%	547	14.7%	199	9.8%	452	17.2%	44	5.3%	95	8.8%
ProbNot	60	2.1%	1774	47.8%	40	2.0%	1451	55.1%	20	2.4%	323	30.0%
NotWritten	32	1.1%	1099	29.6%	6	0.3%	588	22.3%	26	3.1%	511	47.4%
Total trials	*2863*		*3713*		*2026*		*2635*		*837*		*1078*	

Table S30. Conclusion rates by training. Conclusions contradicting ground truth are highlighted. These results are shown graphically in Figure 1 (main paper). (*Baseline Dataset*)

Table S31 provides a further detailed breakdown of performance based on the training groups.

	Training Group A 2 or more years formal training	*Training Group B Less than 2 years formal training*
Positive predictive value (PPV)	95.2%	89.6%
Negative predictive value (NPV)	99.0%	95.2%
True positive rate (TPR_{PRES})	49.6%	64.8%
Correct positive association rate	38.4%	24.5%
True positive + Correct positive association rate	87.9%	89.2%
True negative rate (TNR_{PRES})	22.3%	47.4%
Correct negative association rate	55.1%	30.0%
True Negative + Correct negative association rate	77.4%	77.4%
False positive rate (FPR_{PRES})	1.9%	5.8%
Incorrect positive association rate	3.5%	8.0%
False positive + Incorrect positive association rate	5.5%	13.8%
False negative rate (FNR_{PRES})	0.3%	3.1%
Incorrect negative association rate	2.0%	2.4%
False negative + Incorrect negative association rate	2.3%	5.5%

Table S31. Summary rates by training. Compare to overall rates in Table S12, Table S13, and Table S14 (see those tables for definitions).

Table S32 shows the breakdown by training group of the Error and Definitiveness categories used in Figure 4 for color-coding and symbols. A total of five participants fell in the (ideal) combination of "FPR, FNR, IAR, INR all below average" and "TPR and TNR above average" depicted as blue open circles in Figure 4B (main paper), of whom four were in Training Group A and one was in Training Group B.

Errors	*Definitiveness*	*Total*	*Training Group A 2 or more years formal training*	*Training Group B Less than 2 years formal training*
FPR,FNR,IAR,INR all below average	TPR and TNR above average	5	4	1
	TPR above, TNR below average	4	4	0
	TPR below, TNR above average	2	1	1
	TPR and TNR below average	9	7	2
Any of FPR,FNR,IAR,or INR above average	TPR and TNR above average	10	6	4
	TPR above, TNR below average	6	5	1
	TPR and TNR below average	16	14	2
FNR > 5%	TPR and TNR above average	3	0	3
	TPR and TNR above average	5	2	3
	TPR above, TNR below average	5	4	1
	TPR and TNR below average	1	1	0
FNR and FPR > 5%	TPR and TNR above average	1	0	1
FPR > 15%	TPR and TNR above average	1	0	1
	TPR above, TNR below average	1	0	1
	TPR below, TNR above average	1	0	1
Total		*70*	*48*	*22*

Table S32. Breakdown by training group of the number of participants in the Error and Definitiveness categories used in Figure 4 for color-coding and symbols. (*Examiner comparison dataset*)

Appendix K2 Associating Participant Background Attributes with Performance

The performance of 70 forensic document examiners (FDEs), each of whom completed at least 50% of all assigned samples (at least 50 QKsets), was evaluated with respect to 27 background attributes of interest (Table S33) using the methodology formally detailed in the Hicklin et. al paper on accuracy and reproducibility of bloodstain pattern analysis (25).

Because we used different performance measures for this study, as compared to (25), we developed a new proposed association reporting hierarchy:

1. If an attribute meets the criteria for all three association evaluations (variable importance analysis, Kruskal-Wallis *p*-value, and BH *q*-statistic), then the support for an association is considered notable and reported.
2. If an attribute meets the criteria for two of the association evaluations (variable importance analysis, Kruskal-Wallis *p*-value, and BH *q*-statistic), then the support for an association is considered limited and reported with qualifications.
3. Otherwise, there is insufficient support to indicate a meaningful association between the attribute and performance and no association is reported.

Table S34 details the results for variable importance analysis and significance testing of the 27 background attributes of interest, with respect to four performance measures.

Attribute	*Survey Q*	*Variable Type*	*Levels*
Age	1	Ordinal	< 40, 40-49, 50-59, 60+
Education	2	Ordinal	< Bachelors, Bachelors, Masters
Experience	3	Ordinal	<11 Years, 11+ Years
Examination frequency	4	Ordinal	Less than monthly, monthly, 1-25% of week, 26-50% of week, 51-75% of week, 76-100% of week
# times testified	6	Ordinal	0, 1-9, 10-19, 20+
Training program	7	Binary	Recommended (2+ years, no self-training, no testifying), not recommended
Formal training provider	7A/7AA	Categorical	Informal/self, US Local, US State, US Federal, US Other, Intl Gov
Testified (while in training)	9	Binary	Yes, No
Certification	8	Binary	Certified (ABFDE), certified (other), qualified, other
Last proficiency test	10	Binary	Within 1 year, not within 1 year
Casework handwriting (plurality)	11	Categorical	None, Cursive, Print, Signatures, Mixed
Conclusion scale	13	Categorical	SWGDOC 2013, other 7 level scale, other (2-6 levels)
Use of LRs	14	Binary	Yes, No
Require conclusion verification	15	Binary	Yes, No
Verification frequency	15A/B	Categorical	None, Partial, All (known), All (blind)
Photograph limitations	19	Ordinal	Yes, Sometimes, No
Same conclusions (no originals)	20	Binary	Yes, No
Veracity	21P	Binary	Never, rarely, always
Mental state and/or personality	21J/M	Categorical	Never, ever
Employer: US Federal	22	Binary	Yes, No
Employer: US State	22	Binary	Yes, No
Employer: US Local	22	Binary	Yes, No
Employer: Private Practice	22	Binary	Yes, No
Employer: Academic	22	Binary	Yes, No
Employer: Intl Gov	22	Binary	Yes, No
Employer: Other	22	Binary	Yes, No
# other FDEs	23	Ordinal	0, 1, 2-4, 5+

Table S33. Background attributes of interest with associated survey question numbers and variable information. Note that in some cases response categories appearing on the survey have been combined to ensure sufficient sample sizes for comparison (minimum of 5 for any category).

	TP-CA Ratio			TN-CN Ratio			FP-IA Ratio			FN-IN Ratio		
	VIA (>10.5)	P (<0.05)	Q (<0.10)	VIA (>7.26)	P (<0.05)	Q (<0.10)	VIA (>7.04)	P (<0.05)	Q (<0.10)	VIA (>9.37)	P (<0.05)	Q (<0.10)
Age	1.17	0.52	0.71	0.10	0.74	0.83	5.03	0.18	0.45	0.05	0.95	1.00
Education	2.49	0.20	0.40	0.15	0.58	0.72	0.26	0.15	0.45	0.06	0.54	0.84
Experience	1.28	0.12	0.36	0.09	0.55	0.72	1.17	0.13	0.45	2.52	0.09	0.49
Examination frequency	0.07	0.64	0.76	0.33	0.31	0.65	0.33	0.94	0.94	0.72	0.63	0.84
# times testified	2.91	0.12	0.36	2.38	0.21	0.56	0.31	0.45	0.71	0.34	0.63	0.84
Training program	0.27	0.09	0.36	1.30	**0.02**	0.24	**19.14**	**0.01**	**0.08**	**25.64**	**0.04**	0.28
Formal training provider	7.33	0.13	0.36	**14.37**	0.67	0.79	**14.18**	**0.01**	**0.05**	5.51	0.27	0.68
Testified (while in training)	0.09	0.28	0.50	0.24	0.08	0.44	0.45	0.66	0.73	0.08	0.46	0.83
Certification	3.41	0.06	0.36	1.42	0.14	0.48	4.97	**0.00**	**0.04**	5.13	0.22	0.68
Last proficiency test	0.06	0.52	0.71	0.12	0.96	0.96	0.21	0.32	0.57	0.08	0.85	1.00
Casework handwriting (plurality)	1.01	0.68	0.77	6.80	0.85	0.89	**11.89**	0.78	0.81	0.63	0.28	0.68
Conclusion scale	1.82	0.39	0.61	5.09	0.17	0.52	0.77	0.49	0.71	**10.47**	**0.03**	0.26
Use of LRs	0.26	**0.04**	0.36	0.11	0.34	0.65	1.05	**0.00**	**0.04**	1.73	0.28	0.68
Require conclusion verification	0.06	0.51	0.71	0.22	0.31	0.65	1.88	0.59	0.73	0.07	0.62	0.84
Verification frequency	0.06	0.92	0.92	0.08	0.43	0.72	0.25	0.53	0.71	0.15	0.31	0.69
Photograph limitations	3.82	0.21	0.40	5.79	0.06	0.43	0.25	0.66	0.73	4.06	**0.01**	**0.09**
Same conclusions (no originals)	0.09	0.63	0.76	0.21	0.49	0.72	1.99	0.61	0.73	0.07	0.16	0.62
Veracity	9.11	**0.01**	0.15	**44.90**	**0.00**	0.12	0.21	0.15	0.45	2.29	**0.01**	**0.09**
Mental state and/or personality	3.25	**0.01**	0.17	0.10	0.29	0.65	2.97	**0.00**	**0.00**	0.09	0.12	0.54
Employer: US Federal	0.10	0.17	0.38	0.11	0.14	0.48	0.23	0.22	0.45	0.11	0.61	0.84
Employer: US State	0.24	0.84	0.88	0.30	0.59	0.72	0.15	0.06	0.27	0.12	0.39	0.76
Employer: US Local	8.44	0.08	0.36	0.11	0.82	0.88	0.96	0.21	0.45	0.06	0.96	1.00
Employer: Private Practice	0.05	0.84	0.88	0.26	0.39	0.69	0.14	0.34	0.57	0.08	0.96	1.00
Employer: Academic	0.08	0.55	0.71	0.12	0.51	0.72	0.23	0.28	0.53	0.22	0.66	0.84
Employer: Intl Gov	0.21	0.12	0.36	**8.98**	**0.04**	0.35	0.24	0.20	0.45	3.26	0.33	0.69
Employer: Other	0.55	0.30	0.51	0.15	0.58	0.72	0.28	0.52	0.71	0.40	1.00	1.00
# other FDEs	0.21	0.16	0.38	0.72	0.14	0.48	0.20	0.67	0.73	0.06	0.93	1.00

Table S34. Attribute versus performance results for three association evaluations: variable importance analysis and significance testing (Kruskal-Wallis p-values and q-values). The association threshold for each measure is listed in parentheses. Cells highlighted yellow meet the association criteria for a single evaluation (of the three possible). Cells highlighted blue meet the association criteria for two of the three evaluations (limited support for association). Cells highlighted green meet the association criteria for all three evaluations (notable support for association).

Appendix K2.1 Strong Support for Association with Performance

Two background attributes exhibited strong support for association with the weighted FP-IA ratio: training program and training provider. Note that training program and training provider are highly correlated attributes, with a bias-corrected Cramer's V (26, 27) of 0.6014; (note that Cramer's V serves as a correlation coefficient for categorical variables, ranging from 0 (no relationship) to 1 (perfect association between two variables).

- Fig S18A displays the distribution of the weighted FP-IA ratio as a function of training program; note the large difference in variance between the two groups—in this scenario the Kruskal-Wallis (KW) test is measuring differences in dominance rather than median. Those who completed a training program that was at least 2 years long (and did not entail self-training and/or testifying during training) were more likely to exhibit notably <u>lower</u> rates of erroneous and incorrect associations than those who did not complete such a training program ($p = 0.0147$, $q = 0.0794$). Conversely, those who did not complete the recommended training program were more likely to report above average weighted FP+IA rates.
- Fig S18B displays the distribution of the weighted FP-IA ratio as a function of formal training provider; note the large differences in variance between none/self, international government, and the group of U.S. government agencies—in this scenario the KW test is measuring differences in dominance rather than median. Based upon a Bonferroni-adjusted post-hoc analysis, participants with no formal training or self-training generally were more likely to exhibit notably <u>higher</u> rates of erroneous and incorrect associations than those who were trained by a US state agency ($p = 0.0029$), and possibly more likely to exhibit notably <u>higher</u> rates of erroneous and incorrect associations than those who were trained by a US local or US federal agency ($p = 0.0676$ and $p = 0.1371$, respectively).

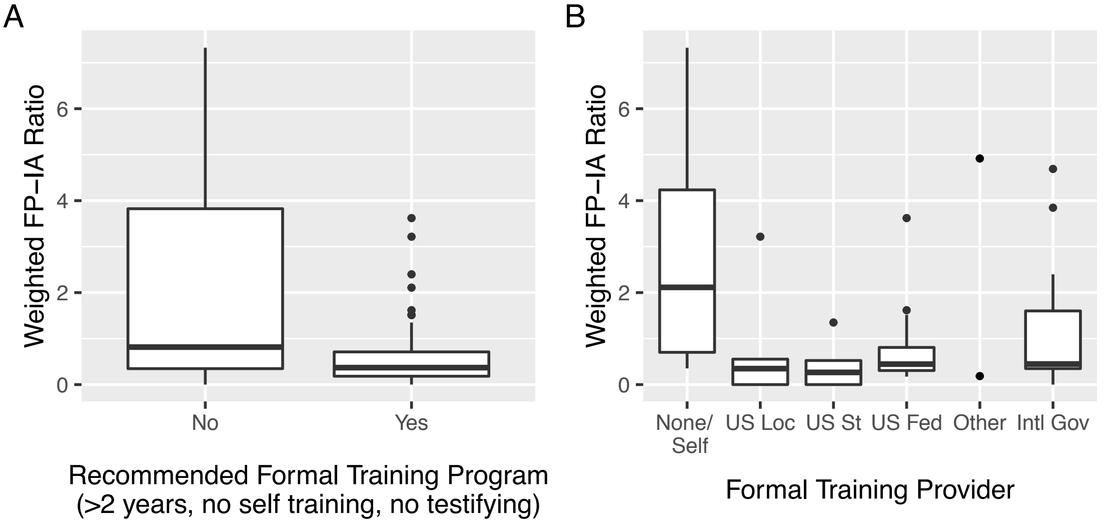

Fig S18. Distribution of weighted performance ratios for background attributes exhibiting strong support for association with performance. (N=70 participants) (Note for Formal Training Provider (plot B) that "Other" only contains 2 participants; however, all other groups contained 5 or more participants, so US Other was not combined with any other group and instead left separate)

Appendix K2.2 Limited Support for Association with Performance

In total, eight attributes exhibited limited support for association with one or more performance measures (Fig S19). The vast majority of these associations were for erroneous conclusions, with seven of the eight attributes exhibiting limited support for an association with either FP-IA or FN-IN ratio.

- Fig S19A displays the distribution of the weighted FP-IA ratio as a function of certification; note the large differences in variance between examiners who were ABFDE certified or qualified versus those who were certified by another organization or with "other" as a certification status—in this scenario the KW test is measuring differences in dominance rather than median. Based upon a Bonferroni-adjusted post-hoc analysis, participants who were qualified (not certified) appeared more likely to exhibit notably lower erroneous and incorrect association rates than those who were certified by an organization other than ABFDE ($p = 0.0290$) and those with "other" as a certification status (*e.g.,* never certified, not currently certified) ($p = 0.0464$). This same trend is also suggested for participants who were certified by ABFDE, although the evidence for it is weaker ($p = 0.1149$ and $p = 0.1400$ for those certified by another organization or with "other" as a certification status, respectively).
- Fig S19B displays the distribution of the weighted FP-IA ratio as a function of likelihood ratio (LR) use for reporting conclusions; note the large difference in interquartile range (IQR) between the two groups—in this scenario the KW test is measuring differences in dominance rather than median. The results indicated that participants who never use LRs for reporting during casework were more likely to have notably lower rates of erroneous and incorrect associations than those who do use LRs ($p = 0.0036$, $q = 0.0362$). Since this study used only a categorical conclusion scale, and did not include LRs for reporting decisions, this result may be a study design artifact. In other words, for people who typically use LRs, the reporting structure required for this study may be a departure from how they typically conduct casework— it is unclear whether this would impact their results.
- Fig S19C displays the distribution of the weighted FP-IA ratio as a function of whether the participant evaluates mental state and/or personality during casework; note the large difference in IQR between the two groups—in this scenario the KW test is measuring differences in dominance. The results indicate that participants who never evaluate mental state and/or personality were more likely to exhibit notably lower rates of erroneous and incorrect associations than participants who do so (rarely or often) ($p = 0.00003$, $q = 0.0010$).

- Fig S19D displays the distribution of the weighted FN-IN ratio as a function of training program; note the large difference in variance between the two groups—in this scenario the KW test is measuring differences in dominance. Similar to what was observed for FP-IA ratio (strong support for association), those who completed a training program that was at least 2 years long (and did not entail self-training and/or testifying during training) appear more likely to exhibit notably lower rates of erroneous and incorrect nonassociations than those who did not complete such a training program ($p = 0.0414$, $q = 0.2796$). However, the support for this association is limited and should be interpreted with caution owing to the *q*-value exceeding the acceptable false discovery level (0.10).
- Fig S19E displays the distribution of the weighted FN-IN ratio as a function of conclusion scale. Based upon a Bonferroni-adjusted post-hoc analysis, participants who use a conclusion scale with 2-6 levels for reporting during casework have notably lower rates of erroneous and incorrect nonassociations than those who use a 7-level conclusion scale ($p = 0.0248$); however, the support for this association is limited and should be interpreted with caution owing to the *q*-value exceeding the acceptable false discovery level (0.10).
- Fig S19F displays the distribution of the weighted FN-IN ratio as a function of whether conclusions are limited when examining photocopies/photographs only. Based upon a Bonferroni-adjusted post-hoc analysis, participants who always limit their conclusions when examining photocopies/photographs had notably lower rates of erroneous and incorrect nonassociations than those who sometimes limited their conclusions ($p = 0.0064$). This result is likely explained by differences in reporting tendencies between these two groups: participants who sometimes limited their conclusions tended to be more definitive than those who always did (median: 47.8% vs 28.4% definitive conclusions, respectively— thereby increasing the chances for an erroneous nonassociation. There was no difference detected between those who do not limit their conclusions and the other groups, although this may be a sample size artifact (n=4 participants).
- Fig S19G displays the distribution of the weighted FN-IN ratio as a function of whether the participant evaluates veracity during casework; note the large differences in variance between those who rarely report on veracity versus those who often or never do—in this scenario the KW test is measuring differences in dominance rather than median. Based upon a Bonferroni-adjusted post-hoc analysis, participants who never assess veracity are more likely to exhibit notably lower rates of erroneous and incorrect nonassociations than those who rarely do ($p = 0.0069$). They also suggest that participants who never evaluate veracity are generally less definitive than those who rarely do (median: 30.0% vs 64.4% definitive conclusions, respectively) and also more indeterminate in reporting conclusions (median: 12.2% vs 1.1% inconclusive determinations, respectively).

Two additional associations were detected with respect to rates of correct conclusions, with two attributes exhibiting limited support for an association with weighted TN-CN ratio. We did not detect evidence of an association between any of the background attributes and TP-CA ratio.

- Fig S19H displays the distribution of the weighted TN-CN ratio as a function of whether the participant evaluates veracity during casework. Based upon a Bonferroni-adjusted post-hoc analysis, participants who never assess veracity have notably lower rates of true positives and correct nonassociations than those who rarely do ($p = 0.0256$) and possibly lower TN-CN ratios than those who often do ($p = 0.0552$). This result reinforces the observation made for FN-IN ratios, wherein a similar relationship was noted: those who never evaluate veracity have lower FN-IN ratios than those who rarely do. Both results are likely linked to reporting tendencies, wherein people who never assess veracity are generally less definitive in reaching conclusions.
- Fig S19I displays the distribution of the weighted TN-CN ratio as a function of whether the participant has ever worked for an international government agency. The results indicate that participants who have worked for an international government agency may have higher rates of true negative and correct nonassociations than those who have not worked for such an agency ($p = 0.0393$, $q = 0.3535$). However, the support for this association is limited and should be interpreted with caution owing to the *q*-value exceeding the acceptable false discovery level (0.10).

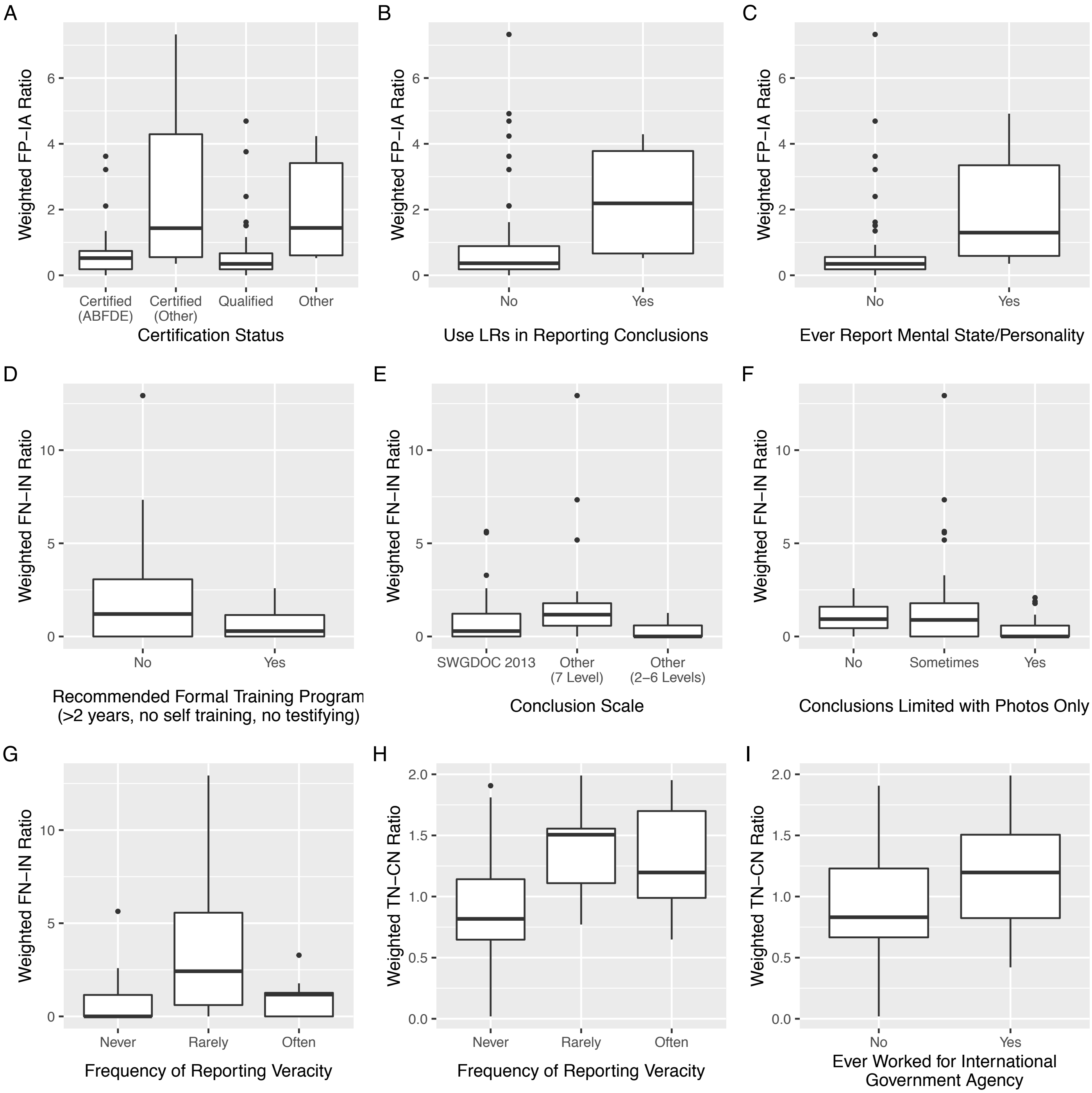

Fig S19. Distribution of weighted performance ratios for background attributes exhibiting limited support for association with performance. (N=70 participants) (Note that only four participants indicated that they do not limit conclusions when examining photocopies/photographs (plot F))

Appendix L Writing style

As described in Section 4.9 (main paper), there is interest in assessing the accuracy and reliability of FDE conclusions by writing style. To do so, the writing style of the Q in each QKset was assessed by each participant using a five-category scale ranging from disconnected printing to connected cursive (see *Appendix D6* for details). Fig S20 depicts the assessment of writing style by participants for all 180 QKsets. Table S35 quantifies the number of trials and QKsets by writing style. Using a majority of participants to assess the writing style of the Q in each QKset, 37% were disconnected printing, 29% were connected cursive, and the remaining 34% were considered "mixed".

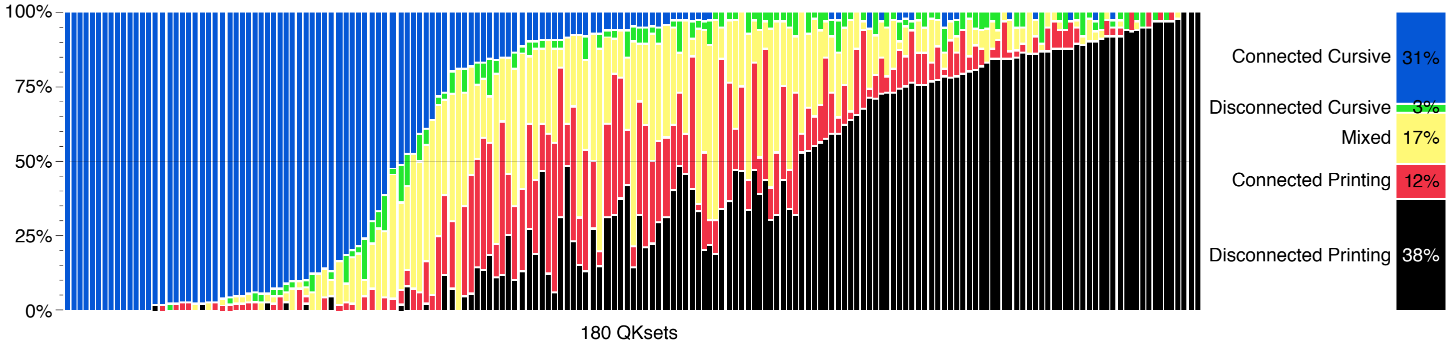

Fig S20. Assessments of writing style of the Q in each QKset. X axis: 180 distinct QKsets sorted by percentage of examiners assessing the Q as "Connected cursive." Y axis: proportion of responses listing each writing style. Few Qs were unanimously classified: 3 were unanimously Disconnected print, and 14 were unanimously Connected Cursive. (*Baseline Dataset*)

	Trials		*QKsets (plurality)*		*QKsets (majority)*	
DisconnPrint	2475	38%	76	42%	66	37%
ConnectedPrint	773	12%	21	12%		
Mixed	1117	17%	29	16%	62	34%
DisconnCursive	204	3%	0	0%		
ConnectedCursive	2007	31%	54	30%	52	29%
Total	6576		180		180	

Table S35. Assessments of writing style: overall distribution of assessments with plurality and majority assessments. Majorities were assessed as "mixed" for any QKsets in which the Q was not majority "disconnected print" or "connected cursive".

Appendix L1 Conclusions by writing style

At least one earlier study has evaluated performance conditioned upon writing style (13); the large number and variety of writing samples included in this study facilitates a similar assessment of conclusions as a function of writing style. However, it is necessary to acknowledge that such an assessment is inherently confounded by a variety of study-related and sample-related factors, including mate prevalence with each writing style, dataset or origin, and comparability of content. For this study, we specifically sought to determine whether errors rates varied as a function of writing style; Table S36 details conclusion rates as a function of writing style. We did not detect support for differences in error rates (FPR_{PRES}, FNR_{PRES}) or incorrect conclusion rates (IAR_{PRES}, INR_{PRES}) as a function of writing style. Moreover, conclusion rates overall (irrespective of correctness) did not vary as a function of writing style for nonmated trials. (All statistical tests in this section were based upon a chi-square analysis, with Bonferroni-adjusted Pearson residual post-hoc testing.)

In contrast, we did detect differences in conclusion rates for mated trials: conclusions reported for cursive writing were much more conservative than those reported for mixed or print samples (note that there was no significant difference in rates for mixed vs print writing). To further understand what might be contributing to this result, we

partitioned the chi-square analysis based upon dataset and content comparability. The justifications for this partitioning as well as the results of interest are as follows:

- Dataset: the NARA dataset for mated trials was entirely composed of cursive writing (no mixed or print samples), so we wanted to determine whether this was disproportionately impacting the observed differences in conclusion rates for cursive versus mixed/print samples.
    - Conclusion rates did not differ between cursive writing for the NARA dataset and cursive writing for the other datasets.
- Content comparability (Table S37): given the limitations of the study (time-restricted, limited amount of questioned/known writing, digital images only), we wanted to determine whether content comparability was positively or negatively (depending upon same/different content) impacting the observed differences in conclusion rates.
    - Conclusion rates did not differ as a function of writing style for comparisons involving different content. Participants were approximately equally conservative/decisive when reporting decisions for different content trials.
    - Conclusion rates differed as a function of writing style for comparisons involving same content. In particular, mixed and print writing exhibited a large increase in definitive conclusions when presented with the same content for comparison, whereas cursive samples did not yield such an inflation. Cursive trials thus were more likely to result in a more conservative decision (*ProbWritten* or even *NoConc*) than trials containing mixed or printed writing, which were more likely to yield a conclusion of *Written*.

In summary, the results overall indicate that there was not support for differences in error rates or incorrect decision rates as a function of writing style, irrespective of partitioning. Furthermore, conclusion rates did not differ as a function of writing style for nonmated trials and for trials involving mixed or print writing, again irrespective of partitioning. However, we did detect notable differences in conclusion rates for mated pairs; these differences seem to be largely explained by the differences detected between trials presenting same/different content. When comparisons involve different content, participants were equally conservative/decisive across all three writing styles; however, when comparing Q and K writing that was the same content, participants became much more decisive (increased proportion of *Written*) for mixed and print writing than for cursive writing.

		Mated						*Nonmated*					
	Trials	*Cursive*		*Mixed*		*Print*		*Cursive*		*Mixed*		*Print*	
Written	1660	397	43%	753	59%	396	60%	34	3%	21	2%	59	4%
ProbWritten	1161	361	39%	410	32%	211	32%	63	6%	49	5%	67	4%
NoConc	790	124	13%	82	6%	37	6%	163	16%	137	14%	247	15%
ProbNot	1834	31	3%	23	2%	6	1%	468	46%	472	47%	834	49%
NotWritten	1131	14	2%	12	1%	6	1%	289	28%	321	32%	489	29%
Total	6576	927		1280		656		1017		1000		1696	

Table S36. Conclusions by writing style, as assessed by the majority of respondents. (*Baseline Dataset*)

	Same Content						*Different Content*						*Total*					
	Cursive		*Mixed*		*Print*		*Cursive*		*Mixed*		*Print*		*Cursive*		*Mixed*		*Print*	
Written	136	50%	545	68%	295	68%	261	40%	208	44%	101	45%	397	43%	753	59%	396	60%
ProbWritten	102	37%	215	27%	121	28%	259	40%	195	41%	90	40%	361	39%	410	32%	211	32%
NoConc	21	8%	32	4%	11	3%	103	16%	50	11%	26	12%	124	13%	82	6%	37	6%
ProbNot	7	3%	8	1%	3	1%	24	4%	15	3%	3	1%	31	3%	23	2%	6	1%
NotWritten	7	3%	4	0%	1	0%	7	1%	8	2%	5	2%	14	2%	12	1%	6	1%

Table S37. Conclusion rates on mated comparisons as a function of content. (Mated subset of *Baseline Dataset*)

Appendix M Effect of Participants' Assessments of Difficulty

Each participant assessed the difficulty of each comparison using this scale:

- Obvious (Very Easy (VEasy)): The conclusion was obvious.
- Easy: The comparison was easier than most comparisons.
- Moderate: The comparison was typical of most comparisons.
- Difficult: The comparison more difficult than most comparisons.
- Very Difficult (VDifficult): The comparison was unusually difficult.

The distribution of difficulty assessments is shown in Table S38: the responses overall are relatively balanced, but the mated comparisons were rated significantly easier than nonmated according to a chi-square analysis (with Bonferroni-adjusted Pearson residual post-hoc testing). This result could support the observation that FDEs consider exclusions as more difficult than inclusions.

	Total		Mates		Nonmates	
VDifficult	269	4.1%	83	2.9%	186	5.0%
Difficult	1284	19.5%	400	14.0%	884	23.8%
Moderate	2805	42.7%	1095	38.2%	1710	46.1%
Easy	1793	27.3%	960	33.5%	833	22.4%
VEasy	425	6.5%	325	11.4%	100	2.7%

Table S38. Distribution of difficulty as assigned by participants. (*Baseline Dataset*)

The distribution of responses is strongly associated with the difficulty of the comparison, as shown in Fig S21. Overall, 88.0% of VEasy comparisons were definitive conclusions, 12.0% were qualified, and none were inconclusive. This contrasts with the VDifficult comparisons, in which 9.7% were definitive, 39.8% were qualified, and 50.6% were inconclusive. Note that a majority of erroneous *Written* conclusions were assessed as Easy or VEasy: of the 114 FPs, 14 were assessed as VEasy, 46 were Easy, 45 were Moderate, 5 were Difficult, and 4 were VDifficult. By contrast, erroneous *NotWritten* assessments were balanced with respect to difficulty: 1 VEasy, 7 Easy, 17 Moderate, 6 Difficult, and 1 VDifficult.

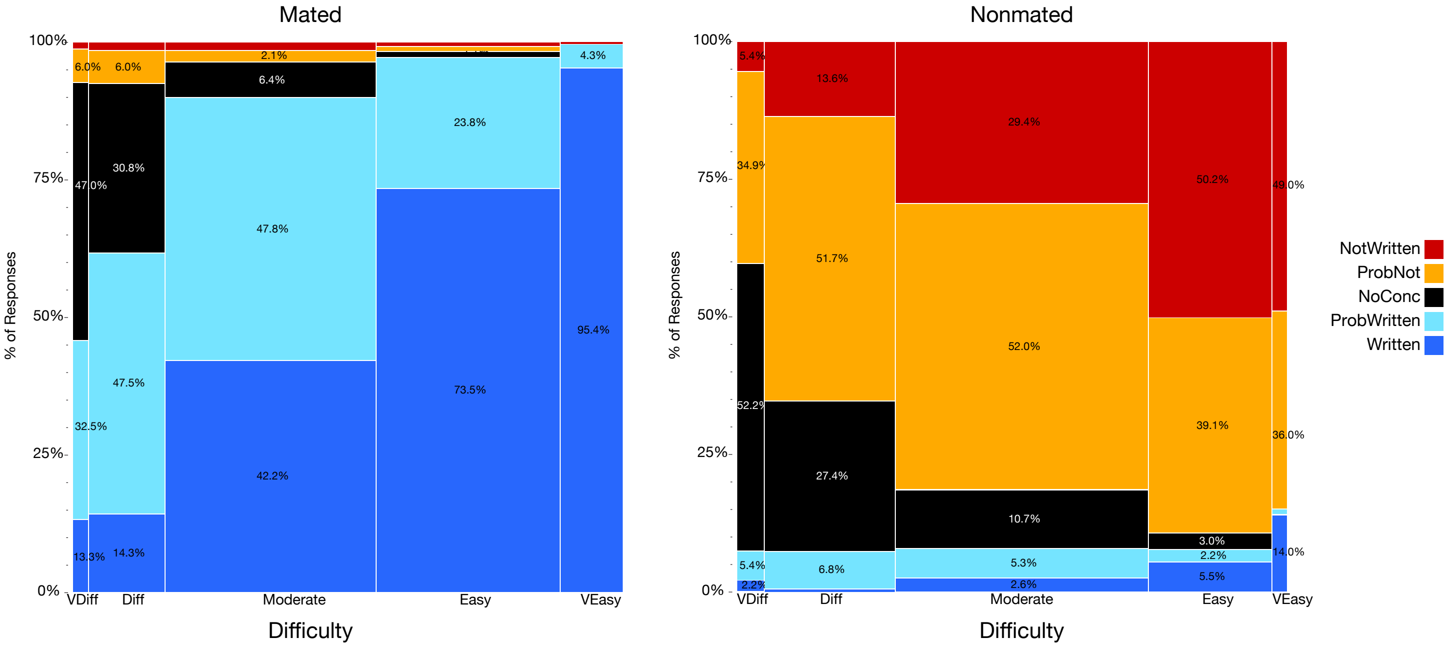

Fig S21. Conclusion rates by difficulty. (*Baseline Dataset*)

Of the 86 participants, 45 made at least one erroneous or incorrect response that they assessed as Easy or VEasy; an additional 26 participants made at least one erroneous or incorrect response that they assessed as Moderate. Of

the 70 participants in the *Examiner comparison dataset*, four participants had 5% or more of responses that were erroneous or incorrect yet assessed as Easy or VEasy; an additional 12 participants had 5% or more of responses that were erroneous or incorrect yet assessed as Easy, VEasy, or Moderate.

In the post-test survey (Appendix C2), participants assessed the overall difficulty of the comparisons: "How does the overall difficulty of the comparisons in this study correspond to your casework?" Of the 66 participants who completed the post-test survey, 12% indicated "Easier", 80% "Similar", and 8% "Harder." These overall assessments of difficulty may be debatable given that some participants made incorrect responses on comparisons they considered easy. However, if we ignore participants for whom 5% or more of responses were erroneous or incorrect yet assessed as Easy, VEasy, or Moderate, the overall assessments are not notably different: of the remaining 51 participants, 12% indicated "Easier", 82% "Similar", and 6% "Harder."

References for Appendices

1. C. P. Saunders, L. J. Davis, J. Buscaglia, Using Automated Comparisons to Quantify Handwriting Individuality. *J. Forensic Sci.* **56** (2011).
2. S. N. Srihari, C. Huang, H. Srinivasan, On the Discriminability of the Handwriting of Twins. *J. Forensic Sci.* **53**, 430–446 (2008).
3. T. Dziedzic, E. Fabianska, Z. Toeplitz, Handwriting of Monozygotic and Dizygotic Twins. *Probl. Forensic Sci.* **69**, 30–36 (2007).
4. D. Boot, An Investigation into the Degree of Similarity in the Handwriting of Identical and Fraternal Twins in New Zealand. *J. Am. Soc. Quest. Doc. Exam.* **1**, 70–81 (1998).
5. M. S. Beacom, A Study of Handwriting by Twins and Other Persons of Multiple Births. *J. Forensic Sci.* **5**, 121–131 (1960).
6. D. Gamble, The handwriting of Identical Twins. *Can. Soc. Forensic Sci. J.* **13**, 11–30 (1980).
7. C. Monden, G. Pison, J. Smits, Twin Peaks: more twinning in humans that ever before. *Hum. Reprod.* **36** (2021).
8. S. N. Srihari, S.-H. Cha, H. Arora, S. Lee, "Individuality of Handwriting" (2001).
9. B. T. Ulery, R. A. Hicklin, J. Buscaglia, M. A. Roberts, Accuracy and reliability of forensic latent fingerprint decisions. *Proc. Natl. Acad. Sci. U. S. A.* **108** (2011).
10. M. Kam, J. Wetstein, R. Conn, Proficiency of Professional Document Examiners in Writer Identification. *J. Forensic Sci.* **39**, 5–14 (1994).
11. M. Kam, G. Fielding, R. Conn, Writer Identification by Professional Document Examiners. *J. Forensic Sci.* **42**, 778–786 (1997).
12. M. Durina, M. Caligiuri, The Determination of Authorship from a Homogenous Group of Writers. *J. Am. Soc. Quest. Doc. Exam.* **12**, 77–90 (2009).
13. M. Kam, E. Lin, Writer identification using hand-printed and non-hand-printed questioned documents. *J. Forensic Sci.* **48**, 1391–1395 (2003).
14. L. L. Mitchell, M. Merlino, A Blind Study on the Reliability of Hand Printing Identification by Forensic Document Examiners. *J. Am. Soc. Quest. Doc. Exam.*, 25–31 (2016).
15. M. Kam, K. Gummadidala, G. Fielding, R. Conn, Signature Authentication by Forensic Examiners. *J. Forensic Sci.* **46**, 884–888 (2001).
16. J. Sita, B. Found, D. K. Rogers, Forensic Handwriting Examiners' Expertise for Signature Comparison. *J. Forensic Sci.* **47**, 1–8 (2002).
17. President's Council of Advisors on Science and Technology (PCAST), "Report to the President. Forensic Science in Criminal Courts: Ensuring Scientific Validity of Feature-Comparison Methods" (Executive Office of the President, 2016).
18. OSAC Human Factors Committee, "Draft Guidance on Testing the Performance of Forensic Examiners" (2018).
19. OSAC Human Factors Committee, "Human Factors in Validation and Performance Testing of Forensic Science (OSAC Technical Series 0004)" (2020) https:/doi.org/https://doi.org/10.29325/OSAC.TS.0004.
20. A. Agresti, B. A. Coull, Approximate Is Better than "Exact" for Interval Estimation of Binomial Proportions. *Am. Stat.* **52**, 119–126 (1998).
21. G. W. Corder, D. I. Foreman, *Nonparametric Statistics: A step-by-step approach* (John Wiley & Sons, Inc., 2014).
22. Scientific Working Group for Forensic Document Examination (SWGDOC), SWGDOC Standard for Minimum Training Requirements for Forensic Document Examiners (2013).
23. ANSI National Accreditation Board (ANAB), Guiding Principles of Professional Responsibility for Forensic Service Providers and Forensic Personnel (2018).
24. Cornell Statistical Consulting Unit, Using Adjusted Standardized Residuals for Interpreting Contingency Tables. *Statnews #95* (2020).
25. R. A. Hicklin, *et al.*, Accuracy and Reproducibility of Conclusions by Forensic Bloodstain Pattern

Analysts. *Forensic Sci. Int.* **325** (2021).
26. D. Cramer, D. Howitt, "Cramer's V" in *The SAGE Dictionary of Statistics*, (SAGE Publications, Inc., 2011) https:/doi.org/https://dx.doi.org/10.4135/9780857020123.
27. W. Bergsma, A bias-correction for Cramér's and Tschuprow's. *J. Korean Stat. Soc.* **42** (2013).
